# Comparative transcriptomics sheds light on differential adaptation and species diversification between two *Melastoma* species and their F_1_ hybrid

**DOI:** 10.1093/aobpla/plz019

**Published:** 2019-03-28

**Authors:** Wei Lun Ng, Wei Wu, Peishan Zou, Renchao Zhou

**Affiliations:** 1 State Key Laboratory of Biocontrol and Guangdong Provincial Key Laboratory of Plant Resources, School of Life Sciences, Sun Yat-sen University, Guangzhou, Guangdong, China; 2 China-ASEAN College of Marine Sciences, Xiamen University Malaysia, Sepang, Selangor, Malaysia

**Keywords:** Adaptive divergence, comparative transcriptomics, *Melastoma*, transgressive segregation, trichome

## Abstract

Variation in gene expression has been shown to promote adaptive divergence, and can lead to speciation. The plant genus *Melastoma*, thought to have diversified through adaptive radiation, provides an excellent model for the study of gene expressional changes during adaptive differentiation and following interspecific hybridization. In this study, we performed RNA-seq on *M. candidum*, *M. sanguineum* and their F_1_ hybrid, to investigate the role of gene expression in species diversification within the genus. Reference transcriptomes were assembled using combined data from both parental species, resulting in 50 519 and 48 120 transcripts for the leaf and flower petal, after removing redundancy. Differential expression analysis uncovered 3793 and 2116 differentially expressed (DE) transcripts, most of which are between *M. candidum* and *M. sanguineum*. Differential expression was observed for genes related to light responses, as well as genes that regulate the development of leaf trichomes, a trait that among others is thought to protect plants against sunlight, suggesting the differential adaptation of the species to sunlight intensity. The analysis of positively selected genes between the two species also revealed possible differential adaptation to other abiotic stresses such as drought and temperature. In the hybrid, almost all possible modes of expression were observed at the DE transcripts, although at most transcripts, the expression levels were similar to that of either parent instead of being intermediate. A small number of transgressively expressed transcripts that matched genes known to promote plant growth and adaptation to stresses in new environments were also found, possibly explaining the vigour observed in the hybrid. The findings in this study provided insights into the role of gene expression in the diversification of *Melastoma*, which we believe is an important example for more cross-taxa comparisons in the future.

## Teaser

Variation in gene expression promotes adaptive divergence. In this study, we performed comparative transcriptomics on *Melastoma candidum*, *M. sanguineum* and their F_1_ hybrid to investigate the role of gene expression in plant species diversification and hybridization. Differentially expressed genes were mostly found between the two parental species, and were related to adaptive traits that limit the species to their habitats. In the F_1_ hybrid, although the expression levels of most genes were similar to either parent, a small number of genes had expression levels exceeding both parents, possibly explaining its vigour in certain morphological and adaptive traits.

## Introduction

In speciation, the formation of new lineages can be driven by two basic evolutionary processes—lineage divergence and lineage fusion. Lineage divergence can happen through differential adaptation or genetic drift, with the former expected to proceed more rapidly when the effective population size is large (Jensen and Bachtrog 2011). Lineage fusion through hybridization, on the other hand, can provide raw material for adaptation to various habitats by creating new allelic combinations and phenotypes (Arnold *et al.* 2012). When the two drivers of speciation take effect on a single group of taxa, rapid species radiation can occur, as seen in the cichlid fishes of Africa (Meier *et al.* 2017) and the kiwifruit (Liu *et al.* 2017).

While the majority of studies on lineage divergence has focused on sequence or structural variation (e.g. Turner *et al.* 2005; Schluter and Conte 2009; Hendrick *et al.* 2016; Lindtke *et al.* 2017), variation in gene expression has also been shown to be relevant in speciation (e.g. Wolf *et al.* 2010). Its interaction with the environment can promote adaptive divergence—when a population colonizes a new environment, the regulation of gene expression becomes critical in ensuring the persistence of a population. In time, this also promotes genetic divergence in adaptive traits, eventually causing reproductive isolation between populations (Pavey *et al.* 2010)—and this can have profound impacts on the diversification of species. A similar impact can be attained following lineage fusion: in hybridization, a first-generation (F_1_) hybrid receives half of its genetic material from each of its parents. The resulting gene expressional patterns may or may not be additive of that observed in the parental taxa (Birchler *et al.* 2003). Transcriptomic shock, usually presented in the form of the complete suppression of transcripts from one parent and widespread up- or down-regulation of gene expression (Hegarty 2011; Barreto *et al.* 2015), can result in novel phenotypes or even transgressive phenotypes that exceed that of the parents (i.e. transgressive segregation; deVicente and Tanksley 1993; Rieseberg *et al.* 1999; Rieseberg *et al.* 2003). These attributes, given the right environment and intrinsic compatibility, may then lead to rapid establishment of the hybrid population into an independent evolutionary lineage (Abbott *et al.* 2013).

**Figure 1.**
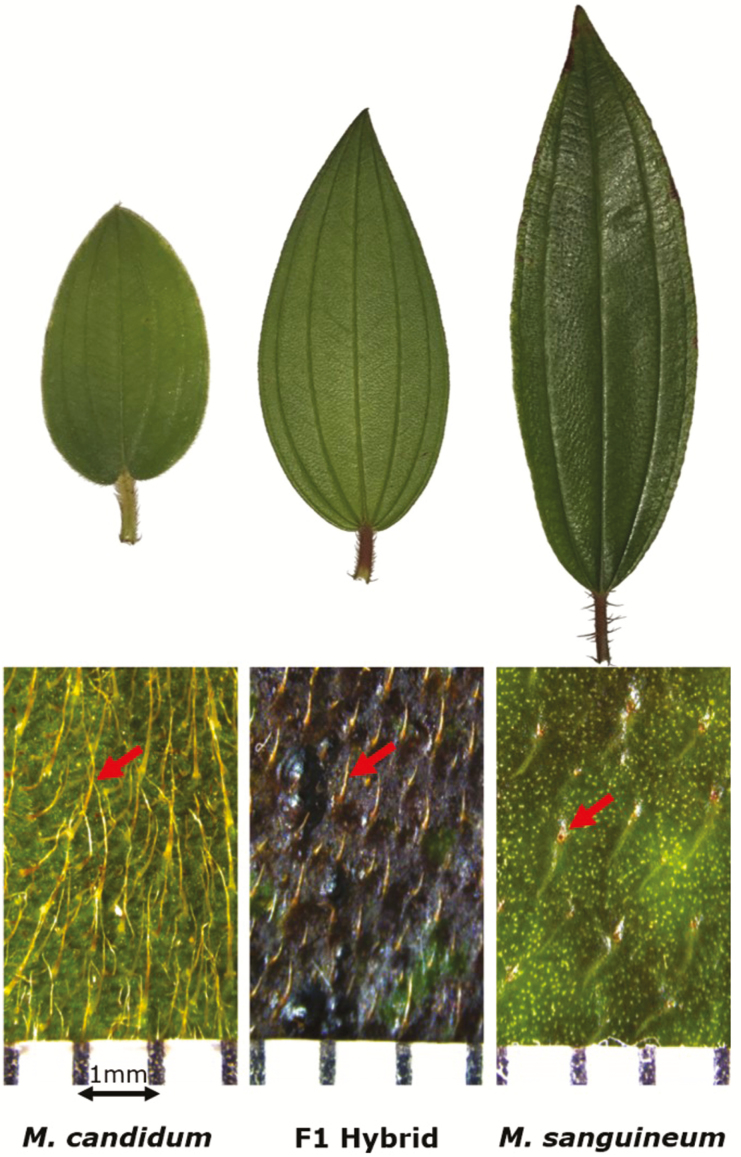
Leaf morphology (top) and trichome structure observed under ×10 magnification (bottom) for *M. candidum*, *M. sanguineum* and their F_1_ hybrid. Differences in the length and density of trichome present on the leaf surface partly contribute to the difference in visible coloration of the leaves.

The shrub genus *Melastoma* (Melastomataceae) provides an excellent model to study the gene expressional changes during adaptive differentiation and following interspecific hybridization. *Melastoma* is thought to have diversified through adaptive radiation (Renner and Meyer 2001), with many species within the genus having evolved to fit into different ecological niches, such as occupying lowlands vs. montane elevations and open vs. shady environments (Wong 2016). At the same time, their recent divergence (i.e. 1 million years ago; Renner and Meyer 2001) and shared life history characteristics, such as partially overlapping geographic distribution and flowering periods (Chen 1984), as well as shared pollinators (Gross 1993; Luo *et al.* 2008), allow hybridization to happen easily between the co-occurring species. Two species, *M. candidum* and *M. sanguineum*, are sympatrically distributed in southern China and hybridize extensively (Liu *et al.* 2014). Despite the largely shared geographical distribution, they have different habitat preferences: in the wild, *M. candidum* prefers exposed environments such as open fields, grasslands and roadsides, while *M. sanguineum* prefers shady environments and is often found at the edges of forests. This adaptation to different sunlight intensities seems to be related to the presence and absence of trichomes (hair-like structures) on the surface of their mature leaves (Fig. 1). Of their various functions, trichomes are thought to play a role in protection of the leaves from sunlight (Wagner *et al.* 2004; Hauser 2014), and thus may hold the key to adaptive divergence between the two *Melastoma* species. Their F_1_ hybrid, on the other hand, displays trichome length intermediate between its parents, while having more vigorous growth (e.g. bigger leaves and flowers, faster growth) and seem capable of crossing over adaptation barriers (e.g. sunlight exposure) that limit the parental species to their habitats (W. L. Ng and R. Zhou, pers. obs.). In fact, during a survey at the Diaoluo Mountain in Hainan, China, we observed hybrid individuals that flourished in constant-shaded habitats and at higher altitudes compared to the parents. Such displays of both intermediate and transgressive phenotypes in the hybrids demonstrate the possible role of gene expressional regulation following hybridization.

Earlier studies that use transcriptomic data to compare gene expression in plants have mostly looked at gene expressional changes within a single species subjected to different treatments (e.g. Carvajal *et al.* 2018) or following hybridization of different varieties/ecotypes of the same species (e.g. Shen *et al.* 2012; Bell *et al.* 2013; He *et al.* 2013). Few have actually looked at differences between divergent species, presumably due to the complexity of comparing large-scale gene expression across species (Wolf *et al.* 2010; Kristiansson *et al.* 2013; Roux *et al.* 2015; Shimizu-Inatsugi *et al.* 2016). Our study therefore aimed to look further to compare gene expression between two plant species, and the expressional changes following hybridization, using *M. candidum*, *M. sanguineum* and their F_1_ hybrid, as a model system. Such comparisons can elucidate the molecular mechanisms that allow for differential adaptation of the taxa to differing habitats, which could be a major underlying cause for species radiation within the genus *Melastoma*.

## Materials and Methods

### Sampling and RNA extraction

Two individuals (biological replicates) each of *M. candidum*, *M. sanguineum* and their F_1_ hybrid (‘hybrid’) were grown in the greenhouse at Sun Yat-sen University, Guangzhou, China, under a white shade cloth with a low level of shade (i.e. 20 %), during which all individuals showed normal growth. The individuals were initially collected from Qiongzhong, Hainan, China. The F_1_ hybrid status of the hybrid individuals was confirmed by sequencing six nuclear loci used in our previous studies on *Melastoma* hybrid identification (Dai *et al.* 2012; Zhou *et al.* 2017; Zou *et al.* 2017). Samples of two tissue types, i.e. leaf and flower petal (‘petal’), were harvested at 0900 h in the morning of 30 June 2017, when the petals exhibited complete opening, snap-frozen in liquid N_2_ and stored at −80 °C until RNA extraction. Total RNA was extracted using the RNAprep Pure Plant Kit (polysaccharides and polyphenolics-rich; TIANGEN Biotech Co. Ltd, Beijing, China). Voucher specimens of the samples used in this study (NG201801–NG201806) were deposited in the Herbarium of Sun Yat-sen University (SYS).

### RNA-seq and data preprocessing

Upon RNA integrity checking and quantification (integrity of RNA samples were measured with an Agilent 2100 Bioanalyzer and RIN (RNA Integrity Number) of >7 is required), the total RNA samples were subjected to library construction (insert size ~450 bp, strand-specific using the dUTP protocol; Parkhomchuk *et al.* 2009) and Illumina HiSeq sequencing (150 bp paired-end). The quality of the obtained raw sequencing reads was first examined using FastQC (Babraham Bioinformatics; www.bioinformatics.babraham.ac.uk/projects/fastqc/) ver. 0.11.3. Then, a series of quality filtering steps were conducted on the raw reads, including the removal of adapter sequences, removal of reads containing >5 % of N bases, trimming of bases with Phred Q score <20 (i.e. error rate ≥1 %) and removal of sequences with average Phred Q score <20. Only paired-end reads with length ≥50 bp were kept. All filtering steps were conducted using BBDuk embedded in BBTools (jgi.doe.gov/data-and-tools/bbtools) ver. 37.76. The quality-filtered reads were considered high-quality (‘HQ’) and were used for the subsequent assembly and mapping steps.

### *De novo* assembly and redundancy reduction

The Trinity (Grabherr *et al.* 2011; Haas *et al.* 2013) pipeline for genome-guided *de novo* assembly was used to assemble transcripts from the RNA-seq data. In order to conduct analysis on differential expression among *M. candidum*, *M. sanguineum* and the hybrid, a common set of reference transcripts had to be generated. The common set of reference transcripts were obtained by generating a ‘master’ transcriptome assembled from reads from both parental species. All four HQ data sets of *M. candidum* and *M. sanguineum* (two replicates per species, each tissue type treated separately) were first combined and normalized using the Trinity script *insilico_read_normalization.pl* [max_cov = 50, pairs_together, SS_lib_type = RF]. Then, the normalized reads were aligned to a *M. candidum* draft genome (W. Wu *et al*., unpubl. data) using the STAR aligner (Dobin *et al.* 2013) ver. 2.5.3, prior to *de novo* assembly using Trinity ver. 2.5.1.

To reduce redundancy, the *tr2aacds* pipeline of the EvidentialGene package (Gilbert 2016) ver. 2017.03.10 was used to process all the Trinity-assembled transcripts. Leveraging the functions of CD-HIT (Fu *et al.* 2012), Exonerate (Slater and Birney 2005) and NCBI’s BLAST, the pipeline trims down the set of assembled transcripts and categorizes the transcripts into ‘okay’, ‘okay-alternate’ and ‘drop’ sets, based on coding potential. The ‘okay’ sets of transcripts were regarded as optimal and hence as the representative transcript sets, i.e. the transcriptomes.

For comparison of transcriptomic properties across *M. candidum*, *M. sanguineum* and the hybrid, the transcriptomes of each tissue type of *M. candidum*, *M. sanguineum* and the hybrid were also separately assembled. To do this, RNA-seq data of both replicates of a single tissue type from each of *M. candidum*, *M. sanguineum* and the hybrid were combined, normalized, assembled and trimmed down, as described above. For convenience, the two types of transcriptomes assembled in this study, i.e. the transcriptomes assembled from combined *M. candidum* and *M. sanguineum* RNA-seq data, and the transcriptomes assembled from RNA-seq data of *M. candidum*, *M. sanguineum* or the hybrid samples, shall hereinafter be known as the ‘master transcriptomes’ and the ‘taxon-specific transcriptomes’, respectively.

### Quality assessment of assembled transcriptomes

To assess the quality of the transcriptomes, TransRate (Smith-Unna *et al.* 2016) ver. 1.0.3 was used to conduct a reference-free quality assessment of the assembled transcriptomes. It generates basic metrics that describe a transcript set, and maps the HQ reads back to the transcriptomes to estimate various metrics from the alignment.

The transcriptomes were compared to a set of 956 core plant genes using the Benchmarking Universal Single-Copy Ortholog (BUSCO) assessment tool (Simao *et al*. 2015) ver. 3.0.2 to assess the completeness of the transcriptome. Contigs were categorized as ‘complete, single copy’, ‘complete, duplicated copy’, ‘fragmented’ or ‘missing’, depending on the length of the aligned sequence. The BUSCO analysis [lineage = plantae, mode = transcriptome, e-value ≤ 1e-3] was run on the Cyverse Discovery Environment (de.cyverse.org; accessed March 2018).

The number of full-length coding transcripts present in the transcriptomes were also used as an assessment of assembly quality. This was done by comparing the transcriptomes to the Viridiplantae protein database of UniProtKB/Swiss-Prot using BLASTx [e-value ≤ 1e-20, max_target_seqs = 1]. The number of transcripts estimated to be full-length (>90 %) or nearly full-length (>70 %) were counted using the Trinity script *analyze_blastPlus_topHit_coverage.pl*.

### Differential expression and functional enrichment

To quantify the expression abundance of each transcript, the Trinity script *align_and_estimate_abundance.pl* was used, employing Kallisto (Bray *et al.* 2016) to generate normalized abundance estimates across transcripts of the master transcriptomes. EdgeR (Robinson *et al.* 2010) was subsequently used to identify differentially expressed (DE) transcripts (with Trinity script *run_DE_analysis.pl*), with those satisfying at the same time a false discovery rate (FDR) of ≤0.001 and a log2 fold-change (logFC) of ≥2 (as suggested by Vijay *et al.* 2013) considered as DE. Heatmaps displaying the relative expression of DE transcripts and expression profile clustering were generated using the Trinity scripts *analyze_diff_expr.pl* and *define_clusters_by_cutting_tree.pl* [Ptree = 60], respectively. Then, DE transcripts were extracted and queried against the *Arabidopsis thaliana* protein database using KOBAS ver. 3.0 (Xie *et al.* 2011) to identify enriched Kyoto Encyclopedia of Genes and Genomes (KEGG) and Gene Ontology (GO) terms [e-value ≤ 1e-5]. The definitions of GO terms were referenced from AmiGO 2 (amigo.geneontology.org).

### Identification of DE genes related to trichome development

The presence of trichomes on the leaves is thought to influence differential adaptation of plant species to sunlight. Among *M. candidum*, *M. sanguineum* and the hybrid, leaf trichome length is greatest in *M. candidum*, followed by the hybrid (intermediate length), and *M. sanguineum* (shortest, but often absent). To determine the molecular factors behind the two extreme leaf trichome phenotypes (presence and absence), we looked at DE transcripts between *M. candidum* and *M. sanguineum* in the leaf that could be related to trichome development. First, *A. thaliana* TAIR10 proteins (ver. 20101214) with functional descriptions containing the keyword ‘trichome’ were extracted from the TAIR10 functional description list (ver. 20170331). Differentially expressed transcripts between *M. candidum* and *M. sanguineum* identified from the leaf master transcriptome were then queried against these trichome-related protein sequences using BLASTx [e-value ≤ 1e-3], and significant matches (best-hit only, bit score ≥ 50, % identity ≥ 30; as suggested by Pearson 2013) were recorded. The same procedures were conducted on DE transcripts between *M. candidum* and *M. sanguineum* in the petal as control, to determine if the trichome-related DE genes found in the leaf were tissue-specific.

### Characterization of transgressive segregation in the F_1_ hybrids

To further dissect the phenomenon of transgressive phenotypes following hybridization, DE transcripts identified from the leaf and petal master transcriptomes that were found to be significantly up- and down-regulated in the hybrid in comparison to both parental species were isolated. These transcripts were then queried against the *A. thaliana* TAIR10 proteins (ver. 20101214) using BLASTx [e-value ≤ 1e-3], and significant matches (best-hit only, bit score ≥ 50, % identity ≥ 30) were then cross-referenced to the TAIR10 functional description list (ver. 20170331) and recorded.

### Identification of positively selected genes between *M. candidum* and *M. sanguineum*

To identify genes under positive selection between *M. candidum* and *M. sanguineum*, orthologous transcripts were first identified from leaf and petal taxon-specific transcriptomes of both species using the bidirectional-best-hits method. A custom script incorporating MAFFT (for sequence alignment; Katoh and Standley 2013), PAL2NAL (for conversion of protein sequence alignment into codon alignments; Suyama *et al.* 2006) and KaKs_Calculator ver. 2.0 (for calculation of Ka and Ks values; Wang *et al.* 2010) was then used to estimate the non-synonymous (Ka) and synonymous (Ks) substitution rates between transcript pairs. Pairs with Ka/Ks ratios >1 and *P* ≤ 0.05 (Fisher’s exact test) were considered as positively selected, and were queried against the *A. thaliana* TAIR10 proteins (ver. 20101214; www.arabidopsis.org/, accessed 4 June 2018) using BLASTx [e-value ≤ 1e-3]. Significant matches (best-hit only, bit score ≥ 50, % identity ≥ 30) were then cross-referenced to the TAIR10 functional description list (ver. 20170331; www.arabidopsis.org/, accessed 4 June 2018) and recorded.

## Results

### *De novo* assembly of transcriptomes from RNA-seq data

Of the 50.0–62.7 million raw read pairs generated from Illumina sequencing of the cDNA libraries of *M. candidum*, *M. sanguineum* and the hybrid, 46.4–59.1 million read pairs survived quality filtering **[see ****Supporting Information—Table S1****]**. Trinity normalization retained 5.6–7.3 % and 8.2–13.1 % HQ reads at a maximum coverage of 50 for the assembly of the master and taxon-specific transcriptomes, respectively. Trinity generated 209 516 and 196 293 transcripts for the leaf and petal master transcriptomes, respectively, which were subsequently reduced to 50 519 and 48 120 by EvidentialGene. The EvidentialGene-reduced transcriptomes were regarded as the optimal, and hence as references for subsequent DE analyses. Together with the outcome of other assessments, the high mapping rates (>80 %) of HQ reads to the transcriptomes assembled in this study signified that they were of good quality. Details on the characterization and various assessments of the master and taxon-specific transcriptomes are listed in Table 1 and **Supporting Information—Table S2**, respectively.

**Table 1.** Statistics based on the master transcriptomes. Mc = *M. candidum*; Ms = *M. sanguineum*; Hy = F_1_ hybrid; rep = replicate. ^a^Based on EvidentialGene ‘okay’ transcripts. ^b^Filtered reads to EvidentialGene ‘okay’ transcripts.

	Leaf	Flower petal
No. of read pairs after normalization (% of total)	15 389 729 (7.3 %)	11 362 570 (5.6 %)
No. of contigs (Trinity output)	209 516	196 293
No. of contigs (EvidentialGene output, ‘okay’ transcripts)	50 519	48 120
Smallest contig^a^ (bp)	201	201
Largest contig^a^ (bp)	16 762	16 810
Total length^a^ (bp)	65 471 851	63 130 782
Mean length^a^ (bp)	1295.99	1311.95
% GC^a^	48.1	48.1
N50^a^ (bp)	1864	1885
Mapping rate^b^ (%)		
Mc (rep 1; rep 2)	88.3; 88.4	88.8; 89.5
Ms (rep 1; rep 2)	85.3; 84.6	89.7; 90.3
Hy (rep 1; rep 2)	88.4; 80.9	87.5; 89.6
BUSCO assessment^a^		
Complete (%)	92.8	92.6
Single-copy (%)	75.1	76.8
Duplicated (%)	17.7	15.8
Fragmented (%)	2.8	2.9
Missing (%)	4.4	4.5
Transcript length assessment^a^		
No. of transcripts >90 %	5404	5224
No. of transcripts >70 %	7558	7372
No. of DE transcripts (logFC ≥ 2, FDR ≤ 0.001)	3973	2116
KOBAS annotation (Mc-Ms-Hy)		
No. of transcripts annotated (% of total)	34 640 (68.6 %)	33579 (69.8 %)
No. of DE transcripts annotated (% of total)	3165 (79.7 %)	1545 (73.0 %)
No. of KEGG terms (corrected *P* ≤ 0.05)	2	3
No. of GO terms (corrected *P* ≤ 0.05)	92	61

GC=the nucleotides Guanine and Cytosine.

### Overall differential gene expression

**Figure 2.**
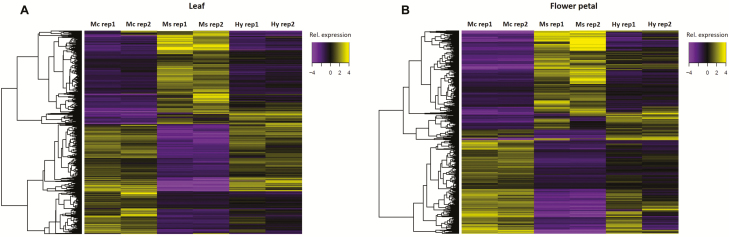
Heatmaps of relative expression levels of DE transcripts in the (A) leaf and (B) flower petal. (Mc = *M. candidum*; Ms = *M. sanguineum*; Hy = F_1_ hybrid; rep = replicate).

**Figure 3.**
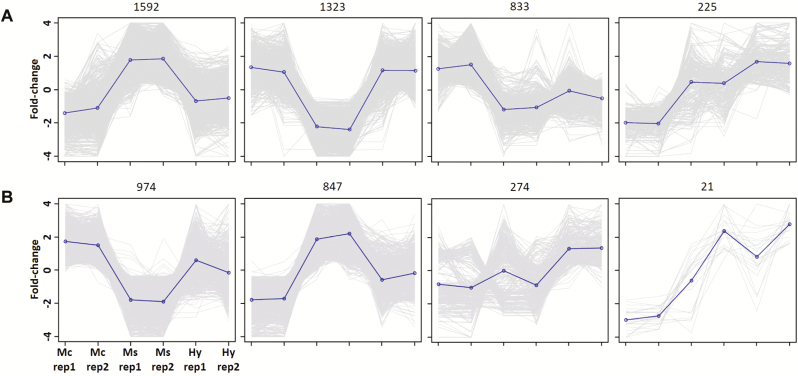
Clustering of transcripts with similar expression profiles, at 60 % height of the dendrograms of heatmaps in Fig. 2, for the (A) leaf and (B) flower petal. The number above each graph shows the number of transcripts that fall under the same cluster (Mc = *M. candidum*; Ms = *M. sanguineum*; Hy = F_1_ hybrid; rep = replicate).

To examine gene expressional differences, data from two biological replicates of each of *M. candidum*, *M. sanguineum* and the hybrid were mapped to the master transcriptomes for abundance estimation. Satisfying logFC ≥ 2 and FDR ≤ 0.001, EdgeR estimated a total of 3973 and 2116 DE transcripts in three-way comparisons (*M. candidum*–*M. sanguineum*–hybrid) for the leaf and petal transcriptomes, respectively (Table 1). In both tissue types, differential expression was highest between the parental species (*M. candidum*–*M. sanguineum*). The hybrid had less DE transcripts with *M. candidum* (hybrid–*M. candidum*) compared to with *M. sanguineum* (hybrid–*M. sanguineum*) in the leaf, and had similar numbers of DE transcripts with both parental species (hybrid–*M. candidum* and hybrid–*M. sanguineum*) in the petal (Table 2). Heatmaps that summarize the relative expression levels at all DE transcripts in the leaf and petal are shown in Fig. 2. Clustering the transcripts with similar patterns of expression across the samples resulted in four clusters each for the leaf and petal (Fig. 3). As seen from Figs 2 and 3, the expression levels of the hybrid at the majority of DE transcripts were similar to either *M. candidum* or *M. sanguineum*, rather than intermediate.

**Table 2.** Number of differentially expressed transcripts found in the leaf and flower petal master transcriptomes. Lower diagonal = leaf master transcriptome; upper diagonal = flower petal master transcriptome. Based on logFC ≥ 2, FDR ≤ 0.001.

	*M. candidum*	*M. sanguineum*	F_1_ hybrid
*M. candidum*	–	1886	418
*M. sanguineum*	3243	–	407
F_1_ hybrid	572	2025	–

### Functional enrichment analysis of DE transcripts

Using KOBAS, enrichment of KEGG and GO terms was conducted to explore the biological functions of the DE transcripts. In the three-way comparisons (*M. candidum*–*M. sanguineum*–hybrid), 79.7 % (3165 of 3973) and 73.0 % (1545 of 2116) of the leaf and petal DE transcripts were functionally annotated. Significant correlations were found for two and three KEGG terms, and 92 and 61 GO terms, for the leaf and petal, respectively (Table 1; **see ****Supporting Information—Table S3**). In the leaf, the enriched KEGG terms were ‘biosynthesis of secondary metabolites’ and ‘stilbenoid, diarylheptanoid and gingerol biosynthesis’; in the petal they were ‘biosynthesis of secondary metabolites’, ‘phenylpropanoid biosynthesis’ and ‘stilbenoid, diarylheptanoid and gingerol biosynthesis’. For the leaf, most GO terms fell under ‘biological process’ (63), followed by ‘cellular component’ (20) and ‘molecular function’ (9); for the petal, most GO terms fell under ‘biological process’ (41), followed by ‘molecular function’ (13) and ‘cellular component’ (7).

Between *M. candidum* and *M. sanguineum*, 78.9 % (2560 of 3243) and 74.1 % (1398 of 1886) of the leaf and petal DE transcripts were annotated, respectively. In the leaf, up-regulated transcripts in *M. candidum* were significantly related to two KEGG terms (‘biosynthesis of secondary metabolites’ and ‘flavonoid biosynthesis’) and 79 GO terms, while up-regulated transcripts in *M. sanguineum* were related to two KEGG terms (‘ribosome’ and ‘thiamine metabolism’) and 55 GO terms. In the petal, up-regulated transcripts in *M. candidum* were not significantly related to any KEGG or GO terms, while up-regulated transcripts in *M. sanguineum* were related to six KEGG terms (‘biosynthesis of secondary metabolites’, ‘phenylpropanoid biosynthesis’, ‘stilbenoid, diarylheptanoid and gingerol biosynthesis’, ‘flavonoid biosynthesis’, ‘phenylalanine metabolism’ and ‘limonene and pinene degradation’) and 82 GO terms **[see ****Supporting Information—Table S4****]**.

### Genes related to trichome development

Trichomes on the leaf surface are thought to be a morphological characteristic that plays a role in differential adaptation between *M. candidum* and *M. sanguineum*. Searching through the list of descriptions for *A. thaliana* functional genes, 122 genes were found to be related to trichome development. BLASTx of leaf DE transcripts between *M. candidum* and *M. sanguineum* revealed 159 transcripts significantly matching to 44 trichome-related *A. thaliana* genes **[see ****Supporting Information—Table S5a****]**, while 108 petal DE transcripts between *M. candidum* and *M. sanguineum* significantly matched 31 trichome-related *A. thaliana* genes **[see ****Supporting Information—Table S5b****]**. Twenty and seven of these trichome-related genes were tissue-specific in the leaf and petal, respectively.

### Transgressive segregation in hybrids

Transcripts that were significantly up- or down-regulated in the leaf and petal of the hybrid compared to *M. candidum* and *M. sanguineum* were extracted and annotated. In the leaf, 11 and 20 transcripts were found to be significantly up- and down-regulated, respectively, while in the petal, only one transcript was found to be significantly up-regulated but no transcripts were found to be significantly down-regulated, in the hybrid. BLASTx correlated seven and 14 transgressively segregated transcripts in the leaf of the hybrid to the same numbers of genes **[see ****Supporting Information—Table S6****]** in the list of *A. thaliana* functional genes. No significant match was found for the up-regulated transcript in the petal.

### Genes under positive selection

To examine genes possibly under positive selection between *M. candidum* and *M. sanguineum*, taxon-specific transcriptomes assembled for both species **[see ****Supporting Information—Table S2****]** were subjected to reciprocal BLAST, retaining only best hits that occurred in both directions for downstream Ka and Ks estimation. In total, 23 602 and 22 338 transcript pairs (*M. candidum*–*M. sanguineum*) were retained for the leaf and petal, respectively. Filtering for transcript pairs with Ka/Ks ratio >1 and *P* ≤ 0.05, 32 and 31 transcripts were obtained for the leaf and petal, respectively. BLASTx correlated 24 and 26 transcripts from the leaf and petal to the same numbers of genes **[see ****Supporting Information—Table S7****]** in the list of *A. thaliana* functional genes.

## Discussion

Having diversified through adaptive radiation (during which new species with very close genetic relationships are formed in a short period of time), frequent hybridization between many *Melastoma* species in nature has been observed, and some recognized ‘species’ have even been shown to be of hybrid origin (Dai *et al.* 2012; Liu *et al.* 2014; Zhou *et al.* 2017; Zou *et al.* 2017), highlighting the importance of hybridization in the evolution of *Melastoma*. Using two commonly found species in South China, *M. candidum* and *M. sanguineum*, as well as their F_1_ hybrid, this study aimed to characterize gene expressional patterns following hybridization. We also looked at the possible role of gene regulation in the differentiation between the two parental species, as well as between the parental species and their hybrid. Such insights may provide glimpses into possible mechanisms of diversification within *Melastoma*, especially since key morphological traits likely linked to their adaptive divergence are few, and reproductive isolation between many member species appears to be weak, illustrated by the many known cases of interspecific hybridization. In addition, we also identified positively selected genes to explore the role of natural selection in the evolution of *Melastoma* species.

### Assembly of master transcriptomes for cross-species comparison

Most studies on comparative transcriptomics of plants to date compared patterns of gene expression across treatments (e.g. Carvajal *et al.* 2018) or across ecotypes (e.g. Shen *et al.* 2012; Bell *et al.* 2013; He *et al.* 2013) of a single species. Examples of studies that conducted cross-species comparisons in plants using high-throughput sequencing data are relatively rare, and these either used the transcriptome/genome of one of the species in the comparison (e.g. Zhang *et al.* 2013) or of a closely related species (e.g. Guo *et al.* 2015), combined the genomes of the species in the comparison (e.g. Zhu *et al.* 2017), or isolated only the orthologous transcripts (e.g. Davidson *et al.* 2012; Roberts and Roalson 2017), as reference. While an unpublished draft genome of *M. candidum* (one of the target parental species) was available during this study, using it as a reference for mapping and estimation of expression abundance in *M. candidum*, *M. sanguineum* and the hybrid would introduce bias when these estimates are directly compared. Therefore, we explored the method of a ‘master’ reference transcriptome assembled using RNA-seq data from the two parental species to allow for cross-taxa comparison of gene expression. This was possible given the low sequence divergence between *M. candidum* and *M. sanguineum* (<2 %, data not shown; high cross-species mapping rates of >85 %, **see ****Supporting Information—Table S2**). Similar methods of using combined data from more than one closely related taxa for *de novo* assembly have been employed in comparative studies of birds (Wolf *et al.* 2010) and fish (Goetz *et al.* 2010). In this study, the draft genome of *M. candidum* was merely used as a substrate for grouping overlapping reads into clusters that will then be separately fed into Trinity for *de novo* transcriptome assembly (i.e. genome-guided assembly; Haas *et al.* 2013). Using this method, fewer, but more contiguous transcripts were assembled than without the reference genome (data not shown), generally suggesting a better assembly.

### Differential adaptation of *M. candidum* and *M. sanguineum*

In the functional analysis of DE transcripts between the two parental species, up-regulated transcripts in the leaf of *M. candidum* were enriched with functions associated with light-related responses (i.e. ‘response to light stimulus’, ‘response to radiation’, ‘response to light intensity’, ‘response to high light intensity’, ‘phototropism’ and ‘photoprotection’). This may explain the better adaptation of *M. candidum* to high sunlight intensity compared to *M. sanguineum*, reflected in their habitat preferences in the wild, whereby the former prefers habitats exposed to strong sunlight while the latter prefers shady environments. We note, however, that up-regulated genes do not necessarily confer better performance or better adaptation, and the true implications could only be established through future functional studies. Interestingly, response to karrikin, a group of plant growth regulators found in the smoke of burning plant material (Flematti *et al.* 2015), was found to also be up-regulated in the leaf of *M. candidum*. While usually associated with plants that grow immediately after bushfires or wildfires, sensitivity to karrikin has been associated with enhanced light responses in *A. thaliana* seedlings (Nelson *et al.* 2010; Flematti *et al.* 2015), possibly contributing to better response to sunlight in *M. candidum*. On the other hand, in *M. sanguineum*, up-regulated transcripts were enriched with functions related to the chloroplast, likely a characteristic of plants adapted to low-light conditions for more efficient light uptake (Leong and Anderson 1984).

The analysis on positively selected genes between *M. candidum* and *M. sanguineum* also identified some interesting genes related to the differential adaptation between *M. candidum* and *M. sanguineum*. MYB transcription factors are well-known regulators of phenylpropanoid metabolism in plants, which play roles in growth and development as well as in defence against biotic and abiotic stresses (Liu *et al.* 2015). The positively selected MYB20 and MYB55 have been associated with adaptive response to drought stress in *Arabidopsis* (Gao *et al.* 2014) and tolerance to high temperatures in rice (El-kereamy *et al.* 2012), respectively. The correlation between these genes and habitat preferences of both species are apparent—in the wild, *M. candidum* is found mostly on level ground while *M. sanguineum* prefers to grow on slopes (W. L. Ng and R. Zhou, pers. obs.); and when along an elevation, *M. candidum* grows at lower altitudes (<120 m) compared to *M. sanguineum* (<400 m; Chen 1983). In addition, several genes associated with the regulation of auxin were found to be under positive selection. Auxin affects plant growth and orientation in response to, among others, sunlight (Weijers *et al.* 2018), and this again seems to correlate with the adaptation of *M. candidum* and *M. sanguineum* to different sunlight conditions.

In this study, transcriptomic data from the petal can be considered as a control when making comparisons between *M. candidum* and *M. sanguineum*, since morphological differences observed in their flowers seem to confer little adaptive advantage. In fact, none of the DE transcripts found in the petals of *M. candidum* and *M. sanguineum* suggested any direct association with differential adaptation between *M. candidum* and *M. sanguineum*, such as responses to light, drought or temperature—the main factors that, we believe, contribute to differential adaptation between the two species. Noteworthy, however, is the up-regulation of transcripts related to cellular components found in the petal of *M. sanguineum*, which could explain the larger flowers of *M. sanguineum* compared to *M. candidum*.

### Trichome development in *M. candidum* and *M. sanguineum*

One of the main morphological differences in the leaves of the two species is in the presence (*M. candidum*) and absence (*M. sanguineum*) of trichomes on the surface of their mature leaves. Usually as defences against biotic attacks and abiotic stressors, trichomes are thought to play a major role in the protection from sunlight by increasing reflectance and reducing the heat load (Hauser 2014). This could be true given our observations in the field, as well as in our greenhouse experiments, in which the leaves of *M. candidum* tend to turn yellow and wilt when placed in the shade, while the leaves of *M. sanguineum* tend to turn red and appear scotched when placed under constant sunlight. We cannot, however, discount the importance of other adaptive roles that trichomes may play in *M. candidum* and *M. sanguineum*, such as protection against herbivory (Hauser 2014), given that in the wild the former (with longer trichomes) often occurs at open areas while the latter (with little/no trichomes) prefers shaded environments.

**Figure 4.**
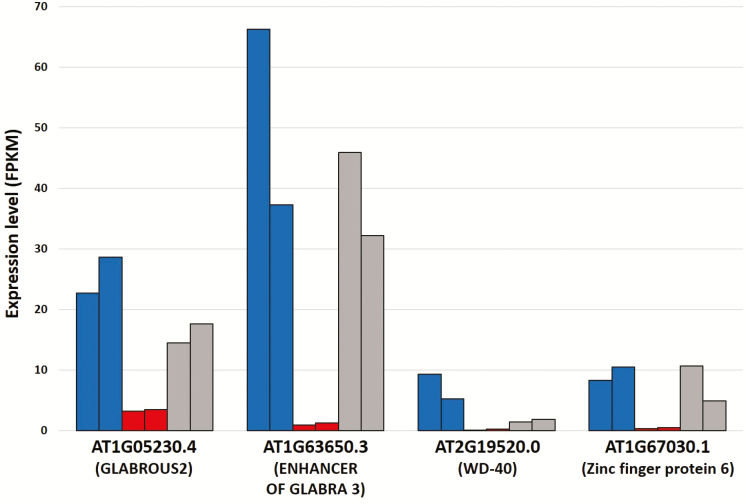
Expression levels of several genes related to trichome development in *M. candidum* (blue), *M. sanguineum* (red), and the F_1_ hybrid (grey).

Nonetheless, it was expected that many genes with functions related to trichome development are differentially expressed between *M. candidum* and *M. sanguineum*. These included members of a transcriptional network known to positively regulate trichome development, i.e. the MYB, basic helix-loop-helix (bHLH) and WD-40 repeat (WDR) proteins. In *Arabidopsis*, these proteins come together to form a trimeric complex that activates the expression of GLABROUS2, which induces trichome formation (Zhao *et al.* 2008). GLABROUS2 was also found in this study to be up-regulated in *M. candidum*. The involvement of several other up-regulated gene products in *M. candidum* in the positive regulation of trichome development, e.g. the C2H2 zinc finger protein, zinc finger protein 6 (ZFP6) and the ENHANCER OF GLABRA 3 (EGL3), is also well documented (Schellmann and Hulskamp 2005; Zhou *et al.* 2013; Pattanaik *et al.* 2014; Yan *et al.* 2014). Interestingly, the expression levels of these transcripts in the hybrid were mostly in between those of *M. candidum* and *M. sanguineum* (Fig. 4), likely a cause for the intermediate trichome length observed in the hybrid (Fig. 1).

A considerable number of proteins with trichome-related functions were also up-regulated in the glabrous leaves of *M. sanguineum*; *M. sanguineum* plantlets initially produce leaves with trichomes (1–2 mm in length) sparsely distributed on the upper surface, but lose them as the plants mature. Under the microscope, trichome ‘roots’ can still be observed below the epidermis of mature *M. sanguineum* leaves (Fig. 1), and the density of these structures remains almost constant throughout the growth phase of the leaf (R. Zhou, unpubl. data). These point to the possibility that trichomes actually continue to differentiate and form throughout a leaf’s development in *M. sanguineum*, although the part above the epidermis does not form, or forms and quickly aborts, leaving only the ‘root’ and a small bump on the surface intact. The detection of gene products in this study known to be expressed in trichomes proved that the trichomes (or part of) in mature *M. sanguineum* leaves are still intact and viable. One of the genes found up-regulated in *M. sanguineum* is GLABRA2, which positively regulates trichome initiation. However, ubiquitous expression of this gene was found to negatively affect subsequent trichome formation and development in *Arabidopsis*, resulting in glabrous leaves with aborted trichomes that appear stubby (Rerie *et al.* 1994; Srinivas 2004), like that observed in *M. sanguineum*. This resemblance hints at the possible role of GLABRA2 in causing the ‘absence’ of trichomes on the leaves of *M. sanguineum*.

### Patterns of gene expression in the F_1_ hybrid

The clustering of DE transcripts based on expression levels among *M. candidum*, *M. sanguineum* and the hybrid provided a general idea of the patterns of expression across them. Almost all possible modes of expression were observed in the hybrid—expression levels similar to either *M. candidum* or *M. sanguineum* (i.e. parental expression dominance), expression levels intermediate between *M. candidum* and *M. sanguineum* (i.e. additive expression), as well as expression levels exceeding both *M. candidum* and *M. sanguineum* (i.e. transgressive expression). Among the DE transcripts found in this study, parental level gene expression dominance was the most common in the hybrid, demonstrating that the mode of expression in plants following hybridization may not always be mostly additive (cf. Swanson-Wagner *et al.* 2006; Wu *et al.* 2016; Taliercio *et al.* 2017). In fact, various mechanisms, such as genomic imprinting (Wolf *et al.* 2014) and regulatory divergence between parental alleles (Combes *et al.* 2015), are known to alter gene expression in the hybrids in relation to the parents.

### Transgressive segregation following hybridization

The hybrid between *M. candidum* and *M. sanguineum* is widespread in South China, to a point where it was once even recognized as a species (Chen 1983) until a recent molecular study (Liu *et al.* 2014) confirmed its hybrid status. Aside from intermediate trichome structures on the leaf, branch and hypanthium, the hybrid displays obvious vigour in several other morphological characteristics—faster growth, as well as larger leaf and flower (diameter of ~6 cm in *M. candidum*, ~7.5 cm in *M. sanguineum* and ~9 cm in the hybrid)—in comparison to both of its parents (W. L. Ng and R. Zhou, pers. obs.). The functional information of the transgressively expressed transcripts in the hybrid suggested some molecular mechanisms behind its vigour, as several up-regulated gene products are thought to be associated with enhanced growth in plants. For example, the overexpression of SAUR proteins has been shown to promote plant growth in *Arabidopsis* (Li *et al.* 2015) possibly through inducing cell elongation and growth (van Mourik *et al.* 2017), and the overexpression of tonoplastic intrinsic protein has been associated with cell enlargement and accelerated cell division in *Arabidopsis* and tobacco (Ludevid *et al.* 1992; Okubo-Kurihara *et al.* 2008).

Aside from morphological changes, the hybrid between *M. candidum* and *M. sanguineum* has also been observed to be able to grow under various conditions, even those not preferred by either parent, for example across sunlight intensities, terrains and temperatures (W. L. Ng and R. Zhou, pers. obs.). In the hybrid, we found an up-regulated gene product associated with adaptation to stress in new environmental conditions—the plant ABC transporter, which helps infer tolerance to many kinds of stresses by transporting chemicals critical for adaptation to terrestrial habitats (Hwang *et al.* 2016). To afford such a regulation of adaptability would be an advantageous and a critical property that could have led to the rapid adaptive speciation within *Melastoma* in only the past 1 million years (Renner and Meyer 2001), and transgressive segregation following hybridization could have been the force that initially drove the generation of this diversity (Kagawa and Takimoto 2018).

## Data

Illumina sequencing data from this study have been deposited in the NCBI SRA database (accession number PRJNA526807). Transcriptomes assembled in this study and the custom scripts used for analysis in this study have been deposited in Figshare (available via the link https://doi.org/10.6084/m9.figshare.7824845.v1).

## Sources of Funding

This study was supported by grants from the National Natural Science Foundation of China (31670210 and 31811530297), Guangdong Natural Science Foundation (2015A030302011), Science and Technology Program of Guangzhou (201707010090) and Chang Hungta Science Foundation of Sun Yat-sen University.

## Contributions by the Authors

W.L.N. and R.Z. conceived and designed the study, W.L.N. and P.Z. performed the experiments, W.L.N. and W.W. performed the analyses, W.L.N. wrote the manuscript. All authors revised the manuscript.

## Conflict of Interest

None declared.

## Supporting Information

The following additional information is available in the online version of this article—

**Table S1.** Sequence data used in this study.

**Table S2.** Statistics based on the taxon-specific transcriptomes.

**Table S3.** Functional annotation of differentially expressed (DE) transcripts among *Melastoma candidum*, *M. sanguineum* and their F_1_ hybrid.

**Table S4.** Functional annotation of differentially expressed (DE) transcripts between *Melastoma candidum* and *M. sanguineum.*

**Table S5.** Annotation of trichome-related differentially expressed (DE) transcripts between *Melastoma sanguineum* and *M. candidum.*

**Table S6.** Annotation of transgressively segregated transcripts in the F_1_ hybrid leaf tissue.

**Table S7.** Annotation of putative genes under positive selection between *Melastoma candidum* and *M. sanguineum.*

Supplementary Tables
