# Supplementary material for: Comparative transcriptomics sheds light on differential adaptation and species diversification between two Melastoma species and their F1 hybrid
Source: AoB Plants. 2019 Mar 28;11(2):plz019. doi: 10.1093/aobpla/plz019 (PMC6481908; doi:10.1093/aobpla/plz019)
Supplement: Supplementary Tables [file plz019_suppl_supplementary_tables.pdf]

**Table S1** Sequence data used in this study.

| | Mc_leaf | Ms_leaf | Hy_leaf | Mc_petal | Ms_petal | Hy_petal |
|---|---|---|---|---|---|---|
| No. of raw read pairs: | | | | | | |
| Replicate 1 | 55,858,276 | 57,934,550 | 50,038,077 | 53,026,585 | 62,716,934 | 56,347,045 |
| Replicate 2 | 61,121,860 | 51,048,834 | 52,896,731 | 50,326,530 | 51,762,064 | 59,317,692 |
| No. of filtered read pairs: | | | | | | |
| Replicate 1 | 51,842,017 | 54,439,138 | 46,360,251 | 49,037,317 | 59,111,340 | 53,078,325 |
| Replicate 2 | 56,641,557 | 48,120,727 | 49,981,094 | 46,650,474 | 48,699,002 | 56,153,126 |

ABBREVIATIONS: Ms=*M. candidum* ; Ms=*M. sanguineum* ; Hy=F1 hybrid.

**Table S2** Statistics based on the taxon-specific transcriptomes.

| | Mc_leaf | Ms_leaf | Hy_leaf | Mc_petal | Ms_petal | Hy_petal |
|---|---|---|---|---|---|---|
| No. read pairs after normalization | 10910170 | 11385909 | 12590897 | 8719081 | 8783127 | 9398992 |
| % read pairs after normalization | 10.06 | 11.1 | 13.07 | 9.11 | 8.15 | 8.6 |
| No. contigs (Trinity output) | 157965 | 162300 | 121882 | 155546 | 144152 | 164465 |
| No. of contigs (EvidentialGene output, 'okay' transcripts) | 43344 | 42342 | 39929 | 39665 | 40738 | 42493 |
| Smallest contig (bp) | 201 | 201 | 201 | 201 | 201 | 201 |
| Largest contig (bp) | 16315 | 16742 | 15566 | 15576 | 16810 | 15952 |
| Total length (bp) | 59064274 | 55882078 | 50462163 | 54750598 | 54757615 | 56037672 |
| Mean length (bp) | 1362.69 | 1319.78 | 1263.8 | 1380.33 | 1344.14 | 1318.75 |
| % GC | 48.3 | 48.3 | 48.7 | 48.2 | 48.4 | 48.3 |
| N50 (bp) | 1890 | 1868 | 1766 | 1909 | 1889 | 1857 |
| Within-species mapping rate (%): | | | | | | |
| Replicate 1 | 88.9 | 88.6 | 90.6 | 91.3 | 91.2 | 88.6 |
| Replicate 2 | 88.9 | 87.3 | 83.7 | 92 | 92.3 | 90.7 |
| Cross-species mapping rate (%): | | | | | | |
| Replicate 1 | 86.9 | 88.7 | - | 90.4 | 89.4 | - |
| Replicate 2 | 85.5 | 88.5 | - | 89.9 | 90 | - |
| BUSCO assessment: | | | | | | |
| Complete (%) | 94.1 | 93 | 92.9 | 90.5 | 92.6 | 90.4 |
| Single-copy (%) | 75.7 | 77.1 | 76.9 | 75.2 | 77.1 | 77 |
| Duplicated (%) | 18.4 | 15.9 | 16 | 15.3 | 15.5 | 13.4 |
| Fragmented (%) | 1.8 | 2.8 | 2.4 | 3.5 | 2.4 | 3.8 |
| Missing (%) | 4.1 | 4.2 | 4.7 | 6 | 5 | 5.8 |
| Transcript length assessment (UniProtKB/Swiss-Prot): | | | | | | |
| No. transcripts >90% | 5391 | 5301 | 5111 | 5051 | 5336 | 5102 |
| No. transcripts >70% | 7446 | 7363 | 7131 | 7034 | 7349 | 7122 |

ABBREVIATIONS: Mc=*M. candidum*; Ms=*M. sanguineum*; Hy=F1 hybrid.

NOTES: Transcriptome statistics are based on EvidentialGene 'okay' transcripts. Mapping rates are based on mapping of quality-filtered reads to EvidentialGene 'okay' transcripts.

**Table S3** Functional annotation of DE transcripts among *M. candidum*, *M. sanguineum*, and their F1 hybrid.

(a) Leaf

| Term | Database | ID | No. of input | No. of background | Benjamini and Hochberg corrected *p*-value | GO category |
|---|---|---|---|---|---|---|
| Biosynthesis of secondary metabolites | KEGG | ath01110 | 216 | 1777 | 5.50E-03 | - |
| Stilbenoid, diarylheptanoid and gingerol biosynthesis | KEGG | ath00945 | 16 | 60 | 2.90E-02 | - |
| Metabolic process | GO | GO:0008152 | 1503 | 15444 | 4.50E-02 | |
| Single-organism process | GO | GO:0044699 | 1331 | 13293 | 3.40E-03 | |
| Response to stimulus | GO | GO:0050896 | 978 | 9099 | 7.10E-06 | |
| Single-organism metabolic process | GO | GO:0044710 | 662 | 5527 | 2.10E-09 | |
| Response to stress | GO | GO:0006950 | 564 | 5100 | 6.40E-04 | |
| Response to chemical | GO | GO:0042221 | 509 | 4427 | 4.90E-05 | |
| Response to abiotic stimulus | GO | GO:0009628 | 408 | 3150 | 1.30E-08 | |
| Response to organic substance | GO | GO:0010033 | 349 | 3194 | 3.30E-02 | |
| Single-organism biosynthetic process | GO | GO:0044711 | 327 | 2538 | 2.00E-06 | |
| Response to oxygen-containing compound | GO | GO:1901700 | 306 | 2510 | 2.50E-04 | |
| Small molecule metabolic process | GO | GO:0044281 | 274 | 2422 | 2.50E-02 | |
| Oxidation-reduction process | GO | GO:0055114 | 272 | 2165 | 1.30E-04 | |
| Response to acid chemical | GO | GO:0001101 | 243 | 1963 | 9.80E-04 | |
| Organic acid metabolic process | GO | GO:0006082 | 197 | 1649 | 1.80E-02 | |
| Response to inorganic substance | GO | GO:0010035 | 190 | 1486 | 1.60E-03 | |
| Oxoacid metabolic process | GO | GO:0043436 | 184 | 1521 | 1.70E-02 | |
| Carboxylic acid metabolic process | GO | GO:0019752 | 174 | 1477 | 4.60E-02 | |
| Response to light stimulus | GO | GO:0009416 | 156 | 1169 | 1.50E-03 | |
| Response to radiation | GO | GO:0009314 | 156 | 1204 | 4.30E-03 | |
| Response to temperature stimulus | GO | GO:0009266 | 126 | 904 | 1.70E-03 | |
| Small molecule biosynthetic process | GO | GO:0044283 | 121 | 968 | 4.40E-02 | |
| Monocarboxylic acid metabolic process | GO | GO:0032787 | 116 | 898 | 2.50E-02 | |
| Secondary metabolic process | GO | GO:0019748 | 110 | 598 | 1.80E-07 | |
| Response to oxidative stress | GO | GO:0006979 | 96 | 644 | 1.80E-03 | |

| | | | | | | |
|---|---|---|---|---|---|---|
| Response to cold | GO | GO:0009409 | 87 | 632 | 2.30E-02 | Biological Process |
| Cellular carbohydrate metabolic process | GO | GO:0044262 | 86 | 614 | 1.80E-02 | |
| Response to water | GO | GO:0009415 | 82 | 520 | 1.50E-03 | |
| Response to water deprivation | GO | GO:0009414 | 82 | 519 | 1.40E-03 | |
| Secondary metabolite biosynthetic process | GO | GO:0044550 | 80 | 434 | 2.00E-05 | |
| Generation of precursor metabolites and energy | GO | GO:0006091 | 78 | 534 | 1.20E-02 | |
| Phenylpropanoid metabolic process | GO | GO:0009698 | 70 | 353 | 1.20E-05 | |
| Flavonoid metabolic process | GO | GO:0009812 | 65 | 312 | 8.60E-06 | |
| Response to jasmonic acid | GO | GO:0009753 | 65 | 436 | 2.10E-02 | |
| Cellular polysaccharide metabolic process | GO | GO:0044264 | 59 | 398 | 3.30E-02 | |
| Flavonoid biosynthetic process | GO | GO:0009813 | 59 | 282 | 2.60E-05 | |
| Photosynthesis | GO | GO:0015979 | 59 | 361 | 6.90E-03 | |
| Phenylpropanoid biosynthetic process | GO | GO:0009699 | 58 | 286 | 7.00E-05 | |
| Cell wall biogenesis | GO | GO:0042546 | 56 | 370 | 3.10E-02 | |
| Cellular carbohydrate biosynthetic process | GO | GO:0034637 | 49 | 319 | 4.40E-02 | |
| Pigment metabolic process | GO | GO:0042440 | 49 | 290 | 1.10E-02 | |
| Pigment biosynthetic process | GO | GO:0046148 | 42 | 236 | 1.20E-02 | |
| Cellular polysaccharide biosynthetic process | GO | GO:0033692 | 40 | 247 | 4.80E-02 | |
| Response to karrikin | GO | GO:0080167 | 40 | 227 | 1.80E-02 | |
| Rhythmic process | GO | GO:0048511 | 40 | 216 | 9.10E-03 | |
| Circadian rhythm | GO | GO:0007623 | 39 | 201 | 5.50E-03 | |
| Lignin metabolic process | GO | GO:0009808 | 35 | 204 | 4.40E-02 | |
| Plant-type secondary cell wall biogenesis | GO | GO:0009834 | 26 | 127 | 2.60E-02 | |
| Cellular response to oxidative stress | GO | GO:0034599 | 25 | 117 | 2.10E-02 | |
| Regulation of secondary metabolic process | GO | GO:0043455 | 20 | 83 | 2.20E-02 | |
| Sulfate assimilation | GO | GO:0000103 | 16 | 61 | 3.20E-02 | |
| Anthocyanin-containing compound biosynthetic process | GO | GO:0009718 | 14 | 50 | 3.80E-02 | |
| RNA secondary structure unwinding | GO | GO:0010501 | 14 | 52 | 4.70E-02 | |
| Abscisic acid metabolic process | GO | GO:0009687 | 13 | 38 | 1.80E-02 | |
| Chloroplast localization | GO | GO:0019750 | 12 | 40 | 4.70E-02 | |
| Lignan biosynthetic process | GO | GO:0009807 | 12 | 19 | 7.50E-04 | |
| Lignan metabolic process | GO | GO:0009806 | 12 | 19 | 7.50E-04 | |

| | | | | | | |
|---|---|---|---|---|---|---|
| Plastid localization | GO | GO:0051644 | 12 | 40 | 4.70E-02 | |
| Abscisic acid biosynthetic process | GO | GO:0009688 | 11 | 28 | 1.90E-02 | |
| Chloroplast relocation | GO | GO:0009902 | 11 | 34 | 4.70E-02 | |
| Establishment of plastid localization | GO | GO:0051667 | 11 | 34 | 4.70E-02 | |
| Oxalate metabolic process | GO | GO:0033609 | 8 | 16 | 3.10E-02 | |
| Regulation of abscisic acid biosynthetic process | GO | GO:0010115 | 8 | 15 | 2.50E-02 | |
| Response to herbicide | GO | GO:0009635 | 6 | 8 | 3.20E-02 | |
| Oxidoreductase activity | GO | GO:0016491 | 252 | 2048 | 9.80E-04 | Molecular Function |
| Oxidoreductase activity, acting on paired donors, with incorporation or reduction of molecular oxygen | GO | GO:0016705 | 66 | 457 | 3.20E-02 | |
| Iron ion binding | GO | GO:0005506 | 59 | 390 | 2.50E-02 | |
| Monooxygenase activity | GO | GO:0004497 | 52 | 335 | 3.00E-02 | |
| Glucosyltransferase activity | GO | GO:0046527 | 40 | 231 | 2.20E-02 | |
| UDP-glucosyltransferase activity | GO | GO:0035251 | 40 | 217 | 9.70E-03 | |
| Carbon-carbon lyase activity | GO | GO:0016830 | 36 | 179 | 5.50E-03 | |
| Heat shock protein binding | GO | GO:0031072 | 14 | 50 | 3.80E-02 | |
| Flavonoid sulfotransferase activity | GO | GO:1990135 | 6 | 9 | 4.50E-02 | |
| Plastid | GO | GO:0009536 | 668 | 5582 | 2.10E-09 | Cellular Component |
| Chloroplast | GO | GO:0009507 | 656 | 5468 | 2.10E-09 | |
| Extracellular region | GO | GO:0005576 | 315 | 2699 | 2.30E-03 | |
| Plastid part | GO | GO:0044435 | 289 | 2188 | 2.00E-06 | |
| Chloroplast part | GO | GO:0044434 | 284 | 2160 | 3.50E-06 | |
| Plastid stroma | GO | GO:0009532 | 159 | 1187 | 1.10E-03 | |
| Chloroplast stroma | GO | GO:0009570 | 154 | 1159 | 1.80E-03 | |
| Cell wall | GO | GO:0005618 | 143 | 1130 | 1.60E-02 | |
| External encapsulating structure | GO | GO:0030312 | 143 | 1130 | 1.60E-02 | |
| Plastid envelope | GO | GO:0009526 | 139 | 1132 | 3.80E-02 | |
| Chloroplast envelope | GO | GO:0009941 | 138 | 1107 | 2.70E-02 | |
| Thylakoid | GO | GO:0009579 | 126 | 875 | 6.40E-04 | |
| Chloroplast thylakoid | GO | GO:0009534 | 106 | 738 | 2.30E-03 | |
| Plastid thylakoid | GO | GO:0031976 | 106 | 738 | 2.30E-03 | |
| Apoplast | GO | GO:0048046 | 100 | 743 | 2.00E-02 | |

| | | | | | | |
|---|---|---|---|---|---|---|
| Thylakoid part | GO | GO:0044436 | 93 | 652 | 7.70E-03 | |
| Photosynthetic membrane | GO | GO:0034357 | 87 | 602 | 8.20E-03 | |
| Thylakoid membrane | GO | GO:0042651 | 87 | 601 | 8.00E-03 | |
| Chloroplast thylakoid membrane | GO | GO:0009535 | 84 | 578 | 8.70E-03 | |
| Plastid thylakoid membrane | GO | GO:0055035 | 84 | 578 | 8.70E-03 | |

(b) Flower petal

| Term | Database | ID | No. of input | No. of background | Benjamini and Hochberg corrected *p*-value | GO category |
|---|---|---|---|---|---|---|
| Biosynthesis of secondary metabolites | KEGG | ath01110 | 109 | 1715 | 7.50E-04 | - |
| Phenylpropanoid biosynthesis | KEGG | ath00940 | 27 | 205 | 4.40E-06 | - |
| Stilbenoid, diarylheptanoid and gingerol biosynthesis | KEGG | ath00945 | 11 | 51 | 7.40E-05 | - |
| Single-organism process | GO | GO:0044699 | 667 | 12768 | 2.90E-05 | |
| Response to stimulus | GO | GO:0050896 | 533 | 8776 | 7.90E-13 | |
| Single-organism metabolic process | GO | GO:0044710 | 318 | 5306 | 7.10E-07 | |
| Response to stress | GO | GO:0006950 | 304 | 4894 | 6.20E-08 | |
| Response to chemical | GO | GO:0042221 | 271 | 4288 | 9.80E-08 | |
| Response to abiotic stimulus | GO | GO:0009628 | 192 | 3134 | 5.40E-05 | |
| Response to organic substance | GO | GO:0010033 | 187 | 3112 | 1.80E-04 | |
| Defense response | GO | GO:0006952 | 162 | 2074 | 2.30E-10 | |
| Response to endogenous stimulus | GO | GO:0009719 | 162 | 2726 | 7.90E-04 | |
| Single-organism biosynthetic process | GO | GO:0044711 | 162 | 2450 | 5.40E-06 | |
| Response to hormone | GO | GO:0009725 | 154 | 2574 | 8.20E-04 | |
| Oxidation-reduction process | GO | GO:0055114 | 147 | 2042 | 2.00E-07 | |
| Response to external stimulus | GO | GO:0009605 | 134 | 2045 | 5.40E-05 | |
| Response to biotic stimulus | GO | GO:0009607 | 113 | 1538 | 2.30E-06 | |
| Response to external biotic stimulus | GO | GO:0043207 | 100 | 1482 | 1.80E-04 | |
| Response to other organism | GO | GO:0051707 | 100 | 1482 | 1.80E-04 | |
| Response to radiation | GO | GO:0009314 | 84 | 1193 | 1.70E-04 | |
| Response to light stimulus | GO | GO:0009416 | 83 | 1154 | 9.80E-05 | |
| Secondary metabolic process | GO | GO:0019748 | 74 | 548 | 1.00E-14 | |

| | | | | | | |
|---|---|---|---|---|---|---|
| Defense response to other organism | GO | GO:0098542 | 72 | 1032 | 6.20E-04 | Biological Process |
| Secondary metabolite biosynthetic process | GO | GO:0044550 | 55 | 403 | 1.90E-11 | |
| Phenylpropanoid metabolic process | GO | GO:0009698 | 46 | 324 | 2.70E-10 | |
| Phenylpropanoid biosynthetic process | GO | GO:0009699 | 38 | 257 | 3.50E-09 | |
| Response to wounding | GO | GO:0009611 | 35 | 379 | 1.90E-04 | |
| Regulation of hormone levels | GO | GO:0010817 | 34 | 389 | 5.90E-04 | |
| Flavonoid biosynthetic process | GO | GO:0009813 | 33 | 262 | 9.90E-07 | |
| Flavonoid metabolic process | GO | GO:0009812 | 33 | 293 | 8.70E-06 | |
| Lignin metabolic process | GO | GO:0009808 | 30 | 189 | 3.70E-08 | |
| Response to karrikin | GO | GO:0080167 | 24 | 223 | 2.60E-04 | |
| Lignin biosynthetic process | GO | GO:0009809 | 23 | 159 | 5.60E-06 | |
| Hormone metabolic process | GO | GO:0042445 | 22 | 190 | 1.80E-04 | |
| Regulation of secondary metabolic process | GO | GO:0043455 | 15 | 75 | 8.50E-06 | |
| Cellular response to metal ion | GO | GO:0071248 | 12 | 74 | 3.80E-04 | |
| Cellular response to iron ion | GO | GO:0071281 | 11 | 49 | 5.40E-05 | |
| Response to iron ion | GO | GO:0010039 | 11 | 67 | 6.00E-04 | |
| Regulation of phenylpropanoid metabolic process | GO | GO:2000762 | 10 | 51 | 3.10E-04 | |
| Regulation of secondary metabolite biosynthetic process | GO | GO:1900376 | 10 | 42 | 7.70E-05 | |
| Flavone biosynthetic process | GO | GO:0051553 | 7 | 27 | 5.90E-04 | |
| Flavone metabolic process | GO | GO:0051552 | 7 | 27 | 5.90E-04 | |
| L-phenylalanine metabolic process | GO | GO:0006558 | 7 | 27 | 5.90E-04 | |
| Regulation of lignin biosynthetic process | GO | GO:1901141 | 7 | 23 | 2.60E-04 | |
| Oxidoreductase activity | GO | GO:0016491 | 144 | 1934 | 4.10E-08 | |
| Oxidoreductase activity, acting on paired donors, with incorporation or reduction of molecular oxygen | GO | GO:0016705 | 58 | 420 | 3.50E-12 | |
| Tetrapyrrole binding | GO | GO:0046906 | 54 | 430 | 4.50E-10 | |
| Heme binding | GO | GO:0020037 | 50 | 389 | 9.80E-10 | |
| Iron ion binding | GO | GO:0005506 | 49 | 359 | 2.40E-10 | |
| Monooxygenase activity | GO | GO:0004497 | 45 | 309 | 2.00E-10 | |
| Oxygen binding | GO | GO:0019825 | 37 | 226 | 4.70E-10 | |
| Dioxygenase activity | GO | GO:0051213 | 21 | 170 | 1.10E-04 | |

| | | | | | | |
|---|---|---|---|---|---|---|
| S-adenosylmethionine-dependent methyltransferase activity | GO | GO:0008757 | 21 | 200 | 8.10E-04 | Molecular Function |
| Oxidoreductase activity, acting on paired donors, with incorporation or reduction of molecular oxygen, NAD(P)H as one donor, and incorporation of one atom of oxygen | GO | GO:0016709 | 19 | 165 | 5.10E-04 | |
| Carboxylic acid binding | GO | GO:0031406 | 18 | 144 | 2.90E-04 | |
| Oxidoreductase activity, acting on paired donors, with incorporation or reduction of molecular oxygen, 2-oxoglutarate as one donor, and incorporation of one atom each of oxygen into both donors | GO | GO:0016706 | 16 | 96 | 3.20E-05 | |
| O-methyltransferase activity | GO | GO:0008171 | 11 | 48 | 4.60E-05 | |
| Intrinsic component of membrane | GO | GO:0031224 | 388 | 7125 | 1.90E-04 | Cellular Component |
| Integral component of membrane | GO | GO:0016021 | 373 | 6887 | 3.90E-04 | |
| Cell periphery | GO | GO:0071944 | 371 | 6749 | 1.40E-04 | |
| Extracellular region | GO | GO:0005576 | 181 | 2627 | 1.20E-07 | |
| Apoplast | GO | GO:0048046 | 55 | 736 | 6.20E-04 | |
| Intrinsic component of plasma membrane | GO | GO:0031226 | 48 | 590 | 2.40E-04 | |
| Integral component of plasma membrane | GO | GO:0005887 | 37 | 441 | 7.00E-04 | |

**Table S4** Functional annotation of DE transcripts between *M. candidum* and *M. sanguineum* .

(a) Leaf - Mc up-regulated

| Term | Database | ID | No. of input | No. of background | Benjamini and Hochberg corrected *p* -value | GO category |
|---|---|---|---|---|---|---|
| Biosynthesis of secondary metabolites | KEGG | ath01110 | 108 | 1777 | 3.06E-03 | - |
| Flavonoid biosynthesis | KEGG | ath00941 | 13 | 62 | 9.95E-04 | - |
| Single-organism process | GO | GO:0044699 | 610 | 13293 | 2.45E-03 | |
| Single-organism cellular process | GO | GO:0044763 | 446 | 9803 | 4.91E-02 | |
| Response to stimulus | GO | GO:0050896 | 438 | 9099 | 1.60E-03 | |
| Single-organism metabolic process | GO | GO:0044710 | 313 | 5527 | 3.94E-07 | |
| Response to chemical | GO | GO:0042221 | 233 | 4427 | 2.17E-03 | |
| Response to abiotic stimulus | GO | GO:0009628 | 208 | 3150 | 1.19E-08 | |
| Response to organic substance | GO | GO:0010033 | 172 | 3194 | 6.42E-03 | |
| Single-organism biosynthetic process | GO | GO:0044711 | 158 | 2538 | 3.80E-05 | |
| Response to endogenous stimulus | GO | GO:0009719 | 151 | 2773 | 1.00E-02 | |
| Response to oxygen-containing compound | GO | GO:1901700 | 149 | 2510 | 5.22E-04 | |
| Response to hormone | GO | GO:0009725 | 146 | 2615 | 5.34E-03 | |
| Response to acid chemical | GO | GO:0001101 | 124 | 1963 | 2.89E-04 | |
| Oxidation-reduction process | GO | GO:0055114 | 121 | 2165 | 1.61E-02 | |
| Organic acid metabolic process | GO | GO:0006082 | 93 | 1649 | 4.23E-02 | |
| Response to light stimulus | GO | GO:0009416 | 88 | 1169 | 3.30E-05 | |
| Response to radiation | GO | GO:0009314 | 88 | 1204 | 8.22E-05 | |
| Single-organism carbohydrate metabolic process | GO | GO:0044723 | 64 | 980 | 1.35E-02 | |
| Secondary metabolic process | GO | GO:0019748 | 51 | 598 | 3.61E-04 | |
| Cellular carbohydrate metabolic process | GO | GO:0044262 | 47 | 614 | 4.82E-03 | |
| Response to auxin | GO | GO:0009733 | 45 | 664 | 3.70E-02 | |
| Secondary metabolite biosynthetic process | GO | GO:0044550 | 43 | 434 | 9.35E-05 | |
| Flavonoid metabolic process | GO | GO:0009812 | 41 | 312 | 4.52E-07 | |
| Cell wall biogenesis | GO | GO:0042546 | 39 | 370 | 8.86E-05 | |
| Response to jasmonic acid | GO | GO:0009753 | 39 | 436 | 1.60E-03 | |
| Response to water | GO | GO:0009415 | 38 | 520 | 2.76E-02 | |

| | | | | | | |
|---|---|---|---|---|---|---|
| Response to water deprivation | GO | GO:0009414 | 38 | 519 | 2.71E-02 | Biological Process |
| Carbohydrate biosynthetic process | GO | GO:0016051 | 37 | 493 | 2.35E-02 | |
| Phenylpropanoid metabolic process | GO | GO:0009698 | 37 | 353 | 1.52E-04 | |
| Photosynthesis | GO | GO:0015979 | 36 | 361 | 4.94E-04 | |
| Flavonoid biosynthetic process | GO | GO:0009813 | 35 | 282 | 1.86E-05 | |
| Plant-type cell wall organization or biogenesis | GO | GO:0071669 | 35 | 397 | 3.81E-03 | |
| Anion transport | GO | GO:0006820 | 33 | 385 | 7.04E-03 | |
| Phenylpropanoid biosynthetic process | GO | GO:0009699 | 33 | 286 | 9.60E-05 | |
| Cellular polysaccharide metabolic process | GO | GO:0044264 | 32 | 398 | 1.97E-02 | |
| Response to karrikin | GO | GO:0080167 | 31 | 227 | 1.86E-05 | |
| Cellular carbohydrate biosynthetic process | GO | GO:0034637 | 30 | 319 | 4.45E-03 | |
| Plant-type cell wall biogenesis | GO | GO:0009832 | 30 | 276 | 6.35E-04 | |
| Pigment metabolic process | GO | GO:0042440 | 28 | 290 | 4.82E-03 | |
| Cellular polysaccharide biosynthetic process | GO | GO:0033692 | 26 | 247 | 2.81E-03 | |
| Polysaccharide biosynthetic process | GO | GO:0000271 | 26 | 301 | 2.37E-02 | |
| Response to heat | GO | GO:0009408 | 26 | 315 | 3.70E-02 | |
| Pigment biosynthetic process | GO | GO:0046148 | 24 | 236 | 6.42E-03 | |
| Response to light intensity | GO | GO:0009642 | 20 | 199 | 1.97E-02 | |
| Glucan biosynthetic process | GO | GO:0009250 | 18 | 162 | 1.48E-02 | |
| Plant-type secondary cell wall biogenesis | GO | GO:0009834 | 18 | 127 | 1.94E-03 | |
| Response to high light intensity | GO | GO:0009644 | 15 | 115 | 1.10E-02 | |
| Inorganic anion transport | GO | GO:0015698 | 14 | 116 | 2.57E-02 | |
| Regulation of flavonoid biosynthetic process | GO | GO:0009962 | 12 | 59 | 2.23E-03 | |
| Anthocyanin-containing compound biosynthetic process | GO | GO:0009718 | 11 | 50 | 2.45E-03 | |
| Anthocyanin-containing compound metabolic process | GO | GO:0046283 | 11 | 74 | 2.35E-02 | |
| Regulation of anthocyanin biosynthetic process | GO | GO:0031540 | 8 | 27 | 4.63E-03 | |
| Regulation of anthocyanin metabolic process | GO | GO:0031537 | 8 | 40 | 2.42E-02 | |
| Starch biosynthetic process | GO | GO:0019252 | 8 | 42 | 2.90E-02 | |
| Cellulose microfibril organization | GO | GO:0010215 | 7 | 36 | 4.91E-02 | |
| Phototropism | GO | GO:0009638 | 7 | 35 | 4.36E-02 | |
| Lignan biosynthetic process | GO | GO:0009807 | 6 | 19 | 1.83E-02 | |
| Lignan metabolic process | GO | GO:0009806 | 6 | 19 | 1.83E-02 | |

| | | | | | | |
|---|---|---|---|---|---|---|
| Photoprotection | GO | GO:0010117 | 5 | 13 | 2.49E-02 | |
| Response to herbicide | GO | GO:0009635 | 5 | 8 | 6.53E-03 | |
| Oxidoreductase activity | GO | GO:0016491 | 114 | 2048 | 2.37E-02 | Molecular Function |
| Lyase activity | GO | GO:0016829 | 39 | 545 | 3.22E-02 | |
| UDP-glycosyltransferase activity | GO | GO:0008194 | 34 | 414 | 1.11E-02 | |
| Monooxygenase activity | GO | GO:0004497 | 27 | 335 | 3.97E-02 | |
| Glucosyltransferase activity | GO | GO:0046527 | 24 | 231 | 5.34E-03 | |
| UDP-glucosyltransferase activity | GO | GO:0035251 | 24 | 217 | 2.81E-03 | |
| Anion transmembrane transporter activity | GO | GO:0008509 | 22 | 228 | 1.83E-02 | |
| Carbon-carbon lyase activity | GO | GO:0016830 | 18 | 179 | 3.10E-02 | |
| Inorganic anion transmembrane transporter activity | GO | GO:0015103 | 14 | 116 | 2.57E-02 | |
| Adenylyltransferase activity | GO | GO:0070566 | 9 | 44 | 1.22E-02 | |
| Transferase activity, transferring sulfur-containing groups | GO | GO:0016782 | 8 | 41 | 2.63E-02 | |
| Sulfotransferase activity | GO | GO:0008146 | 7 | 20 | 5.34E-03 | |
| Binding, bridging | GO | GO:0060090 | 6 | 24 | 3.83E-02 | |
| Flavonoid sulfotransferase activity | GO | GO:1990135 | 6 | 9 | 1.92E-03 | |
| Chloroplast | GO | GO:0009507 | 265 | 5468 | 3.22E-02 | Cellular Component |
| Plastid part | GO | GO:0044435 | 125 | 2188 | 6.53E-03 | |
| Chloroplast part | GO | GO:0044434 | 124 | 2160 | 6.42E-03 | |
| Plastid envelope | GO | GO:0009526 | 71 | 1132 | 1.74E-02 | |
| Chloroplast envelope | GO | GO:0009941 | 70 | 1107 | 1.60E-02 | |
| Cell surface | GO | GO:0009986 | 10 | 49 | 6.42E-03 | |

(a) Leaf - Ms up-regulated

| Term | Database | ID | No. of input | No. of background | Benjamini and Hochberg corrected $p$-value | GO category |
|---|---|---|---|---|---|---|
| Ribosome | KEGG | ath03010 | 35 | 536 | 2.78E-02 | - |
| Thiamine metabolism | KEGG | ath00730 | 5 | 14 | 2.53E-02 | - |
| Metabolic process | GO | GO:0008152 | 581 | 15444 | 3.07E-02 | |
| Response to stress | GO | GO:0006950 | 222 | 5100 | 7.51E-03 | |
| Organonitrogen compound metabolic process | GO | GO:1901564 | 126 | 2762 | 3.71E-02 | |

| | | | | | | |
|---|---|---|---|---|---|---|
| Organonitrogen compound biosynthetic process | GO | GO:1901566 | 95 | 1981 | 3.92E-02 | Biological Process |
| Response to temperature stimulus | GO | GO:0009266 | 65 | 904 | 2.17E-05 | |
| Response to cold | GO | GO:0009409 | 46 | 632 | 7.81E-04 | |
| Response to oxidative stress | GO | GO:0006979 | 39 | 644 | 3.92E-02 | |
| Response to fungus | GO | GO:0009620 | 36 | 486 | 4.60E-03 | |
| Defense response to fungus | GO | GO:0050832 | 27 | 376 | 3.07E-02 | |
| RNA modification | GO | GO:0009451 | 24 | 182 | 3.26E-05 | |
| Rhythmic process | GO | GO:0048511 | 18 | 216 | 4.16E-02 | |
| Circadian rhythm | GO | GO:0007623 | 17 | 201 | 4.86E-02 | |
| Cellular response to oxidative stress | GO | GO:0034599 | 13 | 117 | 2.86E-02 | |
| RNA secondary structure unwinding | GO | GO:0010501 | 12 | 52 | 2.73E-04 | |
| Pyrimidine-containing compound metabolic process | GO | GO:0072527 | 11 | 77 | 1.45E-02 | |
| Carbohydrate mediated signaling | GO | GO:0009756 | 10 | 74 | 3.07E-02 | |
| Pyrimidine-containing compound biosynthetic process | GO | GO:0072528 | 10 | 66 | 1.82E-02 | |
| Sugar mediated signaling pathway | GO | GO:0010182 | 10 | 74 | 3.07E-02 | |
| mRNA modification | GO | GO:0016556 | 9 | 43 | 5.97E-03 | |
| Chemotaxis | GO | GO:0006935 | 8 | 52 | 4.08E-02 | |
| Jasmonic acid biosynthetic process | GO | GO:0009695 | 8 | 51 | 3.92E-02 | |
| Pollen tube guidance | GO | GO:0010183 | 8 | 52 | 4.08E-02 | |
| Positive chemotaxis | GO | GO:0050918 | 8 | 52 | 4.08E-02 | |
| Taxis | GO | GO:0042330 | 8 | 52 | 4.08E-02 | |
| Thiamine biosynthetic process | GO | GO:0009228 | 5 | 14 | 2.53E-02 | |
| Thiamine metabolic process | GO | GO:0006772 | 5 | 16 | 3.28E-02 | |
| Thiamine-containing compound biosynthetic process | GO | GO:0042724 | 5 | 14 | 2.53E-02 | |
| Thiamine-containing compound metabolic process | GO | GO:0042723 | 5 | 16 | 3.28E-02 | |
| Structural molecule activity | GO | GO:0005198 | 48 | 808 | 2.33E-02 | Molecular Function |
| Structural constituent of ribosome | GO | GO:0003735 | 43 | 628 | 4.60E-03 | |
| ATP-dependent helicase activity | GO | GO:0008026 | 15 | 153 | 3.09E-02 | |
| Purine NTP-dependent helicase activity | GO | GO:0070035 | 15 | 153 | 3.09E-02 | |
| RNA helicase activity | GO | GO:0003724 | 12 | 80 | 5.97E-03 | |
| ATP-dependent RNA helicase activity | GO | GO:0004004 | 11 | 64 | 4.89E-03 | |
| RNA-dependent atpase activity | GO | GO:0008186 | 11 | 64 | 4.89E-03 | |

| | | | | | | |
|---|---|---|---|---|---|---|
| Cytoplasm | GO | GO:0005737 | 663 | 16748 | 9.13E-06 | Cellular Component |
| Cytoplasmic part | GO | GO:0044444 | 585 | 13923 | 2.41E-08 | |
| Plastid | GO | GO:0009536 | 309 | 5582 | 4.74E-15 | |
| Chloroplast | GO | GO:0009507 | 301 | 5468 | 1.72E-14 | |
| Mitochondrion | GO | GO:0005739 | 169 | 3376 | 1.17E-04 | |
| Plastid part | GO | GO:0044435 | 131 | 2188 | 5.49E-07 | |
| Chloroplast part | GO | GO:0044434 | 127 | 2160 | 2.30E-06 | |
| Plastid stroma | GO | GO:0009532 | 77 | 1187 | 5.36E-05 | |
| Chloroplast stroma | GO | GO:0009570 | 72 | 1159 | 4.48E-04 | |
| Thylakoid | GO | GO:0009579 | 56 | 875 | 2.15E-03 | |
| Chloroplast thylakoid | GO | GO:0009534 | 50 | 738 | 1.65E-03 | |
| Plastid thylakoid | GO | GO:0031976 | 50 | 738 | 1.65E-03 | |
| Ribosome | GO | GO:0005840 | 44 | 700 | 1.45E-02 | |
| Thylakoid part | GO | GO:0044436 | 43 | 652 | 7.43E-03 | |
| Photosynthetic membrane | GO | GO:0034357 | 41 | 602 | 5.97E-03 | |
| Thylakoid membrane | GO | GO:0042651 | 41 | 601 | 5.97E-03 | |
| Chloroplast thylakoid membrane | GO | GO:0009535 | 39 | 578 | 9.18E-03 | |
| Plastid thylakoid membrane | GO | GO:0055035 | 39 | 578 | 9.18E-03 | |
| Nucleoid | GO | GO:0009295 | 10 | 69 | 2.36E-02 | |
| Plastid nucleoid | GO | GO:0042646 | 8 | 49 | 3.36E-02 | |

(a) Flower petal - Ms up-regulated

| Term | Database | ID | No. of input | No. of background | Benjamini and Hochberg corrected *p*-value | GO category |
|---|---|---|---|---|---|---|
| Biosynthesis of secondary metabolites | KEGG | ath01110 | 56 | 1715 | 3.99E-02 | - |
| Phenylpropanoid biosynthesis | KEGG | ath00940 | 22 | 205 | 7.35E-07 | - |
| Stilbenoid, diarylheptanoid and gingerol biosynthesis | KEGG | ath00945 | 10 | 51 | 5.85E-05 | - |
| Flavonoid biosynthesis | KEGG | ath00941 | 7 | 52 | 1.10E-02 | - |
| Phenylalanine metabolism | KEGG | ath00360 | 7 | 60 | 1.82E-02 | - |
| Limonene and pinene degradation | KEGG | ath00903 | 6 | 40 | 1.57E-02 | - |
| Single-organism process | GO | GO:0044699 | 316 | 12768 | 1.65E-02 | |

| | | | | | | |
|---|---|---|---|---|---|---|
| Response to stimulus | GO | GO:0050896 | 272 | 8776 | 1.71E-09 | Biological Process |
| Single-organism metabolic process | GO | GO:0044710 | 164 | 5306 | 5.82E-05 | |
| Response to stress | GO | GO:0006950 | 160 | 4894 | 3.31E-06 | |
| Response to chemical | GO | GO:0042221 | 135 | 4288 | 2.83E-04 | |
| Defense response | GO | GO:0006952 | 85 | 2074 | 2.16E-06 | |
| Single-organism biosynthetic process | GO | GO:0044711 | 82 | 2450 | 2.73E-03 | |
| Oxidation-reduction process | GO | GO:0055114 | 81 | 2042 | 1.46E-05 | |
| Response to external stimulus | GO | GO:0009605 | 72 | 2045 | 2.25E-03 | |
| Response to biotic stimulus | GO | GO:0009607 | 60 | 1538 | 8.33E-04 | |
| Secondary metabolic process | GO | GO:0019748 | 53 | 548 | 2.98E-15 | |
| Response to external biotic stimulus | GO | GO:0043207 | 50 | 1482 | 3.91E-02 | |
| Response to inorganic substance | GO | GO:0010035 | 50 | 1458 | 2.98E-02 | |
| Response to other organism | GO | GO:0051707 | 50 | 1482 | 3.91E-02 | |
| Defense response to other organism | GO | GO:0098542 | 38 | 1032 | 3.59E-02 | |
| Secondary metabolite biosynthetic process | GO | GO:0044550 | 38 | 403 | 2.66E-10 | |
| Response to metal ion | GO | GO:0010038 | 35 | 811 | 5.94E-03 | |
| Phenylpropanoid metabolic process | GO | GO:0009698 | 33 | 324 | 1.02E-09 | |
| Response to oxidative stress | GO | GO:0006979 | 28 | 615 | 1.13E-02 | |
| Phenylpropanoid biosynthetic process | GO | GO:0009699 | 27 | 257 | 3.04E-08 | |
| Lignin metabolic process | GO | GO:0009808 | 24 | 189 | 1.22E-08 | |
| Response to fungus | GO | GO:0009620 | 21 | 460 | 3.99E-02 | |
| Response to wounding | GO | GO:0009611 | 21 | 379 | 6.18E-03 | |
| Flavonoid biosynthetic process | GO | GO:0009813 | 19 | 262 | 8.10E-04 | |
| Flavonoid metabolic process | GO | GO:0009812 | 19 | 293 | 2.51E-03 | |
| Lignin biosynthetic process | GO | GO:0009809 | 19 | 159 | 1.29E-06 | |
| Post-embryonic root development | GO | GO:0048528 | 14 | 220 | 1.70E-02 | |
| Response to transition metal nanoparticle | GO | GO:1990267 | 14 | 172 | 2.64E-03 | |
| Lateral root development | GO | GO:0048527 | 13 | 198 | 1.93E-02 | |
| Cellular response to inorganic substance | GO | GO:0071241 | 10 | 88 | 2.64E-03 | |
| Cellular response to iron ion | GO | GO:0071281 | 10 | 49 | 4.65E-05 | |
| Cellular response to metal ion | GO | GO:0071248 | 10 | 74 | 9.16E-04 | |
| Regulation of secondary metabolic process | GO | GO:0043455 | 10 | 75 | 9.84E-04 | |

| | | | | | | |
|---|---|---|---|---|---|---|
| Response to iron ion | GO | GO:0010039 | 10 | 67 | 4.65E-04 | |
| Response to toxic substance | GO | GO:0009636 | 10 | 127 | 2.22E-02 | |
| Hydrogen peroxide catabolic process | GO | GO:0042744 | 8 | 97 | 4.79E-02 | |
| Regulation of phenylpropanoid metabolic process | GO | GO:2000762 | 8 | 51 | 2.25E-03 | |
| Glutathione metabolic process | GO | GO:0006749 | 7 | 59 | 1.71E-02 | |
| Regulation of secondary metabolite biosynthetic process | GO | GO:1900376 | 7 | 42 | 3.65E-03 | |
| Toxin metabolic process | GO | GO:0009404 | 7 | 65 | 2.57E-02 | |
| Flavone biosynthetic process | GO | GO:0051553 | 6 | 27 | 3.09E-03 | |
| Flavone metabolic process | GO | GO:0051552 | 6 | 27 | 3.09E-03 | |
| Regulation of lignin biosynthetic process | GO | GO:1901141 | 6 | 23 | 2.04E-03 | |
| Secondary metabolite catabolic process | GO | GO:0090487 | 6 | 41 | 1.68E-02 | |
| Toxin catabolic process | GO | GO:0009407 | 6 | 41 | 1.68E-02 | |
| Flavonol biosynthetic process | GO | GO:0051555 | 5 | 24 | 1.36E-02 | |
| Flavonol metabolic process | GO | GO:0051554 | 5 | 24 | 1.36E-02 | |
| Cellular response to nitric oxide | GO | GO:0071732 | 4 | 12 | 1.31E-02 | |
| Response to nitric oxide | GO | GO:0071731 | 4 | 14 | 1.73E-02 | |
| Aluminum cation transport | GO | GO:0015690 | 3 | 6 | 2.39E-02 | |
| Atrichoblast differentiation | GO | GO:0010055 | 3 | 6 | 2.39E-02 | |
| Coumarin biosynthetic process | GO | GO:0009805 | 3 | 8 | 3.99E-02 | |
| Coumarin metabolic process | GO | GO:0009804 | 3 | 8 | 3.99E-02 | |
| Trivalent inorganic cation transport | GO | GO:0072512 | 3 | 6 | 2.39E-02 | |
| Oxidoreductase activity | GO | GO:0016491 | 80 | 1934 | 3.66E-06 | |
| Heme binding | GO | GO:0020037 | 33 | 389 | 3.89E-08 | |
| Oxidoreductase activity, acting on paired donors, with incorporation or reduction of molecular oxygen | GO | GO:0016705 | 33 | 420 | 2.11E-07 | |
| Tetrapyrrole binding | GO | GO:0046906 | 33 | 430 | 3.26E-07 | |
| Iron ion binding | GO | GO:0005506 | 29 | 359 | 8.63E-07 | |
| Monooxygenase activity | GO | GO:0004497 | 25 | 309 | 6.58E-06 | |
| Oxygen binding | GO | GO:0019825 | 23 | 226 | 7.42E-07 | |
| Dioxygenase activity | GO | GO:0051213 | 13 | 170 | 6.76E-03 | |
| Glucosyltransferase activity | GO | GO:0046527 | 13 | 226 | 4.79E-02 | |

| | | | | | | |
|---|---|---|---|---|---|---|
| S-adenosylmethionine-dependent methyltransferase activity | GO | GO:0008757 | 13 | 200 | 2.07E-02 | Molecular Function |
| Oxidoreductase activity, acting on paired donors, with incorporation or reduction of molecular oxygen, nad(p)h as one donor, and incorporation of one atom of oxygen | GO | GO:0016709 | 11 | 165 | 3.91E-02 | |
| Transferase activity, transferring alkyl or aryl (other than methyl) groups | GO | GO:0016765 | 11 | 161 | 3.36E-02 | |
| O-methyltransferase activity | GO | GO:0008171 | 9 | 48 | 2.83E-04 | |
| Oxidoreductase activity, acting on paired donors, with incorporation or reduction of molecular oxygen, 2-oxoglutarate as one donor, and incorporation of one atom each of oxygen into both donors | GO | GO:0016706 | 9 | 96 | 1.55E-02 | |
| Glutathione transferase activity | GO | GO:0004364 | 7 | 49 | 8.02E-03 | |
| D-ribose transmembrane transporter activity | GO | GO:0015591 | 5 | 15 | 2.73E-03 | |
| D-xylose transmembrane transporter activity | GO | GO:0015148 | 5 | 15 | 2.73E-03 | |
| Galactose transmembrane transporter activity | GO | GO:0005354 | 5 | 24 | 1.36E-02 | |
| Mannitol transmembrane transporter activity | GO | GO:0015575 | 5 | 15 | 2.73E-03 | |
| Myo-inositol transmembrane transporter activity | GO | GO:0005365 | 5 | 25 | 1.55E-02 | |
| Pentose transmembrane transporter activity | GO | GO:0015146 | 5 | 15 | 2.73E-03 | |
| Sorbitol transmembrane transporter activity | GO | GO:0015576 | 5 | 15 | 2.73E-03 | |
| Membrane part | GO | GO:0044425 | 207 | 7818 | 1.28E-02 | Cellular Component |
| Intrinsic component of membrane | GO | GO:0031224 | 199 | 7125 | 1.50E-03 | |
| Integral component of membrane | GO | GO:0016021 | 193 | 6887 | 1.70E-03 | |
| Cell periphery | GO | GO:0071944 | 189 | 6749 | 2.11E-03 | |
| Plasma membrane | GO | GO:0005886 | 161 | 5895 | 1.70E-02 | |
| Extracellular region | GO | GO:0005576 | 102 | 2627 | 1.10E-06 | |

**Table S5** Annotation of trichome-related DE transcripts between *M. sanguineum* and *M. candidum* . Bolded gene names represent tissue-specific DE genes.

(a) Leaf

| Up-regulated in | Gene Name | Other Names | Description | Annotations |
|---|---|---|---|---|
| Mc | **AT1G05230.4** | homeodomain GLABROUS 2 | Encodes a homeobox-leucine zipper family protein belonging to the HD-ZIP IV family. Mutants have trichomes that appear glass-like under a dissecting microscope as compared to the wild-type trichomes. The mutations do not affect trichome growth or branch number. | homeodomain GLABROUS 2 (HDG2); FUNCTIONS IN: DNA binding, sequence-specific DNA binding transcription factor activity; INVOLVED IN: regulation of transcription, DNA-dependent, trichome morphogenesis; LOCATED IN: nucleus; EXPRESSED IN: 32 plant structures; EXPRESSED DURING: 15 growth stages; CONTAINS InterPro DOMAIN/s: Homeobox, conserved site (InterPro:IPR017970), Homeobox (InterPro:IPR001356), Homeodomain-like (InterPro:IPR009057), Lipid-binding START (InterPro:IPR002913), Homeodomain-related (InterPro:IPR012287); BEST Arabidopsis thaliana protein match is: protodermal factor 2 (TAIR:AT4G04890.1). |
| Mc | **AT1G13180.1** | Actin-like ATPase superfamily protein | Mutant has defect in trichome cell expansion and actin organization resulting in a distorted trichome phenotype. | DISTORTED TRICHOMES 1 (DIS1); FUNCTIONS IN: actin binding, structural constituent of cytoskeleton, ATP binding; INVOLVED IN: actin filament organization, multidimensional cell growth, cell morphogenesis, trichome morphogenesis; LOCATED IN: Arp2/3 protein complex; EXPRESSED IN: 23 plant structures; EXPRESSED DURING: 14 growth stages; CONTAINS InterPro DOMAIN/s: Actin/actin-like (InterPro:IPR004000), Actin-related protein 3 (InterPro:IPR015623); BEST Arabidopsis thaliana protein match is: Actin-like ATPase superfamily protein (TAIR:AT2G42100.1) |
| Mc | AT1G15570.1 | CYCLIN A2;3 | A2-type cyclin. Negatively regulates endocycles and acts as a key regulator of ploidy levels in Arabidopsis endoreduplication. Interacts physically with CDKA;1. Expressed preferentially in trichomes and young developing tissues. | CYCLIN A2;3 (CYCA2;3); CONTAINS InterPro DOMAIN/s: Cyclin, C-terminal (InterPro:IPR004367), Cyclin (InterPro:IPR006670), G2/mitotic-specific cyclin A (InterPro:IPR015453), Cyclin-like (InterPro:IPR011028), Cyclin-related (InterPro:IPR013763), Cyclin, N-terminal (InterPro:IPR006671), Cyclin, A/B/D/E (InterPro:IPR014400); BEST Arabidopsis thaliana protein match is: Cyclin A2;4 (TAIR:AT1G80370.1) |

| | | | | |
|---|---|---|---|---|
| Mc | AT1G63650.3 | basic helix-loop-helix (bHLH) DNA-binding superfamily protein | Mutant has reduced trichomes, anthocyanin, and seed coat mucilage and abnormally patterned stomates. Mutants are defective in jasmonate-induced anthocyanin accumulation. Encodes a bHLH Transcription Factor 1. The protein is functionally redundant with GL3 and TT8 and interacts with TTG1, the myb proteins GL1, PAP1 and 2, CPC and TRY, and it will form heterodimers with GL3. Expression in N (non-hair cell forming) cell layers is negatively regulated by WER. Expression in H cells (hair cell forming) is promoted by CPC/TRY. | ENHANCER OF GLABRA 3 (EGL3); FUNCTIONS IN: DNA binding, sequence-specific DNA binding transcription factor activity; INVOLVED IN: epidermal cell fate specification, regulation of transcription; LOCATED IN: nucleus; EXPRESSED IN: 14 plant structures; EXPRESSED DURING: 7 growth stages; CONTAINS InterPro DOMAIN/s: Helix-loop-helix DNA-binding domain (InterPro:IPR001092), Helix-loop-helix DNA-binding (InterPro:IPR011598); BEST Arabidopsis thaliana protein match is: basic helix-loop-helix (bHLH) DNA-binding superfamily protein (TAIR:AT5G41315.1). |
| Mc | AT1G64670.1 | alpha/beta-Hydrolases superfamily protein | Encodes a epidermally expressed extracellular protein that likely functions as an alpha-beta hydrolase and is required for normal cuticle formation. Homozygous mutant plants are dwarfed and have abnormal leaves, collapsed cells, reduced numbers of trichomes. The specific role of BDG is unclear: it may function in cutin biosynthesis or as a cross-linking enzyme in the cell wall itself. | BODYGUARD1 (BDG1); CONTAINS InterPro DOMAIN/s: Alpha/beta hydrolase fold-1 (InterPro:IPR000073); BEST Arabidopsis thaliana protein match is: alpha/beta-Hydrolases superfamily protein (TAIR:AT5G41900.1) |
| Mc | AT1G67030.1 | zinc finger protein 6 | Encodes a novel C2H2 zinc finger protein containing only a single zinc finger which plays a key role in regulating trichome development by integrating GA and cytokinin signaling. The mRNA is cell-to-cell mobile. | zinc finger protein 6 (ZFP6); FUNCTIONS IN: sequence-specific DNA binding transcription factor activity, zinc ion binding, nucleic acid binding; INVOLVED IN: regulation of transcription; LOCATED IN: cytosolic ribosome; EXPRESSED IN: 7 plant structures; EXPRESSED DURING: 4 anthesis, petal differentiation and expansion stage; CONTAINS InterPro DOMAIN/s: Zinc finger, C2H2-like (InterPro:IPR015880), Zinc finger, C2H2-type (InterPro:IPR007087); BEST Arabidopsis thaliana protein match is: C2H2 and C2HC zinc fingers superfamily protein (TAIR:AT1G68360.1) |
| Mc | **AT1G68360.1** | C2H2 and C2HC zinc fingers superfamily protein | Encodes a nuclear localized member of the C2H2 family of TFIIIA transcription factors. GIS3 is involved in trichome initiation and development downstream of GA and cytokinin signaling. GIS regulates the expression GIS and GIS2. | C2H2 and C2HC zinc fingers superfamily protein; FUNCTIONS IN: sequence-specific DNA binding transcription factor activity; INVOLVED IN: regulation of transcription; LOCATED IN: intracellular; EXPRESSED IN: 12 plant structures; EXPRESSED DURING: 4 anthesis, F mature embryo stage, petal differentiation and expansion stage, E expanded cotyledon stage, D bilateral stage; CONTAINS InterPro DOMAIN/s: Zinc finger, C2H2-like (InterPro:IPR015880), Zinc finger, C2H2-type (InterPro:IPR007087); BEST Arabidopsis thaliana protein match is: zinc finger protein 6 (TAIR:AT1G67030.1) |

| | | | | |
|---|---|---|---|---|
| Mc | **AT2G19520.1** | Transducin family protein / WD-40 repeat family protein | Controls flowering. | FVE; FUNCTIONS IN: metal ion binding; INVOLVED IN: flower development, unidimensional cell growth, trichome morphogenesis, leaf morphogenesis; LOCATED IN: nucleolus, nucleus, CUL4 RING ubiquitin ligase complex, cytoplasm; EXPRESSED IN: guard cell; CONTAINS InterPro DOMAIN/s: Histone-binding protein RBBP4 (InterPro:IPR022052), WD40 repeat 2 (InterPro:IPR019782), WD40 repeat-like-containing domain (InterPro:IPR011046), WD40-repeat-containing domain (InterPro:IPR017986), WD40/YVTN repeat-like-containing domain (InterPro:IPR015943), WD40 repeat (InterPro:IPR001680), WD40 repeat, subgroup (InterPro:IPR019781); BEST Arabidopsis thaliana protein match is: nucleosome/chromatin assembly factor group C5 (TAIR:AT4G29730.1) |
| Mc | AT2G30420.1 | Homeodomain-like superfamily protein | In a tandem repeat with AT2G30424 and AT2G30432 | ENHANCER OF TRY AND CPC 2 (ETC2); FUNCTIONS IN: DNA binding, sequence-specific DNA binding transcription factor activity; INVOLVED IN: trichome patterning, regulation of transcription; LOCATED IN: mitochondrion; CONTAINS InterPro DOMAIN/s: SANT, DNA-binding (InterPro:IPR001005), Myb, DNA-binding (InterPro:IPR014778), Homeodomain-related (InterPro:IPR012287), Myb transcription factor (InterPro:IPR015495); BEST Arabidopsis thaliana protein match is: Homeodomain-like superfamily protein (TAIR:AT2G30424.1) |
| Mc | AT3G01140.1 | myb domain protein 106 | Encodes a MIXTA-like MYB gene NOECK (NOK). Loss of function mutations show an increased number of branchpoints in leaf trichomes suggesting a role in negative regulation of trichome branching. | myb domain protein 106 (MYB106); FUNCTIONS IN: DNA binding, sequence-specific DNA binding transcription factor activity; INVOLVED IN: trichome branching, regulation of transcription, DNA-dependent; EXPRESSED IN: 18 plant structures; EXPRESSED DURING: 7 growth stages; CONTAINS InterPro DOMAIN/s: SANT, DNA-binding (InterPro:IPR001005), Homeodomain-like (InterPro:IPR009057), Myb, DNA-binding (InterPro:IPR014778), Myb transcription factor (InterPro:IPR015495), Homeodomain-related (InterPro:IPR012287), HTH transcriptional regulator, Myb-type, DNA-binding (InterPro:IPR017930); BEST Arabidopsis thaliana protein match is: myb domain protein 16 (TAIR:AT5G15310.1) |
| Mc | AT3G13540.1 | myb domain protein 5 | Encodes a member of the MYB family of transcriptional regulators. MYB5 act as a negative regulator of trichome branching and play a role in the correct formation of the seed coat and possibly the formation the underlying endosperm layers. Loss of function mutations have defects in seed coat mucilage and columella cells as well as trichome defects (smaller and reduced number of branches). | myb domain protein 5 (MYB5); CONTAINS InterPro DOMAIN/s: SANT, DNA-binding (InterPro:IPR001005), Homeodomain-like (InterPro:IPR009057), Myb, DNA-binding (InterPro:IPR014778), HTH transcriptional regulator, Myb-type, DNA-binding (InterPro:IPR017930), Homeodomain-related (InterPro:IPR012287), Myb transcription factor (InterPro:IPR015495); BEST Arabidopsis thaliana protein match is: myb domain protein 17 (TAIR:AT3G61250.1) |

| | | | | |
|---|---|---|---|---|
| Mc | **AT3G58070.1** | C2H2 and C2HC zinc fingers superfamily protein | Putative transcription factor, contains C2H2 domain, regulates aspects of shoot maturation in Arabidopsis thaliana. GIS loss-of-function mutations affect the epidermal differentiation of inflorescence organs, causing a premature decrease in trichome production on successive leaves, stem internodes, and branches. Overexpression has the opposite effect on trichome initiation and causes other heterochronic phenotypes, affecting flowering and juvenile–adult leaf transition and inducing the formation of rosette leaves on inflorescence stems. | GLABROUS INFLORESCENCE STEMS (GIS); FUNCTIONS IN: sequence-specific DNA binding transcription factor activity, zinc ion binding, nucleic acid binding; INVOLVED IN: trichome differentiation, trichome branching, response to gibberellin stimulus, regulation of transcription; LOCATED IN: intracellular; EXPRESSED IN: 12 plant structures; EXPRESSED DURING: 7 growth stages; CONTAINS InterPro DOMAIN/s: Zinc finger, C2H2-like (InterPro:IPR015880), Zinc finger, C2H2-type (InterPro:IPR007087); BEST Arabidopsis thaliana protein match is: zinc finger protein 8 (TAIR:AT2G41940.1) |
| Mc | **AT4G09820.1** | basic helix-loop-helix (bHLH) DNA-binding superfamily protein | TT8 is a regulation factor that acts in a concerted action with TT1, PAP1 and TTG1 on the regulation of flavonoid pathways, namely proanthocyanidin and anthocyanin biosynthesis.  Affects dihydroflavonol 4-reductase gene expression. It is thought that a ternary complex composed of TT2, TT8 and TTG1 is necessary for correct expression of BAN in seed endothelium. Also important for important for marginal trichome development. It binds the promoter of both AT3G26790 and AT1G28300.TT8 interacts with JAZ  proteins to regulate anthocyanin accumulation. | TRANSPARENT TESTA 8 (TT8); FUNCTIONS IN: DNA binding, sequence-specific DNA binding transcription factor activity; INVOLVED IN: trichome differentiation, regulation of flavonoid biosynthetic process, regulation of proanthocyanidin biosynthetic process; LOCATED IN: nucleus; EXPRESSED IN: stem, hypocotyl, micropylar endosperm; EXPRESSED DURING: C globular stage; CONTAINS InterPro DOMAIN/s: Helix-loop-helix DNA-binding domain (InterPro:IPR001092), Helix-loop-helix DNA-binding (InterPro:IPR011598); BEST Arabidopsis thaliana protein match is: basic helix-loop-helix (bHLH) DNA-binding superfamily protein (TAIR:AT1G63650.3) |
| Mc | **AT4G16340.1** | guanyl-nucleotide exchange factors;GTPase binding;GTP binding | Encodes SPIKE1 (SPK1), the lone DOCK family guanine nucleotide exchange factor (GEF) in Arabidopsis.  SPK1 is a peripheral membrane protein that accumulates at, and promotes the formation of, a specialized domain of the endoplasmic reticulum (ER) termed the ER exit site (ERES). SPK1 promotes polarized growth and cell-cell adhesion in the leaf epidermis. Mutant has seedling lethal; cotyledon, leaf-shape, trichome defects. | SPIKE1 (SPK1); FUNCTIONS IN: GTPase binding, GTP binding, guanyl-nucleotide exchange factor activity; LOCATED IN: cytosol, plasma membrane; EXPRESSED IN: 22 plant structures; EXPRESSED DURING: 13 growth stages; CONTAINS InterPro DOMAIN/s: Dedicator of cytokinesis (InterPro:IPR010703) |
| Mc | **AT4G22910.1** | FIZZY-related 2 | NULL | FIZZY-related 2 (FZR2); FUNCTIONS IN: signal transducer activity; INVOLVED IN: trichome branching, signal transduction, DNA endoreduplication, cell growth; LOCATED IN: chloroplast, heterotrimeric G-protein complex; EXPRESSED IN: 24 plant structures; EXPRESSED DURING: 14 growth stages; CONTAINS InterPro DOMAIN/s: WD40 repeat 2 (InterPro:IPR019782), WD40 repeat-like-containing domain (InterPro:IPR011046), WD40 repeat, conserved site (InterPro:IPR019775), WD40-repeat-containing domain (InterPro:IPR017986), WD40/YVTN repeat-like-containing domain (InterPro:IPR015943), WD40 repeat (InterPro:IPR001680), WD40 repeat, subgroup (InterPro:IPR019781); BEST Arabidopsis thaliana protein match is: cell cycle switch protein 52 A2 (TAIR:AT4G11920.1) |

| | | | | |
|---|---|---|---|---|
| Mc | AT5G05170.1 | Cellulose synthase family protein | Encodes a cellulose synthase isomer. CESA3 mutants have cellulose defect in the primary cell wall. Multiple lines of evidence suggest that CESA3, along with CESA1 and CESA6 are present in the same plasma membrane complex for cellulose biosynthesis. As inferred from the null role of secondary wall-type CesAs, included in a set of five primary wall-type CesAs that may support trichome cell wall thickening. The xylem cells in primary root have reduced cell expansion and higher than normal lignification. | CONSTITUTIVE EXPRESSION OF VSP 1 (CEV1); FUNCTIONS IN: cellulose synthase activity, transferase activity, transferring glycosyl groups; INVOLVED IN: primary cell wall biogenesis, cellulose biosynthetic process, defense response; LOCATED IN: Golgi apparatus, plasma membrane; EXPRESSED IN: 27 plant structures; EXPRESSED DURING: 14 growth stages; CONTAINS InterPro DOMAIN/s: Cellulose synthase (InterPro:IPR005150), Zinc finger, RING-type (InterPro:IPR001841); BEST Arabidopsis thaliana protein match is: cellulose synthase 1 (TAIR:AT4G32410.1) |
| Mc | **AT5G06650.1** | C2H2 and C2HC zinc fingers superfamily protein | NULL | GLABROUS INFLORESCENCE STEMS 2 (GIS2); FUNCTIONS IN: sequence-specific DNA binding transcription factor activity, zinc ion binding, nucleic acid binding; INVOLVED IN: trichome differentiation, response to gibberellin stimulus, response to cytokinin stimulus, regulation of transcription; LOCATED IN: intracellular; EXPRESSED IN: 6 plant structures; EXPRESSED DURING: F mature embryo stage, petal differentiation and expansion stage, E expanded cotyledon stage, D bilateral stage; CONTAINS InterPro DOMAIN/s: Zinc finger, C2H2-like (InterPro:IPR015880), Zinc finger, C2H2-type (InterPro:IPR007087); BEST Arabidopsis thaliana protein match is: C2H2 and C2HC zinc fingers superfamily protein (TAIR:AT3G58070.1) |
| Mc | **AT5G20490.1** | Myosin family protein with Dil domain | Encodes a member of the type XI myosin protein family involved in root hair growth, trichome development, and organelle trafficking. Required for fast root hair growth. This gene appears to be expressed at low levels throughout the plant. | XIK; FUNCTIONS IN: motor activity; INVOLVED IN: in 11 processes; LOCATED IN: endomembrane system, myosin complex; EXPRESSED IN: 6 plant structures; EXPRESSED DURING: seedling growth; CONTAINS InterPro DOMAIN/s: Dil domain (InterPro:IPR018444), Dilute (InterPro:IPR002710), Myosin, N-terminal, SH3-like (InterPro:IPR004009), Myosin head, motor domain (InterPro:IPR001609), IQ calmodulin-binding region (InterPro:IPR000048); BEST Arabidopsis thaliana protein match is: Myosin family protein with Dil domain (TAIR:AT1G54560.1) |
| Mc | AT5G28646.2 | TPX2 (targeting protein for Xklp2) protein family | Encodes a novel protein. The wvd2 gain-of-function mutant has impaired cell expansion and root waving, and changed root skewing. | WAVE-DAMPENED 2 (WVD2); FUNCTIONS IN: molecular_function unknown; INVOLVED IN: trichome branching, multidimensional cell growth, microtubule bundle formation, establishment or maintenance of cell polarity, microtubule cytoskeleton organization; LOCATED IN: cortical microtubule; CONTAINS InterPro DOMAIN/s: Xklp2 targeting protein (InterPro:IPR009675); BEST Arabidopsis thaliana protein match is: WVD2-like 1 (TAIR:AT3G04630.3) |

| | | | | |
|---|---|---|---|---|
| Mc | AT5G37770.1 | EF hand calcium-binding protein family | Encodes a protein with 40% similarity to calmodulin. Binds Ca(2+) and, as a consequence, undergoes conformational changes. CML24 expression occurs in all major organs, and transcript levels are increased from 2- to 15-fold in plants subjected to touch, darkness, heat, cold, hydrogen peroxide, abscisic acid (ABA), and indole-3-acetic acid. However, CML24 protein accumulation changes were not detectable. The putative CML24 regulatory region confers reporter expression at sites of predicted mechanical stress; in regions undergoing growth; in vascular tissues and various floral organs; and in stomata, trichomes, and hydathodes. CML24-underexpressing transgenics are resistant to ABA inhibition of germination and seedling growth, are defective in long-day induction of flowering, and have enhanced tolerance to CoCl(2), molybdic acid, ZnSO(4), and MgCl(2). Also regulates nitric oxide levels. | TOUCH 2 (TCH2); FUNCTIONS IN: calcium ion binding; INVOLVED IN: in 13 processes; LOCATED IN: plasma membrane, actin cytoskeleton; EXPRESSED IN: 34 plant structures; EXPRESSED DURING: 14 growth stages; CONTAINS InterPro DOMAIN/s: EF-Hand 1, calcium-binding site (InterPro:IPR018247), EF-HAND 2 (InterPro:IPR018249), EF-hand-like domain (InterPro:IPR011992), Calcium-binding EF-hand (InterPro:IPR002048), EF-hand (InterPro:IPR018248); BEST Arabidopsis thaliana protein match is: calmodulin like 23 (TAIR:AT1G66400.1) |
| Mc | **AT5G65930.2** | kinesin-like calmodulin-binding protein (ZWICHEL) | Encodes a novel member of the kinesin superfamily of motor proteins. recessive mutations have reduced number of trichome branches. | ZWICHEL (ZWI); FUNCTIONS IN: microtubule motor activity, calmodulin binding; INVOLVED IN: trichome branching, pollen germination; LOCATED IN: plasma membrane; EXPRESSED IN: 24 plant structures; EXPRESSED DURING: 15 growth stages; CONTAINS InterPro DOMAIN/s: FERM central domain (InterPro:IPR019748), Prismane-like (InterPro:IPR011254), MyTH4 domain (InterPro:IPR000857), Kinesin, motor domain (InterPro:IPR001752), FERM, N-terminal (InterPro:IPR018979), Band 4.1 domain (InterPro:IPR019749), Kinesin, motor region, conserved site (InterPro:IPR019821), FERM/acyl-CoA-binding protein, 3-helical bundle (InterPro:IPR014352), FERM domain (InterPro:IPR000299); BEST Arabidopsis thaliana protein match is: Di-glucose binding protein with Kinesin motor domain (TAIR:AT2G22610.1) |
| Mc | **AT5G65930.3** | kinesin-like calmodulin-binding protein (ZWICHEL) | Encodes a novel member of the kinesin superfamily of motor proteins. recessive mutations have reduced number of trichome branches. | ZWICHEL (ZWI); CONTAINS InterPro DOMAIN/s: FERM central domain (InterPro:IPR019748), Prismane-like (InterPro:IPR011254), MyTH4 domain (InterPro:IPR000857), Kinesin, motor domain (InterPro:IPR001752), Band 4.1 domain (InterPro:IPR019749), FERM, N-terminal (InterPro:IPR018979), Kinesin, motor region, conserved site (InterPro:IPR019821), FERM/acyl-CoA-binding protein, 3-helical bundle (InterPro:IPR014352), FERM domain (InterPro:IPR000299); BEST Arabidopsis thaliana protein match is: Di-glucose binding protein with Kinesin motor domain (TAIR:AT2G22610.2). |
| Ms | AT1G02065.1 | squamosa promoter binding protein-like 8 | Encodes an SBP-box gene, a member of the SPL gene family. Mutants are affected in micro- and megasporogenesis, trichome formation on sepals, and stamen filament elongation. | squamosa promoter binding protein-like 8 (SPL8); CONTAINS InterPro DOMAIN/s: Transcription factor, SBP-box (InterPro:IPR004333); BEST Arabidopsis thaliana protein match is: squamosa promoter binding protein-like 2 (TAIR:AT5G43270.1) |

| | | | | |
|---|---|---|---|---|
| Ms | **AT1G79840.2** | HD-ZIP IV family of homeobox-leucine zipper protein with lipid-binding START domain | Glabra 2, a homeodomain protein affects epidermal cell identity including trichomes, root hairs, and seed coat. It also down-regulates seed oil content. Expressed in atrichoblasts and required to suppress root hair development. Also expressed abundantly during early seed development. Directly regulated by WER. | GLABRA 2 (GL2); CONTAINS InterPro DOMAIN/s: Homeobox, conserved site (InterPro:IPR017970), Homeobox (InterPro:IPR001356), Homeodomain-like (InterPro:IPR009057), Lipid-binding START (InterPro:IPR002913), Homeodomain-related (InterPro:IPR012287); BEST Arabidopsis thaliana protein match is: protodermal factor 2 (TAIR:AT4G04890.1). |
| Ms | AT1G80350.1 | P-loop containing nucleoside triphosphate hydrolases superfamily protein | Encodes a p60 katanin protein that is expressed throughout the plant. Required for the specification of cell fates from early in development (in the meristem) through differentiation and for normal postmitotic organization of cortical microtubules into transverse arrays in root epidermis cells. Mutants display cytoskeletal defects. | ECTOPIC ROOT HAIR 3 (ERH3); FUNCTIONS IN: nucleoside-triphosphatase activity, nucleotide binding, ATP binding; INVOLVED IN: trichome branching, cortical microtubule organization, multidimensional cell growth, plant-type cell wall biogenesis, microtubule cytoskeleton organization; LOCATED IN: katanin complex; EXPRESSED IN: 23 plant structures; EXPRESSED DURING: 15 growth stages; CONTAINS InterPro DOMAIN/s: ATPase, AAA-type, core (InterPro:IPR003959), ATPase, AAA+ type, core (InterPro:IPR003593), ATPase, AAA-type, conserved site (InterPro:IPR003960), Vps4 oligomerisation, C-terminal (InterPro:IPR015415); BEST Arabidopsis thaliana protein match is: P-loop containing nucleoside triphosphate hydrolases superfamily protein (TAIR:AT2G34560.1) |
| Ms | **AT2G02480.1** | AAA-type ATPase family protein | STICHEL mutant shows trichomes with fewer than normal branches. | STICHEL (STI); FUNCTIONS IN: DNA binding, DNA-directed DNA polymerase activity, ATP binding; INVOLVED IN: trichome differentiation, trichome branching; LOCATED IN: plasma membrane; EXPRESSED IN: 20 plant structures; EXPRESSED DURING: 11 growth stages; CONTAINS InterPro DOMAIN/s: ATPase, AAA-type, core (InterPro:IPR003959), DNA polymerase III, clamp loader complex, gamma/delta/delta subunit, C-terminal (InterPro:IPR008921), DNA polymerase III, subunit gamma/ tau (InterPro:IPR012763); BEST Arabidopsis thaliana protein match is: AAA-type ATPase family protein (TAIR:AT1G14460.1) |
| Ms | AT2G41100.3 | Calcium-binding EF hand family protein | Encodes a calmodulin-like protein, with six potential calcium binding domains. Calcium binding shown by Ca(2+)-specific shift in electrophoretic mobility. Expression induced by touch and darkness. Expression may also be developmentally controlled. Expression in growing regions of roots, vascular tissue, root/shoot junctions, trichomes, branch points of the shoot, and regions of siliques and flowers. The mRNA is cell-to-cell mobile. | TOUCH 3 (TCH3); FUNCTIONS IN: calcium ion binding; INVOLVED IN: response to mechanical stimulus, response to temperature stimulus, thigmotropism, response to absence of light; LOCATED IN: plasma membrane; EXPRESSED IN: 26 plant structures; EXPRESSED DURING: 14 growth stages; CONTAINS InterPro DOMAIN/s: EF-Hand 1, calcium-binding site (InterPro:IPR018247), EF-HAND 2 (InterPro:IPR018249), EF-hand-like domain (InterPro:IPR011992), Calcium-binding EF-hand (InterPro:IPR002048), EF-hand (InterPro:IPR018248); BEST Arabidopsis thaliana protein match is: calmodulin 5 (TAIR:AT2G27030.3) |

| | | | | |
|---|---|---|---|---|
| Ms | AT3G09790.1 | ubiquitin 8 | Encodes a ubiquitin-like protein that contains tandem repeats of the ubiquitin coding region, but at least one repeat per gene encodes a protein with amino acid substitutions. | ubiquitin 8 (UBQ8); INVOLVED IN: protein modification process, ubiquitin-dependent protein catabolic process; LOCATED IN: cell wall, intracellular, vacuole; EXPRESSED IN: guard cell, juvenile leaf, trichome; CONTAINS InterPro DOMAIN/s: Ubiquitin subgroup (InterPro:IPR019956), Ubiquitin (InterPro:IPR000626), Ubiquitin supergroup (InterPro:IPR019955); BEST Arabidopsis thaliana protein match is: polyubiquitin 10 (TAIR:AT4G05320.4) |
| Ms | AT3G45290.1 | Seven transmembrane MLO family protein | A member of a large family of seven-transmembrane domain proteins specific to plants, homologs of the barley mildew resistance locus o (MLO) protein. The Arabidopsis genome contains 15 genes encoding MLO proteins, with localization in plasma membrane. Phylogenetic analysis revealed four clades of closely-related AtMLO genes. ATMLO3 belongs to the clade IV, with AtMLO2, AtMLO6 and AtMLO12. The gene is expressed during early seedling growth, in primary root and lateral root primordia, in fruit abscission zone, in vascular system of cotyledons and in trichomes of young leaves,; it was not expressed in mature rosette leaves, as shown by GUS activity patterns. The expression of several phylogenetically closely-related AtMLO genes showed similar or overlapping tissue specificity and analogous responsiveness to external stimuli, suggesting functional redundancy, co-function, or antagonistic function(s). | MILDEW RESISTANCE LOCUS O 3 (MLO3); FUNCTIONS IN: calmodulin binding; INVOLVED IN: cell death, defense response; LOCATED IN: integral to membrane, plasma membrane; EXPRESSED IN: 29 plant structures; EXPRESSED DURING: 14 growth stages; CONTAINS InterPro DOMAIN/s: Mlo-related protein (InterPro:IPR004326); BEST Arabidopsis thaliana protein match is: Seven transmembrane MLO family protein (TAIR:AT2G39200.1) |
| Ms | **AT3G57230.2** | AGAMOUS-like 16 | MADS-box transcription factor. Expressed in leaf, root and stem, with higher RNA accumulation in guard cells and trichomes. AGL16 can directly interact with SVP and indirectly interact with FLC. Furthermore, the accumulation of AGL16 transcripts is modulated by miR824 (AT4G24415). The flowering time effect for the miR824/AGL16 module is more obvious in the Col-FRI background than in the Col-0 background. AGL16 controls flowering via a allelic dosage effect in long-day non-vernalized conditions. | AGAMOUS-like 16 (AGL16); FUNCTIONS IN: sequence-specific DNA binding transcription factor activity; INVOLVED IN: regulation of transcription, DNA-dependent, stomatal lineage progression; LOCATED IN: nucleus; EXPRESSED IN: 26 plant structures; EXPRESSED DURING: 13 growth stages; CONTAINS InterPro DOMAIN/s: Transcription factor, MADS-box (InterPro:IPR002100), Transcription factor, K-box (InterPro:IPR002487); BEST Arabidopsis thaliana protein match is: AGAMOUS-like 21 (TAIR:AT4G37940.1) |
| Ms | **AT4G00100.1** | ribosomal protein S13A | Encodes a cytoplasmic ribosomal protein S13 homologue involved in early leaf development The mRNA is cell-to-cell mobile. | ribosomal protein S13A (RPS13A); FUNCTIONS IN: structural constituent of ribosome; INVOLVED IN: translation, trichome morphogenesis, leaf morphogenesis, cytokinesis by cell plate formation; LOCATED IN: cytosolic small ribosomal subunit, nucleolus, membrane; EXPRESSED IN: 24 plant structures; EXPRESSED DURING: 14 growth stages; CONTAINS InterPro DOMAIN/s: Ribosomal protein S13/S15, N-terminal (InterPro:IPR012606), Ribosomal protein S15 (InterPro:IPR000589), S15/NS1, RNA-binding (InterPro:IPR009068); BEST Arabidopsis thaliana protein match is: Ribosomal protein S13/S15 (TAIR:AT3G60770.1) |

| | | | | |
|---|---|---|---|---|
| Ms | **AT4G18770.1** | myb domain protein 98 | MYB98 is a member of the R2R3-MYB gene family, the members of which likely encode transcription factors. Within an ovule, MYB98 is expressed exclusively in the synergid cells, and mutations in this gene affect the female gametophyte specifically. myb98 female gametophytes are affected in two unique features of the synergid cell, pollen tube guidance and the filiform apparatus, but are otherwise normal. This suggests that MYB98 controls the development of specific features within the synergid cell during female gametophyte development. MYB98 also is expressed in trichomes and endosperm. Homozygous myb98 mutants exhibit no sporophytic defects, including trichome and endosperm defects. | myb domain protein 98 (MYB98); CONTAINS InterPro DOMAIN/s: SANT, DNA-binding (InterPro:IPR001005), Homeodomain-like (InterPro:IPR009057), Myb, DNA-binding (InterPro:IPR014778), HTH transcriptional regulator, Myb-type, DNA-binding (InterPro:IPR017930), Homeodomain-related (InterPro:IPR012287), Myb transcription factor (InterPro:IPR015495); BEST Arabidopsis thaliana protein match is: myb domain protein 118 (TAIR:AT3G27785.1) |
| Ms | AT4G32410.1 | cellulose synthase 1 | Encodes a cellulose synthase isomer. CESA1 mutants have cellulose defect in the primary cell wall. Multiple lines of evidence suggest that CESA1, along with CESA3 and CESA6 are present in the same plasma membrane complex for cellulose biosynthesis. lasma membrane complex for cellulose biosynthesis. As inferred from the null role of secondary wall-type CesAs, included in a set of five primary wall-type CesAs that may support trichome cell wall thickening. | cellulose synthase 1 (CESA1); FUNCTIONS IN: cellulose synthase activity, transferase activity, transferring glycosyl groups; INVOLVED IN: primary cell wall biogenesis, cellulose biosynthetic process, plant-type cell wall biogenesis, hyperosmotic salinity response; LOCATED IN: Golgi apparatus, plasma membrane; EXPRESSED IN: 26 plant structures; EXPRESSED DURING: 16 growth stages; CONTAINS InterPro DOMAIN/s: Cellulose synthase (InterPro:IPR005150), Zinc finger, RING-type (InterPro:IPR001841); BEST Arabidopsis thaliana protein match is: cellulose synthase 10 (TAIR:AT2G25540.1) |
| Ms | **AT5G65690.1** | phosphoenolpyruvate carboxykinase 2 | Encodes a putative phosphoenolpyruvate carboxykinase (ATP-dependent). The mRNA is cell-to-cell mobile. | phosphoenolpyruvate carboxykinase 2 (PCK2); FUNCTIONS IN: phosphoenolpyruvate carboxykinase activity, purine nucleotide binding, phosphoenolpyruvate carboxykinase (ATP) activity, ATP binding; INVOLVED IN: gluconeogenesis; LOCATED IN: cellular_component unknown; EXPRESSED IN: stem, male gametophyte, root, flower, trichome; EXPRESSED DURING: seedling growth; CONTAINS InterPro DOMAIN/s: Phosphoenolpyruvate carboxykinase, N-terminal (InterPro:IPR008210), Phosphoenolpyruvate carboxykinase (ATP), conserved site (InterPro:IPR015994), Phosphoenolpyruvate carboxykinase, ATP-utilising (InterPro:IPR001272), Phosphoenolpyruvate carboxykinase, C-terminal (InterPro:IPR013035); BEST Arabidopsis thaliana protein match is: phosphoenolpyruvate carboxykinase 1 (TAIR:AT4G37870.1) |

| | | | | |
|---|---|---|---|---|
| both | AT1G21210.1 | wall associated kinase 4 | Cell wall-associated ser/thr kinase involved in cell elongation and lateral root development | wall associated kinase 4 (WAK4); FUNCTIONS IN: protein serine/threonine kinase activity, protein kinase activity, ATP binding, calcium ion binding; INVOLVED IN: protein amino acid phosphorylation, unidimensional cell growth, lateral root development; LOCATED IN: plasma membrane; EXPRESSED IN: root, trichome; CONTAINS InterPro DOMAIN/s: EGF-like calcium-binding (InterPro:IPR001881), EGF-like, type 3 (InterPro:IPR000742), Serine-threonine/tyrosine-protein kinase (InterPro:IPR001245), Protein kinase-like domain (InterPro:IPR011009), Serine/threonine-protein kinase, active site (InterPro:IPR008271), Protein kinase, catalytic domain (InterPro:IPR000719), EGF-like calcium-binding, conserved site (InterPro:IPR018097), EGF-type aspartate/asparagine hydroxylation site (InterPro:IPR000152), EGF calcium-binding (InterPro:IPR013091), EGF-like (InterPro:IPR006210); BEST Arabidopsis thaliana protein match is: wall-associated kinase 2 (TAIR:AT1G21270.1) |
| both | AT3G13530.1 | mitogen-activated protein kinase kinase kinase 7 | MAP3K epsilon protein kinase 1 is functionally redundant with MAP3Ke2. Required for pollen development but not essential. map3ke1;map3ke2 double-mutant pollen grains develop plasma membrane irregularities following pollen mitosis I. Localized primarily in the plasma membrane. Expressed in leaf trichomes, root columella cells and developing ovules. | mitogen-activated protein kinase kinase kinase 7 (MAPKKK7); FUNCTIONS IN: protein serine/threonine kinase activity, binding, protein kinase activity, kinase activity, ATP binding; INVOLVED IN: plasma membrane organization, pollen development; LOCATED IN: cytosol, plasma membrane; EXPRESSED IN: 6 plant structures; EXPRESSED DURING: ovule developmental stages; CONTAINS InterPro DOMAIN/s: Protein kinase, ATP binding site (InterPro:IPR017441), Serine/threonine-protein kinase domain (InterPro:IPR002290), Armadillo-like helical (InterPro:IPR011989), Armadillo (InterPro:IPR000225), Serine/threonine-protein kinase-like domain (InterPro:IPR017442), Protein kinase-like domain (InterPro:IPR011009), Serine/threonine-protein kinase, active site (InterPro:IPR008271), Protein kinase, catalytic domain (InterPro:IPR000719), Armadillo-type fold (InterPro:IPR016024); BEST Arabidopsis thaliana protein match is: mitogen-activated protein kinase kinase kinase 6 (TAIR:AT3G07980.1) |
| both | AT3G21560.1 | UDP-Glycosyltransferase superfamily protein | Encodes a protein with sinapic acid:UDP-glucose glucosyltransferase activity. Mutants defective in this gene are hyper-fluorescent (which accumulate in their trichomes a compound that is likely to be 3',5'-dimethoxynaringenin chalcone or sinapoyltriacetic acid lactone, potential products of the concerted action of 4-coumarate CoA ligase and chalcone synthase on sinapic acid). Also shown to be required for Arabidopsis nonhost resistance to the Asian soybean rust pathogen Phakopsora pachyrhizi. | UGT84A2; CONTAINS InterPro DOMAIN/s: UDP-glucuronosyl/UDP-glucosyltransferase (InterPro:IPR002213); BEST Arabidopsis thaliana protein match is: UDP-Glycosyltransferase superfamily protein (TAIR:AT4G15490.1) |

| | | | | |
|---|---|---|---|---|
| both | **AT3G44200.1** | NIMA (never in mitosis, gene A)-related 6 | Encodes AtNek5, a member of the NIMA-related serine/threonine kinases (Neks) that have been linked to cell-cycle regulation in fungi and mammals. Plant Neks might be involved in plant development processes.Interacts physically with plant kinesins ARK1 and ARK2. Mutants show defects in root epidermal cell morphology, trichome branching and other epidermal cell abnormalities suggesting a rol e in epidermal cell differentiation. NEK6 co-localizes with cortical microtubules. | NIMA (never in mitosis, gene A)-related 6 (NEK6); FUNCTIONS IN: protein serine/threonine kinase activity, protein kinase activity, kinase activity, ATP binding; INVOLVED IN: microtubule-based process, epidermal cell differentiation; LOCATED IN: cortical microtubule; EXPRESSED IN: 22 plant structures; EXPRESSED DURING: 13 growth stages; CONTAINS InterPro DOMAIN/s: Protein kinase, ATP binding site (InterPro:IPR017441), Protein kinase, catalytic domain (InterPro:IPR000719), Serine/threonine-protein kinase domain (InterPro:IPR002290), Serine/threonine-protein kinase-like domain (InterPro:IPR017442), Protein kinase-like domain (InterPro:IPR011009), Serine/threonine-protein kinase, active site (InterPro:IPR008271); BEST Arabidopsis thaliana protein match is: NIMA-related serine/threonine kinase 1 (TAIR:AT1G54510.3) |
| both | AT3G48750.1 | cell division control 2 | A-type cyclin-dependent kinase. Together with its specific inhibitor, the Kip-related protein, KRP2 they regulate the mitosis-to-endocycle transition during leaf development. Dominant negative mutations abolish cell division. Loss of function phenotype has reduced fertility with failure to transmit via pollen. Pollen development is arrested at the second mitotic division. Expression is regulated by environmental and chemical signals. Part of the promoter is responsible for expression in trichomes. Functions as a positive regulator of cell proliferation during development of the male gametophyte, embryo and endosperm. Phosphorylation of threonine 161 is required for activation of its associated kinase. | cell division control 2 (CDC2); FUNCTIONS IN: protein binding, protein kinase activity, cyclin-dependent protein kinase activity, kinase activity; INVOLVED IN: in 8 processes; LOCATED IN: in 6 components; EXPRESSED IN: 23 plant structures; EXPRESSED DURING: 13 growth stages; CONTAINS InterPro DOMAIN/s: Protein kinase, ATP binding site (InterPro:IPR017441), Protein kinase, catalytic domain (InterPro:IPR000719), Serine/threonine-protein kinase domain (InterPro:IPR002290), Serine/threonine-protein kinase-like domain (InterPro:IPR017442), Protein kinase-like domain (InterPro:IPR011009), Serine/threonine-protein kinase, active site (InterPro:IPR008271); BEST Arabidopsis thaliana protein match is: cyclin-dependent kinase B1;2 (TAIR:AT2G38620.2) |
| both | AT3G57230.1 | AGAMOUS-like 16 | MADS-box transcription factor. Expressed in leaf, root and stem, with higher RNA accumulation in guard cells and trichomes. AGL16 can directly interact with SVP and indirectly interact with FLC. Furthermore, the accumulation of AGL16 transcripts is modulated by miR824 (AT4G24415). The flowering time effect for the miR824/AGL16 module is more obvious in the Col-FRI background than in the Col-0 background. AGL16 controls flowering via a allelic dosage effect in long-day non-vernalized conditions. | AGAMOUS-like 16 (AGL16); FUNCTIONS IN: sequence-specific DNA binding transcription factor activity; INVOLVED IN: regulation of transcription, DNA-dependent, stomatal lineage progression; LOCATED IN: nucleus; EXPRESSED IN: 26 plant structures; EXPRESSED DURING: 13 growth stages; CONTAINS InterPro DOMAIN/s: Transcription factor, MADS-box (InterPro:IPR002100), Transcription factor, K-box (InterPro:IPR002487); BEST Arabidopsis thaliana protein match is: AGAMOUS-like 21 (TAIR:AT4G37940.1) |

| | | | | |
|---|---|---|---|---|
| both | AT4G04510.1 | cysteine-rich RLK (RECEPTOR-like protein kinase) 38 | Encodes a cysteine-rich receptor-like protein kinase. | cysteine-rich RLK (RECEPTOR-like protein kinase) 38 (CRK38); FUNCTIONS IN: kinase activity; INVOLVED IN: protein amino acid phosphorylation; LOCATED IN: endomembrane system; EXPRESSED IN: trichome, stamen; EXPRESSED DURING: 4 anthesis; CONTAINS InterPro DOMAIN/s: Protein kinase, ATP binding site (InterPro:IPR017441), Protein kinase, catalytic domain (InterPro:IPR000719), Protein of unknown function DUF26 (InterPro:IPR002902), Serine/threonine-protein kinase-like domain (InterPro:IPR017442), Protein kinase-like domain (InterPro:IPR011009), Serine/threonine-protein kinase, active site (InterPro:IPR008271); BEST Arabidopsis thaliana protein match is: cysteine-rich RLK (RECEPTOR-like protein kinase) 39 (TAIR:AT4G04540.1) |
| both | AT5G06700.1 | Plant protein of unknown function (DUF828) | Encodes a member of the TBL (TRICHOME BIREFRINGENCE-LIKE) gene family containing a plant-specific DUF231 (domain of unknown function) domain. TBL gene family has 46 members, two of which (TBR/AT5G06700 and TBL3/AT5G01360) have been shown to be involved in the synthesis and deposition of secondary wall cellulose, presumably by influencing the esterification state of pectic polymers. A nomenclature for this gene family has been proposed (Volker Bischoff & Wolf Scheible, 2010, personal communication). A tbr mutant is impaired in its ability to deposit secondary wall cellulose in specific cell types, most notably in trichomes. | FUNCTIONS IN: molecular_function unknown; INVOLVED IN: plant-type cell wall modification, pectin biosynthetic process, cellulose biosynthetic process, secondary cell wall biogenesis; LOCATED IN: cellular_component unknown; EXPRESSED IN: 23 plant structures; EXPRESSED DURING: 13 growth stages; CONTAINS InterPro DOMAIN/s: Protein of unknown function DUF231, plant (InterPro:IPR004253); BEST Arabidopsis thaliana protein match is: Plant protein of unknown function (DUF828) (TAIR:AT3G12060.1) |
| both | **AT5G40330.1** | myb domain protein 23 | Encodes a MYB gene that, when overexpressed ectopically, can induce ectopic trichome formation. It is a member of subgroup 15, together with WER and GL1. Members of this subgroup share a conserved motif of 19 amino acids in the putative transcription activation domain at the C-terminal end. The gene is expressed in leaves, stems, flowers, seeds and roots and quite strongly in trichomes. There is partial functional redundancy between ATMYB23 and GL1. The two proteins are functionally equivalent with respect to the regulation of trichome initiation but not with respect to trichome branching - which is controlled by MYB23 and not GL1. | myb domain protein 23 (MYB23); CONTAINS InterPro DOMAIN/s: SANT, DNA-binding (InterPro:IPR001005), Homeodomain-like (InterPro:IPR009057), Myb, DNA-binding (InterPro:IPR014778), HTH transcriptional regulator, Myb-type, DNA-binding (InterPro:IPR017930), Homeodomain-related (InterPro:IPR012287), Myb transcription factor (InterPro:IPR015495); BEST Arabidopsis thaliana protein match is: myb domain protein 0 (TAIR:AT3G27920.1) |

| | | | | |
|---|---|---|---|---|
| both | AT5G65530.1 | Protein kinase superfamily protein | Encodes a protein kinase involved in mediating resistance to fungi and also trichome branch number. Kinase activity is increased by ROP6 which also affects its sub-cellular localization (becomes localized to the cell periphery) | Protein kinase superfamily protein; FUNCTIONS IN: protein serine/threonine kinase activity, protein kinase activity, kinase activity, ATP binding; INVOLVED IN: protein amino acid phosphorylation; LOCATED IN: cellular_component unknown; EXPRESSED IN: 16 plant structures; EXPRESSED DURING: 8 growth stages; CONTAINS InterPro DOMAIN/s: Protein kinase, ATP binding site (InterPro:IPR017441), Serine/threonine-protein kinase domain (InterPro:IPR002290), Serine/threonine-protein kinase-like domain (InterPro:IPR017442), Serine/threonine-protein kinase, active site (InterPro:IPR008271), Protein kinase-like domain (InterPro:IPR011009), Protein kinase, catalytic domain (InterPro:IPR000719), Tyrosine-protein kinase, catalytic domain (InterPro:IPR020635); BEST Arabidopsis thaliana protein match is: ROP binding protein kinases 1 (TAIR:AT5G10520.1) |

(b) Flower petal

| Up-regulated in | Gene Name | Other Names | Description | Annotations |
|---|---|---|---|---|
| Mc | AT1G02065.1 | squamosa promoter binding protein-like 8 | Encodes an SBP-box gene, a member of the SPL gene family. Mutants are affected in micro- and megasporogenesis, trichome formation on sepals, and stamen filament elongation. | squamosa promoter binding protein-like 8 (SPL8); CONTAINS InterPro DOMAIN/s: Transcription factor, SBP-box (InterPro:IPR004333); BEST Arabidopsis thaliana protein match is: squamosa promoter binding protein-like 2 (TAIR:AT5G43270.1) |
| Mc | AT1G15570.1 | CYCLIN A2;3 | A2-type cyclin. Negatively regulates endocycles and acts as a key regulator of ploidy levels in Arabidopsis endoreduplication. Interacts physically with CDKA;1. Expressed preferentially in trichomes and young developing tissues. | CYCLIN A2;3 (CYCA2;3); CONTAINS InterPro DOMAIN/s: Cyclin, C-terminal (InterPro:IPR004367), Cyclin (InterPro:IPR006670), G2/mitotic-specific cyclin A (InterPro:IPR015453), Cyclin-like (InterPro:IPR011028), Cyclin-related (InterPro:IPR013763), Cyclin, N-terminal (InterPro:IPR006671), Cyclin, A/B/D/E (InterPro:IPR014400); BEST Arabidopsis thaliana protein match is: Cyclin A2;4 (TAIR:AT1G80370.1) |

| | | | | |
|---|---|---|---|---|
| Mc | **AT1G21970.1** | Histone superfamily protein | Transcriptional activator of genes required for both embryo maturation and cellular differentiation. Sequence is similar to HAP3 subunit of the CCAAT-box binding factor. HAP3 subunit is divided into three domains: an amino-terminal A domain, a central B domain, and a carboxyl-terminal C domain. LEC1 shared high similarity with other HAP3 homologs only in central, B domain. LEC1 is required for the specification of cotyledon identity and the completion of embryo maturation. It was sufficient to induce embryogenic programs in vegetative cells, suggesting that LEC1 is a major embryonic regulator that mediates the switch between embryo and vegetative development. Mutants are desiccation intolerant, have trichomes on cotyledons and exhibit precocious meristem activation. Levels of the ABI3 and FUS3 transcripts were significantly reduced in developing siliques of the lec1-1 mutants, indicating that LEC1 down-regulates FUS3 and ABI3.When LEC1 is overexpressed from an inducible promoter, the expression of numerous genes involved in fatty acid biosynthesis is increased suggesting a role in positive regulation of FA biosynthesis. | LEAFY COTYLEDON 1 (LEC1); FUNCTIONS IN: transcription activator activity, sequence-specific DNA binding transcription factor activity; INVOLVED IN: in 6 processes; LOCATED IN: nucleus, intracellular; EXPRESSED IN: seed, endosperm, cultured somatic embryo; EXPRESSED DURING: C globular stage, B proembryo stage, D bilateral stage, E expanded cotyledon stage; CONTAINS InterPro DOMAIN/s: Transcription factor, CBFA/NFYB, DNA topoisomerase (InterPro:IPR003957), Histone-fold (InterPro:IPR009072), Transcription factor CBF/NF-Y/archaeal histone (InterPro:IPR003958), Transcription factor, NFYB/HAP3, conserved site (InterPro:IPR003956); BEST Arabidopsis thaliana protein match is: nuclear factor Y, subunit B6 (TAIR:AT5G47670.2) |
| Mc | AT1G63650.3 | basic helix-loop-helix (bHLH) DNA-binding superfamily protein | Mutant has reduced trichomes, anthocyanin, and seed coat mucilage and abnormally patterned stomates. Mutants are defective in jasmonate-induced anthocyanin accumulation. Encodes a bHLH Transcription Factor 1. The protein is functionally redundant with GL3 and TT8 and interacts with TTG1, the myb proteins GL1, PAP1 and 2, CPC and TRY, and it will form heterodimers with GL3. Expression in N (non-hair cell forming) cell layers is negatively regulated by WER. Expression in H cells (hair cell forming) is promoted by CPC/TRY. | ENHANCER OF GLABRA 3 (EGL3); FUNCTIONS IN: DNA binding, sequence-specific DNA binding transcription factor activity; INVOLVED IN: epidermal cell fate specification, regulation of transcription; LOCATED IN: nucleus; EXPRESSED IN: 14 plant structures; EXPRESSED DURING: 7 growth stages; CONTAINS InterPro DOMAIN/s: Helix-loop-helix DNA-binding domain (InterPro:IPR001092), Helix-loop-helix DNA-binding (InterPro:IPR011598); BEST Arabidopsis thaliana protein match is: basic helix-loop-helix (bHLH) DNA-binding superfamily protein (TAIR:AT5G41315.1). |
| Mc | AT1G67030.1 | zinc finger protein 6 | Encodes a novel C2H2 zinc finger protein containing only a single zinc finger which plays a key role in regulating trichome development by integrating GA and cytokinin signaling. The mRNA is cell-to-cell mobile. | zinc finger protein 6 (ZFP6); FUNCTIONS IN: sequence-specific DNA binding transcription factor activity, zinc ion binding, nucleic acid binding; INVOLVED IN: regulation of transcription; LOCATED IN: cytosolic ribosome; EXPRESSED IN: 7 plant structures; EXPRESSED DURING: 4 anthesis, petal differentiation and expansion stage; CONTAINS InterPro DOMAIN/s: Zinc finger, C2H2-like (InterPro:IPR015880), Zinc finger, C2H2-type (InterPro:IPR007087); BEST Arabidopsis thaliana protein match is: C2H2 and C2HC zinc fingers superfamily protein (TAIR:AT1G68360.1) |

| | | | | |
|---|---|---|---|---|
| Mc | AT1G80350.1 | P-loop containing nucleoside triphosphate hydrolases superfamily protein | Encodes a p60 katanin protein that is expressed throughout the plant. Required for the specification of cell fates from early in development (in the meristem) through differentiation and for normal postmitotic organization of cortical microtubules into transverse arrays in root epidermis cells. Mutants display cytoskeletal defects. | ECTOPIC ROOT HAIR 3 (ERH3); FUNCTIONS IN: nucleoside-triphosphatase activity, nucleotide binding, ATP binding; INVOLVED IN: trichome branching, cortical microtubule organization, multidimensional cell growth, plant-type cell wall biogenesis, microtubule cytoskeleton organization; LOCATED IN: katanin complex; EXPRESSED IN: 23 plant structures; EXPRESSED DURING: 15 growth stages; CONTAINS InterPro DOMAIN/s: ATPase, AAA-type, core (InterPro:IPR003959), ATPase, AAA+ type, core (InterPro:IPR003593), ATPase, AAA-type, conserved site (InterPro:IPR003960), Vps4 oligomerisation, C-terminal (InterPro:IPR015415); BEST Arabidopsis thaliana protein match is: P-loop containing nucleoside triphosphate hydrolases superfamily protein (TAIR:AT2G34560.1) |
| Mc | AT2G30420.1 | Homeodomain-like superfamily protein | In a tandem repeat with AT2G30424 and AT2G30432 | ENHANCER OF TRY AND CPC 2 (ETC2); FUNCTIONS IN: DNA binding, sequence-specific DNA binding transcription factor activity; INVOLVED IN: trichome patterning, regulation of transcription; LOCATED IN: mitochondrion; CONTAINS InterPro DOMAIN/s: SANT, DNA-binding (InterPro:IPR001005), Myb, DNA-binding (InterPro:IPR014778), Homeodomain-related (InterPro:IPR012287), Myb transcription factor (InterPro:IPR015495); BEST Arabidopsis thaliana protein match is: Homeodomain-like superfamily protein (TAIR:AT2G30424.1) |
| Mc | AT2G41100.3 | Calcium-binding EF hand family protein | Encodes a calmodulin-like protein, with six potential calcium binding domains. Calcium binding shown by Ca(2+)-specific shift in electrophoretic mobility. Expression induced by touch and darkness. Expression may also be developmentally controlled. Expression in growing regions of roots, vascular tissue, root/shoot junctions, trichomes, branch points of the shoot, and regions of siliques and flowers. The mRNA is cell-to-cell mobile. | TOUCH 3 (TCH3); FUNCTIONS IN: calcium ion binding; INVOLVED IN: response to mechanical stimulus, response to temperature stimulus, thigmotropism, response to absence of light; LOCATED IN: plasma membrane; EXPRESSED IN: 26 plant structures; EXPRESSED DURING: 14 growth stages; CONTAINS InterPro DOMAIN/s: EF-Hand 1, calcium-binding site (InterPro:IPR018247), EF-HAND 2 (InterPro:IPR018249), EF-hand-like domain (InterPro:IPR011992), Calcium-binding EF-hand (InterPro:IPR002048), EF-hand (InterPro:IPR018248); BEST Arabidopsis thaliana protein match is: calmodulin 5 (TAIR:AT2G27030.3) |

| | | | | |
|---|---|---|---|---|
| Mc | AT3G13530.1 | mitogen-activated protein kinase kinase kinase 7 | MAP3K epsilon protein kinase 1 is functionally redundant with MAP3Ke2. Required for pollen development but not essential. map3ke1;map3ke2 double-mutant pollen grains develop plasma membrane irregularities following pollen mitosis I. Localized primarily in the plasma membrane. Expressed in leaf trichomes, root columella cells and developing ovules. | mitogen-activated protein kinase kinase kinase 7 (MAPKKK7); FUNCTIONS IN: protein serine/threonine kinase activity, binding, protein kinase activity, kinase activity, ATP binding; INVOLVED IN: plasma membrane organization, pollen development; LOCATED IN: cytosol, plasma membrane; EXPRESSED IN: 6 plant structures; EXPRESSED DURING: ovule developmental stages; CONTAINS InterPro DOMAIN/s: Protein kinase, ATP binding site (InterPro:IPR017441), Serine/threonine-protein kinase domain (InterPro:IPR002290), Armadillo-like helical (InterPro:IPR011989), Armadillo (InterPro:IPR000225), Serine/threonine-protein kinase-like domain (InterPro:IPR017442), Protein kinase-like domain (InterPro:IPR011009), Serine/threonine-protein kinase, active site (InterPro:IPR008271), Protein kinase, catalytic domain (InterPro:IPR000719), Armadillo-type fold (InterPro:IPR016024); BEST Arabidopsis thaliana protein match is: mitogen-activated protein kinase kinase kinase 6 (TAIR:AT3G07980.1) |
| Mc | **AT3G55360.1** | 3-oxo-5-alpha-steroid 4-dehydrogenase family protein | Enoyl-CoA reductase is involved in all very long chain fatty acids (VLCFA) elongation reactions that are required for cuticular wax, storage lipid and sphingolipid metabolism. The protein is located in the ER, but in contrast to its yeast homolog TSC13 is not particularly enriched in the nuclear envelope-vacuole junction. Mutants in this gene show abnormal organ morphology and stem glossiness. Cells in all tissues are only about 1/3 of the size of wild type cells. The morphological changes are most likely to result from the reduction in the VLCFA content of sphingolipids. Mutants also show abnormalities in the endocytic membrane organization and transport as well as reduced trichome papillae. | ECERIFERUM 10 (CER10); FUNCTIONS IN: fatty acid elongase activity, trans-2-enoyl-CoA reductase (NADPH) activity, 3-oxo-5-alpha-steroid 4-dehydrogenase activity; INVOLVED IN: sphingolipid metabolic process, wax biosynthetic process; LOCATED IN: endoplasmic reticulum, fatty acid elongase complex, plasma membrane, endoplasmic reticulum membrane; EXPRESSED IN: 24 plant structures; EXPRESSED DURING: 13 growth stages; CONTAINS InterPro DOMAIN/s: 3-oxo-5-alpha-steroid 4-dehydrogenase, C-terminal (InterPro:IPR001104); BEST Arabidopsis thaliana protein match is: 3-oxo-5-alpha-steroid 4-dehydrogenase family protein (TAIR:AT5G16010.1) |
| Mc | **AT4G20780.1** | calmodulin like 42 | Calcium sensor involved in trichome branching. | calmodulin like 42 (CML42); FUNCTIONS IN: calcium ion binding; INVOLVED IN: trichome branching; LOCATED IN: cellular_component unknown; EXPRESSED IN: 23 plant structures; EXPRESSED DURING: 13 growth stages; CONTAINS InterPro DOMAIN/s: EF-Hand 1, calcium-binding site (InterPro:IPR018247), EF-HAND 2 (InterPro:IPR018249), EF-hand-like domain (InterPro:IPR011992), Calcium-binding EF-hand (InterPro:IPR002048), EF-hand (InterPro:IPR018248); BEST Arabidopsis thaliana protein match is: calmodulin like 43 (TAIR:AT5G44460.1) |

| | | | | |
|---|---|---|---|---|
| Mc | AT5G28646.2 | TPX2 (targeting protein for Xklp2) protein family | Encodes a novel protein. The wvd2 gain-of-function mutant has impaired cell expansion and root waving, and changed root skewing. | WAVE-DAMPENED 2 (WVD2); FUNCTIONS IN: molecular_function unknown; INVOLVED IN: trichome branching, multidimensional cell growth, microtubule bundle formation, establishment or maintenance of cell polarity, microtubule cytoskeleton organization; LOCATED IN: cortical microtubule; CONTAINS InterPro DOMAIN/s: Xklp2 targeting protein (InterPro:IPR009675); BEST Arabidopsis thaliana protein match is: WVD2-like 1 (TAIR:AT3G04630.3) |
| Ms | **AT1G56580.1** | Protein of unknown function, DUF538 | Encodes SMALLER WITH VARIABLE BRANCHES (SVB), a protein with a conserved domain of unknown function (DUF538). The trichomes of the SVB mutants are smaller and exhibit branches of variable length and number. | SMALLER WITH VARIABLE BRANCHES (SVB); CONTAINS InterPro DOMAIN/s: Protein of unknown function DUF538 (InterPro:IPR007493); BEST Arabidopsis thaliana protein match is: Protein of unknown function, DUF538 (TAIR:AT1G09310.1) |
| Ms | AT3G09790.1 | ubiquitin 8 | Encodes a ubiquitin-like protein that contains tandem repeats of the ubiquitin coding region, but at least one repeat per gene encodes a protein with amino acid substitutions. | ubiquitin 8 (UBQ8); INVOLVED IN: protein modification process, ubiquitin-dependent protein catabolic process; LOCATED IN: cell wall, intracellular, vacuole; EXPRESSED IN: guard cell, juvenile leaf, trichome; CONTAINS InterPro DOMAIN/s: Ubiquitin subgroup (InterPro:IPR019956), Ubiquitin (InterPro:IPR000626), Ubiquitin supergroup (InterPro:IPR019955); BEST Arabidopsis thaliana protein match is: polyubiquitin 10 (TAIR:AT4G05320.4) |
| Ms | AT3G45290.1 | Seven transmembrane MLO family protein | A member of a large family of seven-transmembrane domain proteins specific to plants, homologs of the barley mildew resistance locus o (MLO) protein. The Arabidopsis genome contains 15 genes encoding MLO proteins, with localization in plasma membrane. Phylogenetic analysis revealed four clades of closely-related AtMLO genes. ATMLO3 belongs to the clade IV, with AtMLO2, AtMLO6 and AtMLO12. The gene is expressed during early seedling growth, in primary root and lateral root primordia, in fruit abscission zone, in vascular system of cotyledons and in trichomes of young leaves,; it was not expressed in mature rosette leaves, as shown by GUS activity patterns. The expression of several phylogenetically closely-related AtMLO genes showed similar or overlapping tissue specificity and analogous responsiveness to external stimuli, suggesting functional redundancy, co-function, or antagonistic function(s). | MILDEW RESISTANCE LOCUS O 3 (MLO3); FUNCTIONS IN: calmodulin binding; INVOLVED IN: cell death, defense response; LOCATED IN: integral to membrane, plasma membrane; EXPRESSED IN: 29 plant structures; EXPRESSED DURING: 14 growth stages; CONTAINS InterPro DOMAIN/s: Mlo-related protein (InterPro:IPR004326); BEST Arabidopsis thaliana protein match is: Seven transmembrane MLO family protein (TAIR:AT2G39200.1) |

| | | | | |
|---|---|---|---|---|
| Ms | AT4G32410.1 | cellulose synthase 1 | Encodes a cellulose synthase isomer. CESA1 mutants have cellulose defect in the primary cell wall. Multiple lines of evidence suggest that CESA1, along with CESA3 and CESA6 are present in the same plasma membrane complex for cellulose biosynthesis. lasma membrane complex for cellulose biosynthesis. As inferred from the null role of secondary wall-type CesAs, included in a set of five primary wall-type CesAs that may support trichome cell wall thickening. | cellulose synthase 1 (CESA1); FUNCTIONS IN: cellulose synthase activity, transferase activity, transferring glycosyl groups; INVOLVED IN: primary cell wall biogenesis, cellulose biosynthetic process, plant-type cell wall biogenesis, hyperosmotic salinity response; LOCATED IN: Golgi apparatus, plasma membrane; EXPRESSED IN: 26 plant structures; EXPRESSED DURING: 16 growth stages; CONTAINS InterPro DOMAIN/s: Cellulose synthase (InterPro:IPR005150), Zinc finger, RING-type (InterPro:IPR001841); BEST Arabidopsis thaliana protein match is: cellulose synthase 10 (TAIR:AT2G25540.1) |
| Ms | AT5G06700.1 | Plant protein of unknown function (DUF828) | Encodes a member of the TBL (TRICHOME BIREFRINGENCE-LIKE) gene family containing a plant-specific DUF231 (domain of unknown function) domain. TBL gene family has 46 members, two of which (TBR/AT5G06700 and TBL3/AT5G01360) have been shown to be involved in the synthesis and deposition of secondary wall cellulose, presumably by influencing the esterification state of pectic polymers. A nomenclature for this gene family has been proposed (Volker Bischoff & Wolf Scheible, 2010, personal communication). A tbr mutant is impaired in its ability to deposit secondary wall cellulose in specific cell types, most notably in trichomes. | FUNCTIONS IN: molecular_function unknown; INVOLVED IN: plant-type cell wall modification, pectin biosynthetic process, cellulose biosynthetic process, secondary cell wall biogenesis; LOCATED IN: cellular_component unknown; EXPRESSED IN: 23 plant structures; EXPRESSED DURING: 13 growth stages; CONTAINS InterPro DOMAIN/s: Protein of unknown function DUF231, plant (InterPro:IPR004253); BEST Arabidopsis thaliana protein match is: Plant protein of unknown function (DUF828) (TAIR:AT3G12060.1) |
| Ms | **AT5G23940.1** | HXXXD-type acyl-transferase family protein | Encodes PERMEABLE LEAVES3 (PEL3), a putative acyl-transferase. Mutation in this locus results in altered trichome phenotype (trcichomes become tangled during leaf expansion). Additional phenotype includes altered cuticle layer. | PERMEABLE LEAVES3 (PEL3); FUNCTIONS IN: transferase activity, transferring acyl groups other than amino-acyl groups, transferase activity; INVOLVED IN: epidermis morphogenesis, localization, trichome morphogenesis, cutin biosynthetic process, embryo development ending in seed dormancy; LOCATED IN: cytoplasm; EXPRESSED IN: 21 plant structures; EXPRESSED DURING: 7 growth stages; CONTAINS InterPro DOMAIN/s: Transferase (InterPro:IPR003480); BEST Arabidopsis thaliana protein match is: HXXXD-type acyl-transferase family protein (TAIR:AT2G39980.1) |

| | | | | |
|---|---|---|---|---|
| Ms | AT5G37770.1 | EF hand calcium-binding protein family | Encodes a protein with 40% similarity to calmodulin. Binds Ca(2+) and, as a consequence, undergoes conformational changes. CML24 expression occurs in all major organs, and transcript levels are increased from 2- to 15-fold in plants subjected to touch, darkness, heat, cold, hydrogen peroxide, abscisic acid (ABA), and indole-3-acetic acid. However, CML24 protein accumulation changes were not detectable. The putative CML24 regulatory region confers reporter expression at sites of predicted mechanical stress; in regions undergoing growth; in vascular tissues and various floral organs; and in stomata, trichomes, and hydathodes. CML24-underexpressing transgenics are resistant to ABA inhibition of germination and seedling growth, are defective in long-day induction of flowering, and have enhanced tolerance to CoCl(2), molybdic acid, ZnSO(4), and MgCl(2). Also regulates nitric oxide levels. | TOUCH 2 (TCH2); FUNCTIONS IN: calcium ion binding; INVOLVED IN: in 13 processes; LOCATED IN: plasma membrane, actin cytoskeleton; EXPRESSED IN: 34 plant structures; EXPRESSED DURING: 14 growth stages; CONTAINS InterPro DOMAIN/s: EF-Hand 1, calcium-binding site (InterPro:IPR018247), EF-HAND 2 (InterPro:IPR018249), EF-hand-like domain (InterPro:IPR011992), Calcium-binding EF-hand (InterPro:IPR002048), EF-hand (InterPro:IPR018248); BEST Arabidopsis thaliana protein match is: calmodulin like 23 (TAIR:AT1G66400.1) |
| Ms | **AT5G64740.1** | cellulose synthase 6 | Encodes a cellulose synthase isomer. CESA6 mutants have cellulose defect in the primary cell wall. Multiple lines of evidence suggest that CESA6, along with CESA1 and CESA3 are present in the same plasma membrane complex for cellulose biosynthesis. CESA2 and CESA5 are related to CESA6, having partially redundant roles. As inferred from the null role of secondary wall-type CesAs, included in a set of five primary wall-type CesAs that may support trichome cell wall thickening. The mRNA is cell-to-cell mobile. | cellulose synthase 6 (CESA6); FUNCTIONS IN: cellulose synthase activity, transferase activity, transferring glycosyl groups; INVOLVED IN: in 6 processes; LOCATED IN: cortical microtubule, transverse to long axis, Golgi apparatus, cellulose synthase complex, plasma membrane, membrane; EXPRESSED IN: 25 plant structures; EXPRESSED DURING: 15 growth stages; CONTAINS InterPro DOMAIN/s: Cellulose synthase (InterPro:IPR005150), Zinc finger, RING-type (InterPro:IPR001841); BEST Arabidopsis thaliana protein match is: cellulose synthase 5 (TAIR:AT5G09870.1) |
| both | AT1G21210.1 | wall associated kinase 4 | Cell wall-associated ser/thr kinase involved in cell elongation and lateral root development | wall associated kinase 4 (WAK4); FUNCTIONS IN: protein serine/threonine kinase activity, protein kinase activity, ATP binding, calcium ion binding; INVOLVED IN: protein amino acid phosphorylation, unidimensional cell growth, lateral root development; LOCATED IN: plasma membrane; EXPRESSED IN: root, trichome; CONTAINS InterPro DOMAIN/s: EGF-like calcium-binding (InterPro:IPR001881), EGF-like, type 3 (InterPro:IPR000742), Serine-threonine/tyrosine-protein kinase (InterPro:IPR001245), Protein kinase-like domain (InterPro:IPR011009), Serine/threonine-protein kinase, active site (InterPro:IPR008271), Protein kinase, catalytic domain (InterPro:IPR000719), EGF-like calcium-binding, conserved site (InterPro:IPR018097), EGF-type aspartate/asparagine hydroxylation site (InterPro:IPR000152), EGF calcium-binding (InterPro:IPR013091), EGF-like (InterPro:IPR006210); BEST Arabidopsis thaliana protein match is: wall-associated kinase 2 (TAIR:AT1G21270.1) |

| | | | | |
|---|---|---|---|---|
| both | **AT1G33240.1** | GT-2-like 1 | Encodes a plant transcriptional activator that contains two separate, but similar, trihelix DNA-binding domains, similar to GT-2. Gene is expressed in all aerial parts of the plant, with higher level of expression in siliques. At-GTL2 was thought to be a duplicated copy of this gene but is likely to be a cloning artefact, the result of a chimeric clone. Regulates ploidy-dependent cell growth in trichome. | GT-2-like 1 (GTL1); CONTAINS InterPro DOMAIN/s: SANT, DNA-binding (InterPro:IPR001005), MYB-like (InterPro:IPR017877); BEST Arabidopsis thaliana protein match is: Duplicated homeodomain-like superfamily protein (TAIR:AT1G76890.2) |
| both | AT1G64670.1 | alpha/beta-Hydrolases superfamily protein | Encodes a epidermally expressed extracellular protein that likely functions as an alpha-beta hydrolase and is required for normal cuticle formation. Homozygous mutant plants are dwarfed and have abnormal leaves, collapsed cells, reduced numbers of trichomes. The specific role of BDG is unclear: it may function in cutin biosynthesis or as a cross-linking enzyme in the cell wall itself. | BODYGUARD1 (BDG1); CONTAINS InterPro DOMAIN/s: Alpha/beta hydrolase fold-1 (InterPro:IPR000073); BEST Arabidopsis thaliana protein match is: alpha/beta-Hydrolases superfamily protein (TAIR:AT5G41900.1) |
| both | AT3G01140.1 | myb domain protein 106 | Encodes a MIXTA-like MYB gene NOECK (NOK). Loss of function mutations show an increased number of branchpoints in leaf trichomes suggesting a role in negative regulation of trichome branching. | myb domain protein 106 (MYB106); FUNCTIONS IN: DNA binding, sequence-specific DNA binding transcription factor activity; INVOLVED IN: trichome branching, regulation of transcription, DNA-dependent; EXPRESSED IN: 18 plant structures; EXPRESSED DURING: 7 growth stages; CONTAINS InterPro DOMAIN/s: SANT, DNA-binding (InterPro:IPR001005), Homeodomain-like (InterPro:IPR009057), Myb, DNA-binding (InterPro:IPR014778), Myb transcription factor (InterPro:IPR015495), Homeodomain-related (InterPro:IPR012287), HTH transcriptional regulator, Myb-type, DNA-binding (InterPro:IPR017930); BEST Arabidopsis thaliana protein match is: myb domain protein 16 (TAIR:AT5G15310.1) |
| both | AT3G13540.1 | myb domain protein 5 | Encodes a member of the MYB family of transcriptional regulators. MYB5 act as a negative regulator of trichome branching and play a role in the correct formation of the seed coat and possibly the formation the underlying endosperm layers. Loss of function mutations have defects in seed coat mucilage and columella cells as well as trichome defects (smaller and reduced number of branches). | myb domain protein 5 (MYB5); CONTAINS InterPro DOMAIN/s: SANT, DNA-binding (InterPro:IPR001005), Homeodomain-like (InterPro:IPR009057), Myb, DNA-binding (InterPro:IPR014778), HTH transcriptional regulator, Myb-type, DNA-binding (InterPro:IPR017930), Homeodomain-related (InterPro:IPR012287), Myb transcription factor (InterPro:IPR015495); BEST Arabidopsis thaliana protein match is: myb domain protein 17 (TAIR:AT3G61250.1) |
| both | AT3G21560.1 | UDP-Glycosyltransferase superfamily protein | Encodes a protein with sinapic acid:UDP-glucose glucosyltransferase activity. Mutants defective in this gene are hyper-fluorescent (which accumulate in their trichomes a compound that is likely to be 3',5'-dimethoxynaringenin chalcone or sinapoyltriacetic acid lactone, potential products of the concerted action of 4-coumarate CoA ligase and chalcone synthase on sinapic acid). Also shown to be required for Arabidopsis nonhost resistance to the Asian soybean rust pathogen Phakopsora pachyrhizi. | UGT84A2; CONTAINS InterPro DOMAIN/s: UDP-glucuronosyl/UDP-glucosyltransferase (InterPro:IPR002213); BEST Arabidopsis thaliana protein match is: UDP-Glycosyltransferase superfamily protein (TAIR:AT4G15490.1) |

| | | | | |
|---|---|---|---|---|
| both | AT3G48750.1 | cell division control 2 | A-type cyclin-dependent kinase. Together with its specific inhibitor, the Kip-related protein, KRP2 they regulate the mitosis-to-endocycle transition during leaf development. Dominant negative mutations abolish cell division. Loss of function phenotype has reduced fertility with failure to transmit via pollen. Pollen development is arrested at the second mitotic division. Expression is regulated by environmental and chemical signals. Part of the promoter is responsible for expression in trichomes. Functions as a positive regulator of cell proliferation during development of the male gametophyte, embryo and endosperm. Phosphorylation of threonine 161 is required for activation of its associated kinase. | cell division control 2 (CDC2); FUNCTIONS IN: protein binding, protein kinase activity, cyclin-dependent protein kinase activity, kinase activity; INVOLVED IN: in 8 processes; LOCATED IN: in 6 components; EXPRESSED IN: 23 plant structures; EXPRESSED DURING: 13 growth stages; CONTAINS InterPro DOMAIN/s: Protein kinase, ATP binding site (InterPro:IPR017441), Protein kinase, catalytic domain (InterPro:IPR000719), Serine/threonine-protein kinase domain (InterPro:IPR002290), Serine/threonine-protein kinase-like domain (InterPro:IPR017442), Protein kinase-like domain (InterPro:IPR011009), Serine/threonine-protein kinase, active site (InterPro:IPR008271); BEST Arabidopsis thaliana protein match is: cyclin-dependent kinase B1;2 (TAIR:AT2G38620.2) |
| both | AT3G57230.1 | AGAMOUS-like 16 | MADS-box transcription factor. Expressed in leaf, root and stem, with higher RNA accumulation in guard cells and trichomes. AGL16 can directly interact with SVP and indirectly interact with FLC. Furthermore, the accumulation of AGL16 transcripts is modulated by miR824 (AT4G24415). The flowering time effect for the miR824/AGL16 module is more obvious in the Col-FRI background than in the Col-0 background. AGL16 controls flowering via a allelic dosage effect in long-day non-vernalized conditions. | AGAMOUS-like 16 (AGL16); FUNCTIONS IN: sequence-specific DNA binding transcription factor activity; INVOLVED IN: regulation of transcription, DNA-dependent, stomatal lineage progression; LOCATED IN: nucleus; EXPRESSED IN: 26 plant structures; EXPRESSED DURING: 13 growth stages; CONTAINS InterPro DOMAIN/s: Transcription factor, MADS-box (InterPro:IPR002100), Transcription factor, K-box (InterPro:IPR002487); BEST Arabidopsis thaliana protein match is: AGAMOUS-like 21 (TAIR:AT4G37940.1) |
| both | AT4G04510.1 | cysteine-rich RLK (RECEPTOR-like protein kinase) 38 | Encodes a cysteine-rich receptor-like protein kinase. | cysteine-rich RLK (RECEPTOR-like protein kinase) 38 (CRK38); FUNCTIONS IN: kinase activity; INVOLVED IN: protein amino acid phosphorylation; LOCATED IN: endomembrane system; EXPRESSED IN: trichome, stamen; EXPRESSED DURING: 4 anthesis; CONTAINS InterPro DOMAIN/s: Protein kinase, ATP binding site (InterPro:IPR017441), Protein kinase, catalytic domain (InterPro:IPR000719), Protein of unknown function DUF26 (InterPro:IPR002902), Serine/threonine-protein kinase-like domain (InterPro:IPR017442), Protein kinase-like domain (InterPro:IPR011009), Serine/threonine-protein kinase, active site (InterPro:IPR008271); BEST Arabidopsis thaliana protein match is: cysteine-rich RLK (RECEPTOR-like protein kinase) 39 (TAIR:AT4G04540.1) |

| | | | | |
|---|---|---|---|---|
| both | AT5G05170.1 | Cellulose synthase family protein | Encodes a cellulose synthase isomer. CESA3 mutants have cellulose defect in the primary cell wall. Multiple lines of evidence suggest that CESA3, along with CESA1 and CESA6 are present in the same plasma membrane complex for cellulose biosynthesis. As inferred from the null role of secondary wall-type CesAs, included in a set of five primary wall-type CesAs that may support trichome cell wall thickening. The xylem cells in primary root have reduced cell expansion and higher than normal lignification. | CONSTITUTIVE EXPRESSION OF VSP 1 (CEV1); FUNCTIONS IN: cellulose synthase activity, transferase activity, transferring glycosyl groups; INVOLVED IN: primary cell wall biogenesis, cellulose biosynthetic process, defense response; LOCATED IN: Golgi apparatus, plasma membrane; EXPRESSED IN: 27 plant structures; EXPRESSED DURING: 14 growth stages; CONTAINS InterPro DOMAIN/s: Cellulose synthase (InterPro:IPR005150), Zinc finger, RING-type (InterPro:IPR001841); BEST Arabidopsis thaliana protein match is: cellulose synthase 1 (TAIR:AT4G32410.1) |
| both | AT5G65530.1 | Protein kinase superfamily protein | Encodes a protein kinase involved in mediating resistance to fungi and also trichome branch number. Kinase activity is increased by ROP6 which also affects its sub-cellular localization (becomes localized to the cell periphery) | Protein kinase superfamily protein; FUNCTIONS IN: protein serine/threonine kinase activity, protein kinase activity, kinase activity, ATP binding; INVOLVED IN: protein amino acid phosphorylation; LOCATED IN: cellular_component unknown; EXPRESSED IN: 16 plant structures; EXPRESSED DURING: 8 growth stages; CONTAINS InterPro DOMAIN/s: Protein kinase, ATP binding site (InterPro:IPR017441), Serine/threonine-protein kinase domain (InterPro:IPR002290), Serine/threonine-protein kinase-like domain (InterPro:IPR017442), Serine/threonine-protein kinase, active site (InterPro:IPR008271), Protein kinase-like domain (InterPro:IPR011009), Protein kinase, catalytic domain (InterPro:IPR000719), Tyrosine-protein kinase, catalytic domain (InterPro:IPR020635); BEST Arabidopsis thaliana protein match is: ROP binding protein kinases 1 (TAIR:AT5G10520.1) |

**Table S6** Annotation of transgressively segregated transcripts in the F1 hybrid leaf tissue.

(a) Up-regulated

| Gene Name | Other Names | Description | Annotations |
|---|---|---|---|
| AT1G22400.1 | UDP-Glycosyltransferase superfamily protein | NULL | UGT85A1; FUNCTIONS IN: in 6 functions; INVOLVED IN: metabolic process; LOCATED IN: cellular_component unknown; EXPRESSED IN: 22 plant structures; EXPRESSED DURING: 10 growth stages; CONTAINS InterPro DOMAIN/s: UDP-glucuronosyl/UDP-glucosyltransferase (InterPro:IPR002213); BEST Arabidopsis thaliana protein match is: UDP-glucosyl transferase 85A3 (TAIR:AT1G22380.1) |
| AT1G50020.1 | NULL | NULL | unknown protein; FUNCTIONS IN: molecular_function unknown; INVOLVED IN: biological_process unknown; LOCATED IN: chloroplast thylakoid membrane, chloroplast; EXPRESSED IN: 24 plant structures; EXPRESSED DURING: 15 growth stages |
| AT1G54740.1 | Protein of unknown function (DUF3049) | NULL | Protein of unknown function (DUF3049); CONTAINS InterPro DOMAIN/s: Protein of unknown function DUF3049 (InterPro:IPR021410); BEST Arabidopsis thaliana protein match is: Protein of unknown function (DUF3049) (TAIR:AT5G22390.1) |
| AT1G65870.1 | Disease resistance-responsive (dirigent-like protein) family protein | NULL | Disease resistance-responsive (dirigent-like protein) family protein; FUNCTIONS IN: molecular_function unknown; INVOLVED IN: lignan biosynthetic process, defense response; LOCATED IN: cell wall; EXPRESSED IN: 13 plant structures; EXPRESSED DURING: 9 growth stages; CONTAINS InterPro DOMAIN/s: Plant disease resistance response protein (InterPro:IPR004265); BEST Arabidopsis thaliana protein match is: Disease resistance-responsive (dirigent-like protein) family protein (TAIR:AT1G22900.1) |
| AT1G70830.5 | MLP-like protein 28 | NULL | MLP-like protein 28 (MLP28); FUNCTIONS IN: molecular_function unknown; INVOLVED IN: response to biotic stimulus, defense response; LOCATED IN: cellular_component unknown; EXPRESSED IN: 22 plant structures; EXPRESSED DURING: 14 growth stages; CONTAINS InterPro DOMAIN/s: Bet v I allergen (InterPro:IPR000916); BEST Arabidopsis thaliana protein match is: MLP-like protein 34 (TAIR:AT1G70850.3) |

| | | | |
|---|---|---|---|
| AT1G73010.1 | phosphate starvation-induced gene 2 | Encodes PPsPase1, a pyrophosphate-specific phosphatase catalyzing the specific cleavage of pyrophosphate (Km 38.8 uM) with an alkaline catalytic pH optimum. Expression is upregulated in the shoot of cax1/cax3 mutant. | phosphate starvation-induced gene 2 (PS2); FUNCTIONS IN: phosphatase activity; INVOLVED IN: metabolic process; LOCATED IN: cellular_component unknown; EXPRESSED IN: 19 plant structures; EXPRESSED DURING: 10 growth stages; CONTAINS InterPro DOMAIN/s: Pyridoxal phosphate phosphatase, PHOSPHO2 (InterPro:IPR016965), HAD-superfamily hydrolase, subfamily IB, PSPase-like (InterPro:IPR006383), Pyridoxal phosphate phosphatase-related (InterPro:IPR006384); BEST Arabidopsis thaliana protein match is: Pyridoxal phosphate phosphatase-related protein (TAIR:AT1G17710.1) |
| AT1G75580.1 | SAUR-like auxin-responsive protein family | NULL | SAUR-like auxin-responsive protein family ; CONTAINS InterPro DOMAIN/s: Auxin responsive SAUR protein (InterPro:IPR003676); BEST Arabidopsis thaliana protein match is: SAUR-like auxin-responsive protein family (TAIR:AT1G19830.1) |
| AT2G33420.1 | Protein of unknown function (DUF810) | NULL | CONTAINS InterPro DOMAIN/s: Munc13 homology 1 (InterPro:IPR014770), Protein of unknown function DUF810 (InterPro:IPR008528), Mammalian uncoordinated homology 13, domain 2 (InterPro:IPR014772); BEST Arabidopsis thaliana protein match is: Protein of unknown function (DUF810) (TAIR:AT1G04470.1) |
| AT2G36830.1 | gamma tonoplast intrinsic protein | Encodes a tonoplast intrinsic protein, which functions as water channel. It has also been shown to be able to facilitate the transport of urea and hydrogen peroxide. Highly expressed in vascular tissues of the root, stem, cauline leaves and flowers but not in the apical meristems. The mRNA is cell-to-cell mobile. | gamma tonoplast intrinsic protein (GAMMA-TIP); FUNCTIONS IN: water channel activity, urea transmembrane transporter activity; INVOLVED IN: in 6 processes; LOCATED IN: in 7 components; EXPRESSED IN: 33 plant structures; EXPRESSED DURING: 13 growth stages; CONTAINS InterPro DOMAIN/s: Major intrinsic protein, conserved site (InterPro:IPR022357), Aquaporin (InterPro:IPR012269), Major intrinsic protein (InterPro:IPR000425); BEST Arabidopsis thaliana protein match is: tonoplast intrinsic protein 2 (TAIR:AT3G26520.1) |
| AT3G24520.1 | heat shock transcription factor C1 | Member of Heat Stress Transcription Factor (Hsf) family | heat shock transcription factor C1 (HSFC1); FUNCTIONS IN: DNA binding, sequence-specific DNA binding transcription factor activity; INVOLVED IN: regulation of transcription, DNA-dependent; LOCATED IN: nucleus; EXPRESSED IN: 14 plant structures; EXPRESSED DURING: LP.06 six leaves visible, LP.04 four leaves visible, 4 anthesis, petal differentiation and expansion stage, LP.08 eight leaves visible; CONTAINS InterPro DOMAIN/s: Winged helix-turn-helix transcription repressor DNA-binding (InterPro:IPR011991), Interferon induced 35kDa, N-terminal (InterPro:IPR009938), Heat shock factor (HSF)-type, DNA-binding (InterPro:IPR000232); BEST Arabidopsis thaliana protein match is: heat shock factor 1 (TAIR:AT4G17750.1) |

| | | | |
|---|---|---|---|
| AT4G16740.1 | terpene synthase 03 | Encodes an (E,E)-alpha-farnesene synthase in the Col ecotype of Arabidopsis. This enzyme can also catalyze the formation of (E)-beta-ocimene as well as trace amounts of myrcene and other related compounds in vitro. The cytosolic localization of the protein may make it favor (E,E)-alpha-farnesene biosynthesis because the precursor of this product, FPP, is primarily cytosolic. Transcript levels for this gene increase in response to treatment with the jasmonic acid mimic coronalon or in response to the insect Plutella xylostella. TPS03 transcripts can also be detected in flowers. A similar protein from the C24 ecotype with one amino acid change (S267F) has a different substrate specificity. | terpene synthase 03 (TPS03); CONTAINS InterPro DOMAIN/s: Terpene synthase, metal-binding domain (InterPro:IPR005630), Terpenoid synthase (InterPro:IPR008949), Terpenoid cylases/protein prenyltransferase alpha-alpha toroid (InterPro:IPR008930), Terpene synthase-like (InterPro:IPR001906); BEST Arabidopsis thaliana protein match is: terpene synthase-like sequence-1,8-cineole (TAIR:AT3G25820.1) |
| AT4G18700.1 | CBL-interacting protein kinase 12 | Encodes CBL-interacting protein kinase 12 (CIPK12). | CBL-interacting protein kinase 12 (CIPK12); FUNCTIONS IN: protein serine/threonine kinase activity, protein kinase activity, kinase activity, ATP binding; INVOLVED IN: signal transduction, protein amino acid phosphorylation; EXPRESSED IN: 24 plant structures; EXPRESSED DURING: 15 growth stages; CONTAINS InterPro DOMAIN/s: Protein kinase, ATP binding site (InterPro:IPR017441), Serine/threonine-protein kinase domain (InterPro:IPR002290), NAF/FISL domain (InterPro:IPR018451), Serine/threonine-protein kinase-like domain (InterPro:IPR017442), Protein kinase-like domain (InterPro:IPR011009), Serine/threonine-protein kinase, active site (InterPro:IPR008271), NAF domain (InterPro:IPR004041), CBL-interacting protein kinase (InterPro:IPR020660), Protein kinase, catalytic domain (InterPro:IPR000719), Calcium/calmodulin-dependent protein kinase-like (InterPro:IPR020636); BEST Arabidopsis thaliana protein match is: CBL-interacting protein kinase 19 (TAIR:AT5G45810.1) |
| AT4G23496.1 | SPIRAL1-like5 | Belongs to a six-member gene family in Arabidopsis; all members share high sequence similarity in amino- and carboxy-terminal regions. Regulates cortical microtubule organization. Mutant plants exhibit altered patterns of root, leaf and petal growth as a result of defective anisotropic cell expansion. | SPIRAL1-like5 (SP1L5); BEST Arabidopsis thaliana protein match is: SPIRAL1-like2 (TAIR:AT1G69230.2) |

| | | | |
|---|---|---|---|
| AT5G06530.1 | ABC-2 type transporter family protein | Encodes ABCG22, an ABC transporter gene. Mutation results in increased water transpiration and drought susceptibility. | ABC-2 type transporter family protein; FUNCTIONS IN: ATPase activity, coupled to transmembrane movement of substances; LOCATED IN: plasma membrane; EXPRESSED IN: 23 plant structures; EXPRESSED DURING: 13 growth stages; CONTAINS InterPro DOMAIN/s: ATPase, AAA+ type, core (InterPro:IPR003593), ABC transporter-like (InterPro:IPR003439), ABC-2 type transporter (InterPro:IPR013525), ABC transporter, conserved site (InterPro:IPR017871); BEST Arabidopsis thaliana protein match is: ABC-2 type transporter family protein (TAIR:AT3G52310.1) |

(b) Down-regulated

| Gene Name | Other Names | Description | Annotations |
|---|---|---|---|
| AT1G48750.1 | Bifunctional inhibitor/lipid-transfer protein/seed storage 2S albumin superfamily protein | NULL | Bifunctional inhibitor/lipid-transfer protein/seed storage 2S albumin superfamily protein; FUNCTIONS IN: lipid binding; INVOLVED IN: lipid transport; LOCATED IN: endomembrane system; EXPRESSED IN: 22 plant structures; EXPRESSED DURING: 13 growth stages; CONTAINS InterPro DOMAIN/s: Bifunctional inhibitor/plant lipid transfer protein/seed storage (InterPro:IPR016140), Plant lipid transfer protein/seed storage/trypsin-alpha amylase inhibitor (InterPro:IPR003612), Plant lipid transfer protein/hydrophobic protein, helical domain (InterPro:IPR013770); BEST Arabidopsis thaliana protein match is: Bifunctional inhibitor/lipid-transfer protein/seed storage 2S albumin superfamily protein (TAIR:AT3G18280.1) |
| AT1G50010.1 | tubulin alpha-2 chain | Encodes alpha-2,4 tubulin. TUA2 and TUA4 encode identical proteins. The mRNA is cell-to-cell mobile. | tubulin alpha-2 chain (TUA2); FUNCTIONS IN: structural constituent of cytoskeleton; INVOLVED IN: microtubule-based process, response to salt stress; LOCATED IN: tubulin complex, cytosol, cell wall, membrane; EXPRESSED IN: 26 plant structures; EXPRESSED DURING: 15 growth stages; CONTAINS InterPro DOMAIN/s: Alpha tubulin (InterPro:IPR002452), Tubulin (InterPro:IPR000217), Tubulin/FtsZ, GTPase domain (InterPro:IPR003008), Tubulin/FtsZ, N-terminal (InterPro:IPR019746), Tubulin/FtsZ, C-terminal (InterPro:IPR008280), Tubulin, conserved site (InterPro:IPR017975), Tubulin/FtsZ, 2-layer sandwich domain (InterPro:IPR018316); BEST Arabidopsis thaliana protein match is: tubulin alpha-4 chain (TAIR:AT1G04820.1) |

| | | | |
|---|---|---|---|
| AT1G75680.1 | glycosyl hydrolase 9B7 | NULL | glycosyl hydrolase 9B7 (GH9B7); FUNCTIONS IN: hydrolase activity, hydrolyzing O-glycosyl compounds, catalytic activity; INVOLVED IN: carbohydrate metabolic process; LOCATED IN: plasma membrane; EXPRESSED IN: 23 plant structures; EXPRESSED DURING: 13 growth stages; CONTAINS InterPro DOMAIN/s: Six-hairpin glycosidase (InterPro:IPR012341), Glycoside hydrolase, family 9, active site (InterPro:IPR018221), Six-hairpin glycosidase-like (InterPro:IPR008928), Glycoside hydrolase, family 9 (InterPro:IPR001701); BEST Arabidopsis thaliana protein match is: glycosyl hydrolase 9B5 (TAIR:AT1G19940.1) |
| AT2G04780.2 | FASCICLIN-like arabinoogalactan 7 | fasciclin-like arabinogalactan-protein 7 (Fla7) | FASCICLIN-like arabinoogalactan 7 (FLA7); LOCATED IN: anchored to plasma membrane, plasma membrane, anchored to membrane, membrane; EXPRESSED IN: 22 plant structures; EXPRESSED DURING: 13 growth stages; CONTAINS InterPro DOMAIN/s: FAS1 domain (InterPro:IPR000782); BEST Arabidopsis thaliana protein match is: FASCICLIN-like arabinogalactan 6 (TAIR:AT2G20520.1) |
| AT4G00230.1 | xylem serine peptidase 1 | NULL | xylem serine peptidase 1 (XSP1); FUNCTIONS IN: identical protein binding, serine-type endopeptidase activity; INVOLVED IN: proteolysis, negative regulation of catalytic activity; LOCATED IN: cell wall, plant-type cell wall; EXPRESSED IN: 24 plant structures; EXPRESSED DURING: 15 growth stages; CONTAINS InterPro DOMAIN/s: Protease-associated PA (InterPro:IPR003137), Peptidase S8/S53, subtilisin/kexin/sedolisin (InterPro:IPR000209), Peptidase S8, subtilisin-related (InterPro:IPR015500), Peptidase S8/S53, subtilisin, active site (InterPro:IPR022398), Proteinase inhibitor I9, subtilisin propeptide (InterPro:IPR010259); BEST Arabidopsis thaliana protein match is: Subtilisin-like serine endopeptidase family protein (TAIR:AT5G03620.1) |
| AT4G00360.1 | cytochrome P450, family 86, subfamily A, polypeptide 2 | Encodes a member of the CYP86A subfamily of cytochrome p450 genes. Expressed at moderate levels in flowers, leaves, roots and stems. | cytochrome P450, family 86, subfamily A, polypeptide 2 (CYP86A2); CONTAINS InterPro DOMAIN/s: Cytochrome P450 (InterPro:IPR001128), Cytochrome P450, E-class, group I (InterPro:IPR002401), Cytochrome P450, conserved site (InterPro:IPR017972); BEST Arabidopsis thaliana protein match is: cytochrome P450, family 86, subfamily A, polypeptide 4 (TAIR:AT1G01600.1) |

| | | | |
|---|---|---|---|
| AT4G12420.2 | Cupredoxin superfamily protein | Encodes a protein of unknown function involved in directed root tip growth. It is a member of 19-member gene family and is distantly related structurally to the multiple-copper oxidases ascorbate oxidase and laccase, though it lacks the copper-binding domains. The protein is glycosylated and GPI-anchored. It is localized to the plasma membrane and the cell wall. The gene is expressed most strongly in expanding tissues. | SKU5; FUNCTIONS IN: oxidoreductase activity, copper ion binding; INVOLVED IN: cell tip growth; LOCATED IN: plasma membrane, anchored to membrane, plant-type cell wall; EXPRESSED IN: 23 plant structures; EXPRESSED DURING: 13 growth stages; CONTAINS InterPro DOMAIN/s: Multicopper oxidase, type 3 (InterPro:IPR011707), Cupredoxin (InterPro:IPR008972), Multicopper oxidase, type 2 (InterPro:IPR011706), Multicopper oxidase, type 1 (InterPro:IPR001117); BEST Arabidopsis thaliana protein match is: SKU5 similar 2 (TAIR:AT5G51480.1). |

**Table S7** Annotation of putative genes under positive selection between *M. candidum* and *M. sanguineum* .

(a) Leaf

| Gene Name | Other Names | Description | Annotations |
|---|---|---|---|
| AT1G01780.1 | GATA type zinc finger transcription factor family protein | Encodes a member of the Arabidopsis LIM proteins: a family of actin bundlers with distinct expression patterns. WLIM1, WLIM2a, and WLIM2b are widely expressed, whereas PLIM2a, PLIM2b, and PLIM2c are predominantly expressed in pollen. Regulates actin cytoskeleton organization. The mRNA is cell-to-cell mobile. | GATA type zinc finger transcription factor family protein; FUNCTIONS IN: zinc ion binding; INVOLVED IN: biological_process unknown; LOCATED IN: cellular_component unknown; EXPRESSED IN: 23 plant structures; EXPRESSED DURING: 15 growth stages; CONTAINS InterPro DOMAIN/s: Zinc finger, LIM-type (InterPro:IPR001781); BEST Arabidopsis thaliana protein match is: GATA type zinc finger transcription factor family protein (TAIR:AT2G45800.1) |
| AT1G05010.1 | ethylene-forming enzyme | Encodes 1-aminocyclopropane-1-carboxylate oxidase | ethylene-forming enzyme (EFE); FUNCTIONS IN: 1-aminocyclopropane-1-carboxylate oxidase activity; INVOLVED IN: response to fungus, ethylene biosynthetic process; EXPRESSED IN: 22 plant structures; EXPRESSED DURING: 13 growth stages; CONTAINS InterPro DOMAIN/s: Oxoglutarate/iron-dependent oxygenase (InterPro:IPR005123); BEST Arabidopsis thaliana protein match is: 2-oxoglutarate (2OG) and Fe(II)-dependent oxygenase superfamily protein (TAIR:AT1G12010.1) |
| AT1G66230.1 | myb domain protein 20 | Encodes a putative transcription factor (MYB20). | myb domain protein 20 (MYB20); CONTAINS InterPro DOMAIN/s: SANT, DNA-binding (InterPro:IPR001005), Homeodomain-like (InterPro:IPR009057), Myb, DNA-binding (InterPro:IPR014778), HTH transcriptional regulator, Myb-type, DNA-binding (InterPro:IPR017930), Homeodomain-related (InterPro:IPR012287), Myb transcription factor (InterPro:IPR015495); BEST Arabidopsis thaliana protein match is: myb domain protein 43 (TAIR:AT5G16600.1) |
| AT1G67230.1 | little nuclei1 | Encodes a nuclear coiled-coil protein related to the carrot peripheral nuclear protein NMCP1 that is involved in the determination of plant nuclear structure. | LITTLE NUCLEI1 (LINC1); FUNCTIONS IN: molecular_function unknown; INVOLVED IN: nucleus organization; LOCATED IN: cytosol, nucleus; EXPRESSED IN: 25 plant structures; EXPRESSED DURING: 13 growth stages; BEST Arabidopsis thaliana protein match is: nuclear matrix constituent protein-related (TAIR:AT1G13220.2) |

| | | | |
|---|---|---|---|
| AT1G75950.1 | S phase kinase-associated protein 1 | SKP1 is core component of the SCF family of E3 ubiquitin ligases and serves to tether the rest of the complex to an F-box protein, which provides specificity in binding to ubiquitin ligase substrate proteins. Predominately expressed from leptotene to pachytene. Negatively regulates recombination. Interacts with P0, a silencing suppressor protein encoded by poleroviruses by means of a conserved minimal F-box motif. The mRNA is cell-to-cell mobile. | S phase kinase-associated protein 1 (SKP1); FUNCTIONS IN: ubiquitin-protein ligase activity, protein binding; INVOLVED IN: negative regulation of DNA recombination, response to cadmium ion, mitosis, male meiosis, ubiquitin-dependent protein catabolic process; LOCATED IN: in 7 components; EXPRESSED IN: 35 plant structures; EXPRESSED DURING: 15 growth stages; CONTAINS InterPro DOMAIN/s: E3 ubiquitin ligase, SCF complex, Skp subunit (InterPro:IPR016897), SKP1 component, dimerisation (InterPro:IPR016072), SKP1 component (InterPro:IPR001232), BTB/POZ fold (InterPro:IPR011333), SKP1 component, POZ (InterPro:IPR016073); BEST Arabidopsis thaliana protein match is: E3 ubiquitin ligase SCF complex subunit SKP1/ASK1 family protein (TAIR:AT5G42190.1) |
| AT1G78060.1 | Glycosyl hydrolase family protein | NULL | Glycosyl hydrolase family protein; FUNCTIONS IN: hydrolase activity, hydrolyzing O-glycosyl compounds; INVOLVED IN: carbohydrate metabolic process; LOCATED IN: apoplast, cell wall, chloroplast, plant-type cell wall; EXPRESSED IN: 22 plant structures; EXPRESSED DURING: 13 growth stages; CONTAINS InterPro DOMAIN/s: Glycoside hydrolase, family 3, N-terminal (InterPro:IPR001764), Glycoside hydrolase, family 3, C-terminal (InterPro:IPR002772), Glycoside hydrolase, catalytic core (InterPro:IPR017853); BEST Arabidopsis thaliana protein match is: Glycosyl hydrolase family protein (TAIR:AT5G10560.1) |
| AT2G26450.1 | Plant invertase/pectin methylesterase inhibitor superfamily | NULL | Plant invertase/pectin methylesterase inhibitor superfamily; FUNCTIONS IN: enzyme inhibitor activity, pectinesterase activity; INVOLVED IN: cell wall modification; LOCATED IN: cell wall, plant-type cell wall; EXPRESSED IN: 9 plant structures; EXPRESSED DURING: L mature pollen stage, M germinated pollen stage, 4 anthesis, C globular stage, petal differentiation and expansion stage; CONTAINS InterPro DOMAIN/s: Pectinesterase, active site (InterPro:IPR018040), Pectin lyase fold/virulence factor (InterPro:IPR011050), Pectinesterase, catalytic (InterPro:IPR000070), Pectinesterase inhibitor (InterPro:IPR006501), Pectin lyase fold (InterPro:IPR012334); BEST Arabidopsis thaliana protein match is: Plant invertase/pectin methylesterase inhibitor superfamily (TAIR:AT4G33230.1) |

| | | | |
|---|---|---|---|
| AT2G36026.1 | Ovate family protein | NULL | Ovate family protein; FUNCTIONS IN: molecular_function unknown; INVOLVED IN: biological_process unknown; LOCATED IN: cellular_component unknown; CONTAINS InterPro DOMAIN/s: Protein of unknown function DUF623 (InterPro:IPR006458); BEST Arabidopsis thaliana protein match is: ovate family protein 6 (TAIR:AT3G52525.1) |
| AT2G38120.1 | Transmembrane amino acid transporter family protein | Encodes an auxin influx transporter. AUX1 resides at the apical plasma membrane of protophloem cells and at highly dynamic subpopulations of Golgi apparatus and endosomes in all cell types. AUX1 action in the lateral root cap and/or epidermal cells influences lateral root initiation and positioning. Shoot supplied ammonium targets AUX1 and inhibits lateral root emergence. The mRNA is cell-to-cell mobile. | AUXIN RESISTANT 1 (AUX1); CONTAINS InterPro DOMAIN/s: Amino acid transporter, transmembrane (InterPro:IPR013057); BEST Arabidopsis thaliana protein match is: like AUXIN RESISTANT 1 (TAIR:AT5G01240.1) |
| AT3G14460.1 | LRR and NB-ARC domains-containing disease resistance protein | NULL | LRR and NB-ARC domains-containing disease resistance protein; FUNCTIONS IN: ATP binding; INVOLVED IN: apoptosis, defense response; CONTAINS InterPro DOMAIN/s: NB-ARC (InterPro:IPR002182), Leucine-rich repeat (InterPro:IPR001611), Disease resistance protein (InterPro:IPR000767); BEST Arabidopsis thaliana protein match is: NB-ARC domain-containing disease resistance protein (TAIR:AT3G14470.1) |
| AT3G24760.1 | Galactose oxidase/kelch repeat superfamily protein | NULL | Galactose oxidase/kelch repeat superfamily protein; CONTAINS InterPro DOMAIN/s: F-box domain, cyclin-like (InterPro:IPR001810), Galactose oxidase/kelch, beta-propeller (InterPro:IPR011043), Kelch-type beta propeller (InterPro:IPR015915); BEST Arabidopsis thaliana protein match is: Kelch repeat-containing F-box family protein (TAIR:AT1G23390.1) |
| AT3G56340.1 | Ribosomal protein S26e family protein | NULL | Ribosomal protein S26e family protein; FUNCTIONS IN: structural constituent of ribosome; INVOLVED IN: translation, ribosome biogenesis; LOCATED IN: cytosolic small ribosomal subunit, cytosolic ribosome, ribosome, membrane; EXPRESSED IN: 23 plant structures; EXPRESSED DURING: 13 growth stages; CONTAINS InterPro DOMAIN/s: Ribosomal protein S26e (InterPro:IPR000892); BEST Arabidopsis thaliana protein match is: Ribosomal protein S26e family protein (TAIR:AT2G40510.1) |

| | | | |
|---|---|---|---|
| AT3G62100.1 | indole-3-acetic acid inducible 30 | Encodes a member of the Aux/IAA family of proteins implicated in auxin signaling. IAA30 lacks the conserved degron (domain II) found in many family members. IAA30 transcripts are induced by auxin treatment and accumulate preferentially in the quiescent center cells of the root meristem. Overexpression of IAA30 leads to defects in gravitropism, root development, root meristem maintenance, and cotyledon vascular development. Target of LEC2 and AGL15. Promotes somatyic embryogenesis. | Indole-3-acetic acid inducible 30 (IAA30); FUNCTIONS IN: sequence-specific DNA binding transcription factor activity; INVOLVED IN: gravitropism, response to auxin stimulus, response to cyclopentenone, somatic embryogenesis, root development; LOCATED IN: nucleus, chloroplast; EXPRESSED IN: 16 plant structures; EXPRESSED DURING: 6 growth stages; CONTAINS InterPro DOMAIN/s: Aux/IAA-ARF-dimerisation (InterPro:IPR011525), AUX/IAA protein (InterPro:IPR003311); BEST Arabidopsis thaliana protein match is: indole-3-acetic acid inducible 20 (TAIR:AT2G46990.1) |
| AT4G01680.3 | myb domain protein 55 | Encodes a putative transcription factor (MYB55). | Myb domain protein 55 (MYB55); CONTAINS InterPro DOMAIN/s: SANT, DNA-binding (InterPro:IPR001005), Homeodomain-like (InterPro:IPR009057), Myb, DNA-binding (InterPro:IPR014778), HTH transcriptional regulator, Myb-type, DNA-binding (InterPro:IPR017930), Homeodomain-related (InterPro:IPR012287), Myb transcription factor (InterPro:IPR015495); BEST Arabidopsis thaliana protein match is: myb domain protein 61 (TAIR:AT1G09540.1) |
| AT4G02800.1 | NULL | NULL | Unknown protein; FUNCTIONS IN: molecular_function unknown; INVOLVED IN: biological_process unknown; LOCATED IN: chloroplast; EXPRESSED IN: 16 plant structures; EXPRESSED DURING: 9 growth stages; BEST Arabidopsis thaliana protein match is: unknown protein (TAIR:AT5G01970.1) |
| AT4G03500.1 | Ankyrin repeat family protein | NULL | Ankyrin repeat family protein; CONTAINS InterPro DOMAIN/s: Ankyrin repeat-containing domain (InterPro:IPR020683), Ankyrin repeat (InterPro:IPR002110); BEST Arabidopsis thaliana protein match is: Ankyrin repeat family protein (TAIR:AT4G03460.1) |
| AT4G14746.1 | NULL | NULL | CONTAINS InterPro DOMAIN/s: EGF-like (InterPro:IPR006210) |
| AT4G21410.1 | cysteine-rich RLK (RECEPTOR-like protein kinase) 29 | Encodes a cysteine-rich receptor-like protein kinase. | cysteine-rich RLK (RECEPTOR-like protein kinase) 29 (CRK29); FUNCTIONS IN: kinase activity; INVOLVED IN: response to abscisic acid stimulus; LOCATED IN: plasma membrane, vacuole; EXPRESSED IN: 21 plant structures; EXPRESSED DURING: 13 growth stages; CONTAINS InterPro DOMAIN/s: Protein kinase, ATP binding site (InterPro:IPR017441), Protein kinase, catalytic domain (InterPro:IPR000719), Protein of unknown function DUF26 (InterPro:IPR002902), Serine/threonine-protein kinase-like domain (InterPro:IPR017442), Protein kinase-like domain (InterPro:IPR011009), Serine/threonine-protein kinase, active site (InterPro:IPR008271); BEST Arabidopsis thaliana protein match is: cysteine-rich RLK (RECEPTOR-like protein kinase) 28 (TAIR:AT4G21400.1) |

| | | | |
|---|---|---|---|
| AT4G25590.1 | actin depolymerizing factor 7 | NULL | actin depolymerizing factor 7 (ADF7); FUNCTIONS IN: actin binding; INVOLVED IN: biological_process unknown; LOCATED IN: intracellular; EXPRESSED IN: 8 plant structures; EXPRESSED DURING: L mature pollen stage, M germinated pollen stage, 4 anthesis, C globular stage, petal differentiation and expansion stage; CONTAINS InterPro DOMAIN/s: Actin-binding, cofilin/tropomyosin type (InterPro:IPR002108); BEST Arabidopsis thaliana protein match is: actin depolymerizing factor 10 (TAIR:AT5G52360.1) |
| AT5G17680.1 | disease resistance protein (TIR-NBS-LRR class), putative | NULL | disease resistance protein (TIR-NBS-LRR class), putative; FUNCTIONS IN: transmembrane receptor activity, ATP binding; INVOLVED IN: signal transduction, defense response, apoptosis, innate immune response; LOCATED IN: intrinsic to membrane; EXPRESSED IN: 14 plant structures; EXPRESSED DURING: 6 growth stages; CONTAINS InterPro DOMAIN/s: Leucine-rich repeat, typical subtype (InterPro:IPR003591), NB-ARC (InterPro:IPR002182), Leucine-rich repeat (InterPro:IPR001611), Disease resistance protein (InterPro:IPR000767), Toll-Interleukin receptor (InterPro:IPR000157); BEST Arabidopsis thaliana protein match is: Disease resistance protein (TIR-NBS-LRR class) (TAIR:AT5G11250.1) |
| AT5G23570.1 | XS domain-containing protein / XS zinc finger domain-containing protein-related | Required for posttranscriptional gene silencing and natural virus resistance.SGS3 is a member of an 'unknown' protein family. Members of this family have predicted coiled domains suggesting oligomerization and a potential zinc finger domain. Involved in the production of trans-acting siRNAs, through direct or indirect stabilization of cleavage fragments of the primary ta-siRNA transcript. Acts before RDR6 in this pathway. The mRNA is cell-to-cell mobile. | SUPPRESSOR OF GENE SILENCING 3 (SGS3); CONTAINS InterPro DOMAIN/s: Domain of unknown function XS (InterPro:IPR005380), Domain of unknown function, putative Zinc finger, XS/XH (InterPro:IPR005381); BEST Arabidopsis thaliana protein match is: unknown protein (TAIR:AT3G22430.1) |
| AT5G41080.1 | PLC-like phosphodiesterases superfamily protein | Encodes a member of the glycerophosphodiester phosphodiesterase (GDPD) family. | PLC-like phosphodiesterases superfamily protein; FUNCTIONS IN: phosphoric diester hydrolase activity, glycerophosphodiester phosphodiesterase activity; INVOLVED IN: glycerol metabolic process, lipid metabolic process; EXPRESSED IN: 23 plant structures; EXPRESSED DURING: 14 growth stages; CONTAINS InterPro DOMAIN/s: PLC-like phosphodiesterase, TIM beta/alpha-barrel domain (InterPro:IPR017946), Glycerophosphoryl diester phosphodiesterase (InterPro:IPR004129); BEST Arabidopsis thaliana protein match is: senescence-related gene 3 (TAIR:AT3G02040.1) |

| | | | |
|---|---|---|---|
| AT5G48100.1 | Laccase/Diphenol oxidase family protein | Encodes a protein that is similar to laccase-like polyphenol oxidases. Involved in lignin and flavonoids biosynthesis. It has four conserved copper binding domains. Expressed in developing testa, where it colocalizes with the flavonoid end products proanthocyanidins and flavonols. Mutant plants exhibited a delay in developmentally determined browning of the testa, characterized by the pale brown color of seed coat. The tt10 mutant seeds accumulate more epicatechin monomers and more soluble proanthocyanidins than wild-type seeds. Flavonol composition was also affected in tt10 seeds, which exhibited a higher ratio of quercetin rhamnoside monomers versus dimers than wild-type seeds. | TRANSPARENT TESTA 10 (TT10); FUNCTIONS IN: copper ion binding, laccase activity; INVOLVED IN: lignin biosynthetic process, response to copper ion, flavonoid biosynthetic process; LOCATED IN: endomembrane system, apoplast; EXPRESSED IN: 8 plant structures; EXPRESSED DURING: L mature pollen stage, M germinated pollen stage, 4 anthesis, seedling growth, seed development stages; CONTAINS InterPro DOMAIN/s: Multicopper oxidase, type 3 (InterPro:IPR011707), Laccase (InterPro:IPR017761), Multicopper oxidase, type 2 (InterPro:IPR011706), Cupredoxin (InterPro:IPR008972), Multicopper oxidase, copper-binding site (InterPro:IPR002355), Multicopper oxidase, type 1 (InterPro:IPR001117); BEST Arabidopsis thaliana protein match is: laccase 14 (TAIR:AT5G09360.1) |
| AT5G56000.1 | HEAT SHOCK PROTEIN 81.4 | NULL | HEAT SHOCK PROTEIN 81.4 (Hsp81.4); FUNCTIONS IN: unfolded protein binding, ATP binding; INVOLVED IN: protein folding, response to stress; LOCATED IN: cytosol, apoplast, cell wall, nucleus, plasma membrane; EXPRESSED IN: male gametophyte, cultured cell, pollen tube; EXPRESSED DURING: L mature pollen stage, M germinated pollen stage; CONTAINS InterPro DOMAIN/s: Chaperone protein htpG (InterPro:IPR001404), Heat shock protein Hsp90, conserved site (InterPro:IPR019805), Heat shock protein Hsp90, C-terminal (InterPro:IPR020576), Heat shock protein Hsp90, N-terminal (InterPro:IPR020575), Ribosomal protein S5 domain 2-type fold (InterPro:IPR020568), ATPase-like, ATP-binding domain (InterPro:IPR003594); BEST Arabidopsis thaliana protein match is: heat shock protein 81-3 (TAIR:AT5G56010.1) |

(b) Flower petal

| Gene Name | Other Names | Description | Annotations |
|---|---|---|---|
| AT1G02790.1 | polygalacturonase 4 | Encodes a exopolygalacturonase. | polygalacturonase 4 (PGA4); FUNCTIONS IN: polygalacturonase activity; INVOLVED IN: carbohydrate metabolic process; LOCATED IN: endomembrane system; EXPRESSED IN: 14 plant structures; EXPRESSED DURING: 6 growth stages; CONTAINS InterPro DOMAIN/s: Pectin lyase fold/virulence factor (InterPro:IPR011050), Pectin lyase fold (InterPro:IPR012334), Glycoside hydrolase, family 28 (InterPro:IPR000743), Parallel beta-helix repeat (InterPro:IPR006626); BEST Arabidopsis thaliana protein match is: Pectin lyase-like superfamily protein (TAIR:AT3G07830.1) |

| | | | |
|---|---|---|---|
| AT1G11040.1 | HSP40/DnaJ peptide-binding protein | NULL | HSP40/DnaJ peptide-binding protein; FUNCTIONS IN: unfolded protein binding, heat shock protein binding; INVOLVED IN: protein folding; LOCATED IN: cellular_component unknown; EXPRESSED IN: petal, leaf whorl, male gametophyte, flower, pollen tube; EXPRESSED DURING: L mature pollen stage, M germinated pollen stage, 4 anthesis, petal differentiation and expansion stage; CONTAINS InterPro DOMAIN/s: Molecular chaperone, heat shock protein, Hsp40, DnaJ (InterPro:IPR015609), HSP40/DnaJ peptide-binding (InterPro:IPR008971), Chaperone DnaJ, C-terminal (InterPro:IPR002939), Heat shock protein DnaJ (InterPro:IPR003095); BEST Arabidopsis thaliana protein match is: HSP40/DnaJ peptide-binding protein (TAIR:AT1G44160.1) |
| AT1G17370.1 | oligouridylate binding protein 1B | Encodes an RNA-binding protein involved in stress granule formation. Regulated by a transposable element small RNA. | oligouridylate binding protein 1B (UBP1B); FUNCTIONS IN: mRNA 3'-UTR binding; EXPRESSED IN: 23 plant structures; EXPRESSED DURING: 13 growth stages; CONTAINS InterPro DOMAIN/s: RNA recognition motif, RNP-1 (InterPro:IPR000504), Nucleotide-binding, alpha-beta plait (InterPro:IPR012677); BEST Arabidopsis thaliana protein match is: RNA-binding (RRM/RBD/RNP motifs) family protein (TAIR:AT3G14100.1) |
| AT1G69550.1 | disease resistance protein (TIR-NBS-LRR class) | NULL | disease resistance protein (TIR-NBS-LRR class); FUNCTIONS IN: transmembrane receptor activity, nucleoside-triphosphatase activity, nucleotide binding, ATP binding; INVOLVED IN: signal transduction, defense response, apoptosis, innate immune response; LOCATED IN: intrinsic to membrane, endomembrane system; EXPRESSED IN: 21 plant structures; EXPRESSED DURING: 13 growth stages; CONTAINS InterPro DOMAIN/s: ATPase, AAA+ type, core (InterPro:IPR003593), NB-ARC (InterPro:IPR002182), Leucine-rich repeat (InterPro:IPR001611), Disease resistance protein (InterPro:IPR000767), Toll-Interleukin receptor (InterPro:IPR000157); BEST Arabidopsis thaliana protein match is: Disease resistance protein (TIR-NBS-LRR class) (TAIR:AT5G11250.1) |

| | | | |
|---|---|---|---|
| AT1G73590.1 | Auxin efflux carrier family protein | Encodes an auxin efflux carrier involved in shoot and root development. It is involved in the maintenance of embryonic auxin gradients. Loss of function severely affects organ initiation, pin1 mutants are characterised by an inflorescence meristem that does not initiate any flowers, resulting in the formation of a naked inflorescence stem. PIN1 is involved in the determination of leaf shape by actively promoting development of leaf margin serrations. In roots, the protein mainly resides at the basal end of the vascular cells, but weak signals can be detected in the epidermis and the cortex. Expression levels and polarity of this auxin efflux carrier change during primordium development suggesting that cycles of auxin build-up and depletion accompany, and may direct, different stages of primordium development. PIN1 action on plant development does not strictly require function of PGP1 and PGP19 proteins. | PIN-FORMED 1 (PIN1); FUNCTIONS IN: transporter activity; INVOLVED IN: in 14 processes; LOCATED IN: apical part of cell, plasma membrane, membrane, basal plasma membrane, cytoplasm; EXPRESSED IN: 25 plant structures; EXPRESSED DURING: 13 growth stages; CONTAINS InterPro DOMAIN/s: Auxin efflux carrier, subgroup (InterPro:IPR014024), Auxin efflux carrier (InterPro:IPR004776); BEST Arabidopsis thaliana protein match is: Auxin efflux carrier family protein (TAIR:AT1G70940.1) |
| AT1G75950.1 | S phase kinase-associated protein 1 | SKP1 is core component of the SCF family of E3 ubiquitin ligases and serves to tether the rest of the complex to an F-box protein, which provides specificity in binding to ubiquitin ligase substrate proteins. Predominately expressed from leptotene to pachytene. Negatively regulates recombination. Interacts with P0, a silencing suppressor protein encoded by poleroviruses by means of a conserved minimal F-box motif. The mRNA is cell-to-cell mobile. | S phase kinase-associated protein 1 (SKP1); FUNCTIONS IN: ubiquitin-protein ligase activity, protein binding; INVOLVED IN: negative regulation of DNA recombination, response to cadmium ion, mitosis, male meiosis, ubiquitin-dependent protein catabolic process; LOCATED IN: in 7 components; EXPRESSED IN: 35 plant structures; EXPRESSED DURING: 15 growth stages; CONTAINS InterPro DOMAIN/s: E3 ubiquitin ligase, SCF complex, Skp subunit (InterPro:IPR016897), SKP1 component, dimerisation (InterPro:IPR016072), SKP1 component (InterPro:IPR001232), BTB/POZ fold (InterPro:IPR011333), SKP1 component, POZ (InterPro:IPR016073); BEST Arabidopsis thaliana protein match is: E3 ubiquitin ligase SCF complex subunit SKP1/ASK1 family protein (TAIR:AT5G42190.1) |
| AT1G78060.1 | Glycosyl hydrolase family protein | NULL | Glycosyl hydrolase family protein; FUNCTIONS IN: hydrolase activity, hydrolyzing O-glycosyl compounds; INVOLVED IN: carbohydrate metabolic process; LOCATED IN: apoplast, cell wall, chloroplast, plant-type cell wall; EXPRESSED IN: 22 plant structures; EXPRESSED DURING: 13 growth stages; CONTAINS InterPro DOMAIN/s: Glycoside hydrolase, family 3, N-terminal (InterPro:IPR001764), Glycoside hydrolase, family 3, C-terminal (InterPro:IPR002772), Glycoside hydrolase, catalytic core (InterPro:IPR017853); BEST Arabidopsis thaliana protein match is: Glycosyl hydrolase family protein (TAIR:AT5G10560.1) |

| | | | |
|---|---|---|---|
| AT3G07970.1 | Pectin lyase-like superfamily protein | Required for pollen separation during normal development. In qrt mutants, the outer walls of the four meiotic products of the pollen mother cell are fused, and pollen grains are released in tetrads.May be required for cell type-specific pectin degradation. | QUARTET 2 (QRT2); FUNCTIONS IN: polygalacturonase activity; INVOLVED IN: in 6 processes; LOCATED IN: endomembrane system; EXPRESSED IN: 9 plant structures; EXPRESSED DURING: 4 anthesis, petal differentiation and expansion stage; CONTAINS InterPro DOMAIN/s: Pectin lyase fold/virulence factor (InterPro:IPR011050), Glycoside hydrolase, family 28 (InterPro:IPR000743), Parallel beta-helix repeat (InterPro:IPR006626); BEST Arabidopsis thaliana protein match is: Pectin lyase-like superfamily protein (TAIR:AT3G57510.1) |
| AT3G14460.1 | LRR and NB-ARC domains-containing disease resistance protein | NULL | LRR and NB-ARC domains-containing disease resistance protein; FUNCTIONS IN: ATP binding; INVOLVED IN: apoptosis, defense response; CONTAINS InterPro DOMAIN/s: NB-ARC (InterPro:IPR002182), Leucine-rich repeat (InterPro:IPR001611), Disease resistance protein (InterPro:IPR000767); BEST Arabidopsis thaliana protein match is: NB-ARC domain-containing disease resistance protein (TAIR:AT3G14470.1) |
| AT3G21240.1 | 4-coumarate:CoA ligase 2 | Encodes an isoform of 4-coumarate:CoA ligase (4CL), which is involved in the last step of the general phenylpropanoid pathway. The catalytic efficiency was in the following (descending) order: p-coumaric acid, caffeic acid, ferulic acid, 5-OH-ferulic acid and cinnamic acid. At4CL2 was unable to use sinapic acid as substrate. | 4-coumarate:CoA ligase 2 (4CL2); CONTAINS InterPro DOMAIN/s: AMP-binding, conserved site (InterPro:IPR020845), AMP-dependent synthetase/ligase (InterPro:IPR000873); BEST Arabidopsis thaliana protein match is: 4-coumarate:CoA ligase 1 (TAIR:AT1G51680.1) |
| AT3G22970.1 | Protein of unknown function (DUF506) | NULL | Protein of unknown function (DUF506) ; FUNCTIONS IN: molecular_function unknown; INVOLVED IN: biological_process unknown; LOCATED IN: chloroplast; CONTAINS InterPro DOMAIN/s: Protein of unknown function DUF506, plant (InterPro:IPR006502); BEST Arabidopsis thaliana protein match is: Protein of unknown function (DUF506) (TAIR:AT4G14620.1) |
| AT3G24760.1 | Galactose oxidase/kelch repeat superfamily protein | NULL | Galactose oxidase/kelch repeat superfamily protein; CONTAINS InterPro DOMAIN/s: F-box domain, cyclin-like (InterPro:IPR001810), Galactose oxidase/kelch, beta-propeller (InterPro:IPR011043), Kelch-type beta propeller (InterPro:IPR015915); BEST Arabidopsis thaliana protein match is: Kelch repeat-containing F-box family protein (TAIR:AT1G23390.1) |
| AT4G03500.1 | Ankyrin repeat family protein | NULL | Ankyrin repeat family protein; CONTAINS InterPro DOMAIN/s: Ankyrin repeat-containing domain (InterPro:IPR020683), Ankyrin repeat (InterPro:IPR002110); BEST Arabidopsis thaliana protein match is: Ankyrin repeat family protein (TAIR:AT4G03460.1) |

| | | | |
|---|---|---|---|
| AT4G13710.1 | Pectin lyase-like superfamily protein | NULL | Pectin lyase-like superfamily protein; FUNCTIONS IN: lyase activity, pectate lyase activity; INVOLVED IN: biological_process unknown; LOCATED IN: endomembrane system; EXPRESSED IN: 20 plant structures; EXPRESSED DURING: 6 growth stages; CONTAINS InterPro DOMAIN/s: Pectin lyase fold/virulence factor (InterPro:IPR011050), AmbAllergen (InterPro:IPR018082), Pectate lyase/Amb allergen (InterPro:IPR002022), Pectin lyase fold (InterPro:IPR012334); BEST Arabidopsis thaliana protein match is: Pectate lyase family protein (TAIR:AT3G24230.1) |
| AT4G13940.4 | S-adenosyl-L-homocysteine hydrolase | Encodes a S-adenosyl-L-homocysteine hydrolase required for DNA methylation-dependent gene silencing. The mRNA is cell-to-cell mobile. | HOMOLOGY-DEPENDENT GENE SILENCING 1 (HOG1); FUNCTIONS IN: adenosylhomocysteinase activity; INVOLVED IN: methylation-dependent chromatin silencing, one-carbon metabolic process, posttranscriptional gene silencing, embryo development ending in seed dormancy; LOCATED IN: plasma membrane, membrane; EXPRESSED IN: 34 plant structures; EXPRESSED DURING: 16 growth stages; CONTAINS InterPro DOMAIN/s: S-adenosyl-L-homocysteine hydrolase (InterPro:IPR000043), S-adenosyl-L-homocysteine hydrolase, conserved site (InterPro:IPR020082), NAD(P)-binding domain (InterPro:IPR016040), S-adenosyl-L-homocysteine hydrolase, NAD binding (InterPro:IPR015878); BEST Arabidopsis thaliana protein match is: S-adenosyl-l-homocysteine (SAH) hydrolase 2 (TAIR:AT3G23810.1) |
| AT4G25590.1 | actin depolymerizing factor 7 | NULL | actin depolymerizing factor 7 (ADF7); FUNCTIONS IN: actin binding; INVOLVED IN: biological_process unknown; LOCATED IN: intracellular; EXPRESSED IN: 8 plant structures; EXPRESSED DURING: L mature pollen stage, M germinated pollen stage, 4 anthesis, C globular stage, petal differentiation and expansion stage; CONTAINS InterPro DOMAIN/s: Actin-binding, cofilin/tropomyosin type (InterPro:IPR002108); BEST Arabidopsis thaliana protein match is: actin depolymerizing factor 10 (TAIR:AT5G52360.1) |

| | | | |
|---|---|---|---|
| AT4G30440.1 | UDP-D-glucuronate 4-epimerase 1 | Encodes a UDP-D-glucuronate 4-epimerase involved in pectin biosynthesis in the cell wall and affects cell wall integrity and immunity to fungi and bacteria. | UDP-D-glucuronate 4-epimerase 1 (GAE1); FUNCTIONS IN: UDP-glucuronate 4-epimerase activity, catalytic activity; INVOLVED IN: cellular metabolic process, carbohydrate metabolic process, nucleotide-sugar metabolic process, metabolic process; LOCATED IN: cellular_component unknown; EXPRESSED IN: 24 plant structures; EXPRESSED DURING: 15 growth stages; CONTAINS InterPro DOMAIN/s: NAD-dependent epimerase/dehydratase (InterPro:IPR001509), NAD(P)-binding domain (InterPro:IPR016040), Nucleotide sugar epimerase (InterPro:IPR008089); BEST Arabidopsis thaliana protein match is: UDP-D-glucuronate 4-epimerase 2 (TAIR:AT1G02000.1) |
| AT4G33430.1 | BRI1-associated receptor kinase | Leu-rich receptor Serine/threonine protein kinase. Component of BR signaling that interacts with BRI1 in vitro and in vivo to form a heterodimer. Brassinolide-dependent association of BRI1 and BAK1 in vivo. Phosphorylation of both BRI1 and BAK1 on Thr residues was BR dependent. Although BAK1 and BRI1 alone localize in the plasma membrane, when BAK1 and BRI1 are coexpressed, the heterodimer BAK1/BRI1 they form is localized in the endosome. | BRI1-associated receptor kinase (BAK1); FUNCTIONS IN: protein binding, protein serine/threonine kinase activity, protein heterodimerization activity, kinase activity; INVOLVED IN: in 6 processes; LOCATED IN: endosome, plasma membrane, protein complex; EXPRESSED IN: 26 plant structures; EXPRESSED DURING: 13 growth stages; CONTAINS InterPro DOMAIN/s: Protein kinase, ATP binding site (InterPro:IPR017441), Protein kinase, catalytic domain (InterPro:IPR000719), Leucine-rich repeat-containing N-terminal domain, type 2 (InterPro:IPR013210), Leucine-rich repeat (InterPro:IPR001611), Serine/threonine-protein kinase-like domain (InterPro:IPR017442), Protein kinase-like domain (InterPro:IPR011009), Serine/threonine-protein kinase, active site (InterPro:IPR008271); BEST Arabidopsis thaliana protein match is: somatic embryogenesis receptor-like kinase 4 (TAIR:AT2G13790.1) |
| AT4G36630.1 | Vacuolar sorting protein 39 | NULL | EMBRYO DEFECTIVE 2754 (EMB2754); FUNCTIONS IN: small GTPase regulator activity, binding; INVOLVED IN: embryo development ending in seed dormancy; LOCATED IN: cellular_component unknown; EXPRESSED IN: 24 plant structures; EXPRESSED DURING: 15 growth stages; CONTAINS InterPro DOMAIN/s: Citron-like (InterPro:IPR001180), Vacuolar sorting protein 39/Transforming growth factor beta receptor-associated domain 1 (InterPro:IPR019452), Armadillo-type fold (InterPro:IPR016024), Vacuolar sorting protein 39/Transforming growth factor beta receptor-associated domain 2 (InterPro:IPR019453) |

| | | | |
|---|---|---|---|
| AT5G08290.1 | mRNA splicing factor, thioredoxin-like U5 snRNP | Encodes Dim1 homolog. | YELLOW-LEAF-SPECIFIC GENE 8 (YLS8); CONTAINS InterPro DOMAIN/s: Thioredoxin fold (InterPro:IPR012335), mRNA splicing factor, thioredoxin-like U5 snRNP (InterPro:IPR004123), Thioredoxin-like fold (InterPro:IPR012336); BEST Arabidopsis thaliana protein match is: mRNA splicing factor, thioredoxin-like U5 snRNP (TAIR:AT3G24730.1) |
| AT5G15410.2 | Cyclic nucleotide-regulated ion channel family protein | defense, no death' gene (DND1) encodes a mutated cyclic nucleotide-gated cation channel; Same as CNGC2 (article ID 229): Cyclic nucleotide gated channel, activated by cAMP, conducts K+ and other monovalent cations but excludes Na+, does not contain the GYG amino acid sequence found in other channels with this conductivity profile. Conducts Ca2+ into cells which is linked to the generation of NO and the NO signaling pathway involved in the innate immune response to pathogens. CNGC2 could be the key step mediating bulk Ca2+ influx into leaf cells after unloading from the vascular and have no direct roles in the leaf development and HR. | DEFENSE NO DEATH 1 (DND1); CONTAINS InterPro DOMAIN/s: Cyclic nucleotide-binding (InterPro:IPR000595), Cyclic nucleotide-binding-like (InterPro:IPR018490), RmlC-like jelly roll fold (InterPro:IPR014710); BEST Arabidopsis thaliana protein match is: cyclic nucleotide-gated cation channel 4 (TAIR:AT5G54250.2) |
| AT5G16570.1 | glutamine synthetase 1;4 | Encodes a cytosolic glutamine synthetase, the enzyme has high affinity with substrate ammonium | glutamine synthetase 1;4 (GLN1;4); CONTAINS InterPro DOMAIN/s: Glutamine synthetase, catalytic domain (InterPro:IPR008146), Glutamine synthetase, beta-Grasp (InterPro:IPR008147), Glutamine synthetase/guanido kinase, catalytic domain (InterPro:IPR014746); BEST Arabidopsis thaliana protein match is: glutamine synthase clone R1 (TAIR:AT5G37600.1) |
| AT5G17680.1 | disease resistance protein (TIR-NBS-LRR class), putative | NULL | disease resistance protein (TIR-NBS-LRR class), putative; FUNCTIONS IN: transmembrane receptor activity, ATP binding; INVOLVED IN: signal transduction, defense response, apoptosis, innate immune response; LOCATED IN: intrinsic to membrane; EXPRESSED IN: 14 plant structures; EXPRESSED DURING: 6 growth stages; CONTAINS InterPro DOMAIN/s: Leucine-rich repeat, typical subtype (InterPro:IPR003591), NB-ARC (InterPro:IPR002182), Leucine-rich repeat (InterPro:IPR001611), Disease resistance protein (InterPro:IPR000767), Toll-Interleukin receptor (InterPro:IPR000157); BEST Arabidopsis thaliana protein match is: Disease resistance protein (TIR-NBS-LRR class) (TAIR:AT5G11250.1) |
| AT5G23570.1 | XS domain-containing protein / XS zinc finger domain-containing protein-related | Required for posttranscriptional gene silencing and natural virus resistance.SGS3 is a member of an 'unknown' protein family. Members of this family have predicted coiled coiled domains suggesting oligomerization and a potential zinc finger domain. Involved in the production of trans-acting siRNAs, through direct or indirect stabilization of cleavage fragments of the primary ta-siRNA transcript. Acts before RDR6 in this pathway. The mRNA is cell-to-cell mobile. | SUPPRESSOR OF GENE SILENCING 3 (SGS3); CONTAINS InterPro DOMAIN/s: Domain of unknown function XS (InterPro:IPR005380), Domain of unknown function, putative Zinc finger, XS/XH (InterPro:IPR005381); BEST Arabidopsis thaliana protein match is: unknown protein (TAIR:AT3G22430.1) |

| | | | |
|---|---|---|---|
| AT5G24530.1 | 2-oxoglutarate (2OG) and Fe(II)-dependent oxygenase superfamily protein | Encodes a putative 2OG-Fe(II) oxygenase that is defense-associated but required for susceptibility to downy mildew. The mRNA is cell-to-cell mobile. | DOWNY MILDEW RESISTANT 6 (DMR6); FUNCTIONS IN: oxidoreductase activity, acting on paired donors, with incorporation or reduction of molecular oxygen, 2-oxoglutarate as one donor, and incorporation of one atom each of oxygen into both donors, oxidoreductase activity; INVOLVED IN: response to fungus, response to bacterium, flavonoid biosynthetic process; LOCATED IN: cellular_component unknown; EXPRESSED IN: 22 plant structures; EXPRESSED DURING: 13 growth stages; CONTAINS InterPro DOMAIN/s: Oxoglutarate/iron-dependent oxygenase (InterPro:IPR005123); BEST Arabidopsis thaliana protein match is: 2-oxoglutarate (2OG) and Fe(II)-dependent oxygenase superfamily protein (TAIR:AT4G10490.1) |
| AT5G35450.1 | Disease resistance protein (CC-NBS-LRR class) family | NULL | Disease resistance protein (CC-NBS-LRR class) family; FUNCTIONS IN: ATP binding; INVOLVED IN: defense response, apoptosis; LOCATED IN: plasma membrane; EXPRESSED IN: 21 plant structures; EXPRESSED DURING: 10 growth stages; CONTAINS InterPro DOMAIN/s: NB-ARC (InterPro:IPR002182), Disease resistance protein (InterPro:IPR000767); BEST Arabidopsis thaliana protein match is: Disease resistance protein (CC-NBS-LRR class) family (TAIR:AT5G43470.2) |
| AT5G36110.1 | cytochrome P450, family 716, subfamily A, polypeptide 1 | Member of CYP716A | cytochrome P450, family 716, subfamily A, polypeptide 1 (CYP716A1); FUNCTIONS IN: electron carrier activity, monooxygenase activity, iron ion binding, oxygen binding, heme binding; INVOLVED IN: oxidation reduction; LOCATED IN: endomembrane system; CONTAINS InterPro DOMAIN/s: Cytochrome P450 (InterPro:IPR001128), Cytochrome P450, conserved site (InterPro:IPR017972), Cytochrome P450, E-class, group I (InterPro:IPR002401); BEST Arabidopsis thaliana protein match is: cytochrome P450, family 716, subfamily A, polypeptide 2 (TAIR:AT5G36140.1) |
